# Carbene footprinting accurately maps binding sites in protein–ligand and protein–protein interactions

**DOI:** 10.1038/ncomms13288

**Published:** 2016-11-16

**Authors:** Lucio Manzi, Andrew S. Barrow, Daniel Scott, Robert Layfield, Timothy G. Wright, John E. Moses, Neil J. Oldham

**Affiliations:** 1 School of Chemistry, University of Nottingham, University Park, Nottingham NG7 2RD, UK; 2 Queen's Medical Centre, School of Life Sciences, University of Nottingham, Nottingham NG7 2UH, UK

## Abstract

Specific interactions between proteins and their binding partners are fundamental to life processes. The ability to detect protein complexes, and map their sites of binding, is crucial to understanding basic biology at the molecular level. Methods that employ sensitive analytical techniques such as mass spectrometry have the potential to provide valuable insights with very little material and on short time scales. Here we present a differential protein footprinting technique employing an efficient photo-activated probe for use with mass spectrometry. Using this methodology the location of a carbohydrate substrate was accurately mapped to the binding cleft of lysozyme, and in a more complex example, the interactions between a 100 kDa, multi-domain deubiquitinating enzyme, USP5 and a diubiquitin substrate were located to different functional domains. The much improved properties of this probe make carbene footprinting a viable method for rapid and accurate identification of protein binding sites utilizing benign, near-UV photoactivation.

## Abstract (web summary)

Mapping protein-ligand interactions is fundamental to advance the understanding of cellular processes and to develop drug discovery strategies. Here, the authors present a photo-activated probe that allows highly efficient labelling and identification of protein binding sites using mass spectrometry.

## Graphical abstract

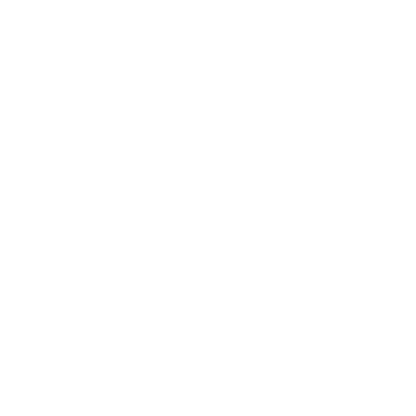

Interactions between proteins, and between proteins and small molecules are at the heart of practically all biochemical processes^1^. Methods that allow detection and mapping of these interactions are consequently essential both in understanding basic biology at the molecular level, and in developing drug discovery strategies based on rational design. Frequently used techniques, such as X-ray crystallography and nuclear magnetic resonance (NMR) spectroscopy deliver high-resolution structural data, but are often time-consuming and require relatively large amounts of material^2^. In contrast, methods that employ mass spectrometry (MS) to detect and map protein interactions are rapid and sensitive, but produce relatively low-resolution data^3^,^4^. MS-based strategies that provide reliable and high-resolution mapping of protein interaction sites are, therefore, highly sought after^5^. Towards this goal, a number of approaches have been employed, including hydrogen-deuterium exchange (HDX), chemical modification and cross-linking, and rapid footprinting strategies such as hydroxyl radical or carbene labelling. HDX, one of the first and most widely used methods, probes the mass shift associated with deuterium uptake^6^. This procedure has provided promising results, particularly with stable complexes, but suffers from two main drawbacks: unwanted (and variable) back-exchange of deuterium under quenching/digestion/analysis conditions^7^, and H/D scrambling under collisional MS/MS activation^8^. The latter problem arises when peptides are fragmented, using thermal activation methods, to gain sub-peptide-level information. Scrambling of the label can be reduced by non-thermal activation^9^, but the general labile nature of the deuterium label complicates data interpretation in HDX experiments. Chemical modification and cross-linking are, again, widely utilized and have been successfully used to map protein structure and interactions^10^,^11^,^12^. The relatively low resolution of the data (the labelling density can be low, and reliant on the chemical reactivity of specific amino acid sidechains), and the often slow rate of chemical reaction leading to labelling/crosslinking, are drawbacks of this approach. For stable complexes this may not be problematic, but for more dynamic systems the temporal resolution afforded by the reaction is limited. Hydroxyl radical labelling is a very promising method for protein footprinting^13^,^14^,^15^, especially in the form of fast photochemical oxidation of proteins (FPOP) developed by the Gross lab^16^, and a similar approach described by Sze and coworkers^17^. Recently, the methodology has been employed to explore the free-energy folding landscape of the bacterial immunity protein Im7 (ref. 18). Although powerful, drawbacks of the strategy include the need to expose the protein to hydrogen peroxide, which may cause oxidative damage, and the use of middle-ultraviolet laser irradiation (typically 266 nm) to photolyse the hydrogen peroxide to produce·OH, which lies within the absorption region of the aromatic amino acids, and hence may be damaging to protein structure. More recently, a FPOP variant has been produced based on iodine photochemical labelling, but this still operates at 266 nm^19^.

Diazirine-based footprinting agents, in contrast, offer the potential to use near-ultraviolet wavelengths (≈350 nm)–outside the absorbance window of amino acids–to generate highly reactive carbenes, which rapidly label the surface of a protein^20^,^21^. It should be noted that this generic strategy is quite distinct from that of photoaffinity labelling, where a specific photoactive ligand is employed, and which therefore requires a different probe for each system studied^22^. The use of methylene gas itself has been reported, but its utility is limited by low solubility in water^20^. Recently, Schriemer and colleagues^23^ described the use of commercially available photoleucine **1** and 4,4-azipentanoic acid for diazirine-based protein carbene footprinting. The former has been used to map the calmodulin-M13 interaction site^24^, and the calcium-induced conformational change in calmodulin^25^. However, high concentrations (100 mM) of the probes were required to obtain reasonable levels of protein labelling. In investigating other protein systems, we found the labelling efficiency of photoleucine **1** to be highly protein dependent, and that labelled peptides exhibited, in some cases, sub-optimal behaviour during MS/MS fragmentation. Despite this, we believed that the carbene-labelling strategy offered considerable promise owing to its use of benign, near-ultraviolet photoactivation by compact low-cost laser systems, and clear mass shifts upon protein labelling. To provide a platform for high-resolution mapping of protein interactions by MS, we have designed and synthesized a bespoke diazirine-based footprinting agent for differential protein labelling experiments. This probe labels proteins with much higher efficiency than photoleucine at both lower concentrations, and lower irradiation times. We have successfully used the probe to map the lysozyme substrate-binding site accurately at high resolution, and identify changes in intramolecular interactions associated with binding. In a second example of the technology we have identified the substrate-binding sites of a large, multi-domain deubiquitinating protease and used the results to clarify uncertainties raised by the X-ray crystal structure.

## Results

### Design and synthesis of a new footprinting probe

To produce an efficient carbene-based probe for protein footprinting, we elected to incorporate an aromatic ring to provide a non-polar surface for interaction with hydrophobic patches on the protein, and an ionic group both to associate with polar amino acid sidechains and to improve water solubility of the probe. The trifluoromethylaryl diazirine **2**, based on a sodium benzoate core was considered an appropriate choice, where the presence of the –CF_3_ group has the benefit of reducing side reactions associated with the diazoisomer^26^. The free acid form of **2** has been synthesized some years previously, for incorporation into photoaffinity probes, but not for use as a footprinting tool^27^. The synthesis of aryldiazirine **2** was achieved following a modified literature method (see the ‘Methods' section, and Supplementary Figs 1–6) in five steps from 4-bromotoluene. It has been shown that, once formed, the lifetime of aryl trifluoromethylcarbenes, such as **3**, is of the order of a few ns (ref. 28), making **2** a promising reagent for fast protein labelling. We note that at the time of publication the free acid of **2** is commercially available.

### Footprinting efficiency of aryldiazirine 2

**Figure 1:**
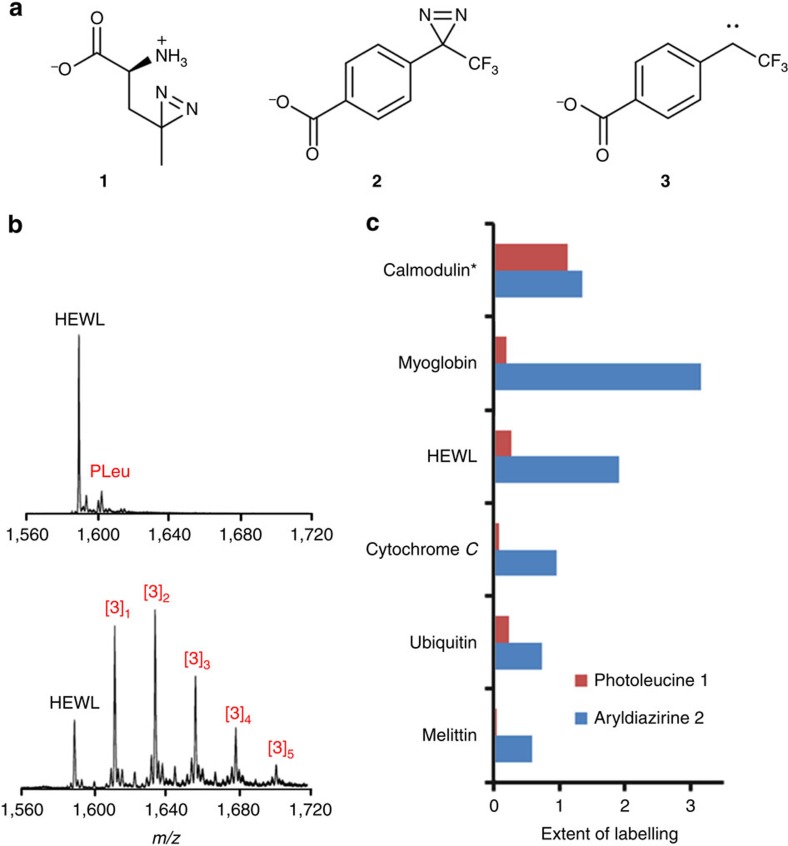
Protein labelling efficiency of diazirine probe 2. (**a**) Structures of the footprinting probes used in this study, (**b**) electrospray ionization-MS of HEWL (9^+^ charge state) showing the high efficiency of labelling achieved with the aryldiazirine **2** (lower spectrum) over photoleucine **1** (upper spectrum), (**c**) extent of labelling of a range of proteins with **1** (100 mM, 16 s irradiation), and **2** (10 mM, 4 s irradiation (*1 s in the case of calmodulin)).

A comparison of the protein labelling efficiency of the aryldiazirine salt **2** with that of photoleucine **1** showed that, for a range of proteins, **2** exhibited far superior properties to the existing probe (Fig. 1). Photo-activated labelling (see the ‘Methods' section) with diazirine **1**, required 100 mM concentrations of the probe and 16 s irradiation times to give modest levels of labelling of the proteins tested, whereas aryldiazirine **2** footprinted with much higher efficiency at only 10 mM and 1–4 s irradiation, following flash freezing (Fig. 1c). This property was found to be reproducible for a range of peptides and proteins tested, making **2** a much more efficient footprinting probe compared with **1** (Fig. 1c and Supplementary Figs 7–12). This may be a result of the increased hydrophobicity of **2**, and its lack of zwitterion character. Indeed, the only protein efficiently labelled by **1** was calmodulin. The degree of labelling for the remaining proteins using **1** was found to be too low for footprinting purposes.

An important consideration when using probes such as **2** to study protein interactions is the potential effect of the diazirine on protein structure. Although we proposed to use a differential approach, where a comparison of labelling in the presence and absence of a binding partner should take into account minor structural effects, we wished to establish that the probe caused no significant perturbation to protein structure. To provide evidence that the presence of **2** in mM quantities was not detrimental to the native structure of the enzyme hen egg white lysozyme (HEWL), enzyme activity assays were performed in the presence and absence of the probe. No change in enzyme activity was detected upon addition of 10 mM **2** to HEWL, indicating that the enzyme's structure was unaffected by the diazirine (Supplementary Fig. 13). These encouraging results led us to footprint differentially HEWL in the presence and absence of a penta-*N*-acetylchitopentaose (NAG_5_) substrate to highlight regions of HEWL masked by NAG_5_. Interactions between enzyme and substrate in this system have been studied in detail, and a large number of high-resolution structures are available^29^, thus it is an excellent model to test the efficacy of **2** as part of a carbene labelling strategy.

### Mapping the NAG_5_ binding site of HEWL

**Figure 2:**
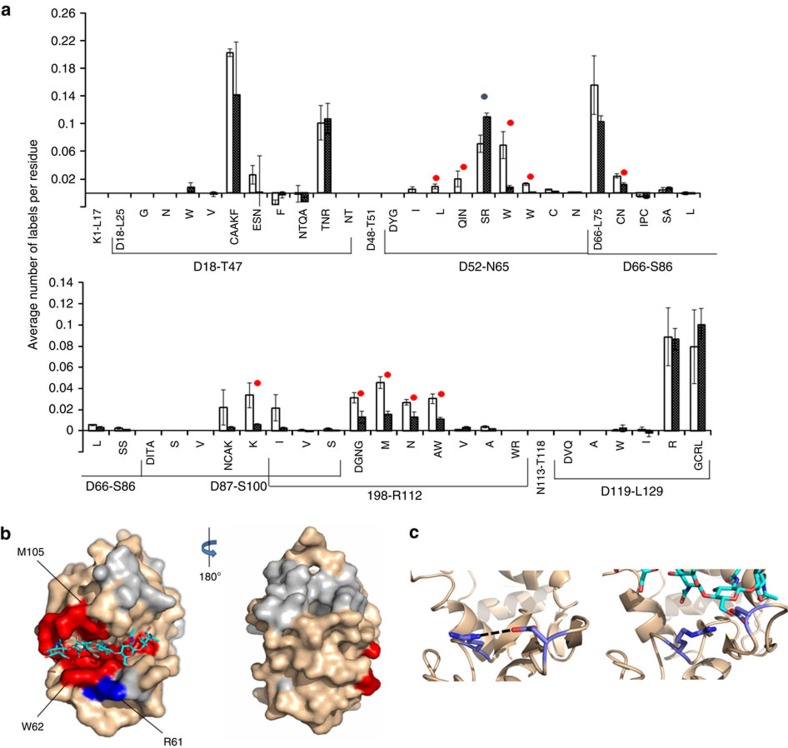
Carbene footprinting of HEWL reveals ligand-binding site. (**a**) Average number of labels per amino acid residue for HEWL footprinted with diazirine **2** in the presence (black bars) and absence (white bars) of NAG_5_. Error bars are±s.d. (*n*=3) and significant differences (Student's *t*-test, *P*<0.05) are highlighted with a red or blue dot. (**b**) Differential footprinting of HEWL by **2** shown as a surface plot. Colour scheme: red=significant masking by NAG_5_, blue=increased labelling in the presence of NAG_5_ wheat=no difference with/without NAG_5_, grey=areas with no peptide coverage. NAG_5_ is shown as a stick diagram in cyan. Structure based on PDB file 1SFB. (**c**) Details of the region around R61 of HEWL in NAG_5_-unbound (left, PDB 1LYZ) and -bound (right, 1SFB) states showing the disruption of the intramolecular interactions on NAG_5_ (cyan) binding and the resulting increased availability of R61 for labelling by **2**.

Differential footprinting of HEWL by photoactivation of **2** was performed in the presence and absence of NAG_5_ (see the ‘Methods' section). Irradiation of the sample following flash freezing had the dual benefit of halting enzyme turn-over and reducing diffusion of the probe. Indeed, we found freezing a necessary step for efficient labelling. Following proteolysis of HEWL with either AspN or trypsin, the resulting peptides (representing 79% sequence coverage) were analysed by liquid chromatography-tandem MS (LC-MS/MS) to quantify the degree of labelling at each position (see the ‘Methods' section, Supplementary Figs 14–26, and Supplementary Tables 1–38). For some peptides, chromatographic separation of isomers derived from labelling at different positions was observed; in these cases, spectra were combined over all signals for each peptide to give an overall average. In general, the probe was found to label basic and hydrophobic residues preferentially, which may be expected from its structure. The results (Fig. 2) showed that the extent of carbene attachment at some residues was significantly (Student's *t*-test, *P*<0.05) reduced in the presence of the NAG_5_ ligand (Fig. 2a). Pleasingly, when mapped onto the surface of HEWL these positions were found to be located in and around the enzyme binding cleft (Fig. 2b), including the well-characterized NAG_n_-interacting site W62. More distal regions of the protein showed no significant difference in labelling in the presence or absence of NAG_5_. Depending upon the efficiency of MS/MS fragmentation, labelling was mapped to single amino acid residue resolution in some regions of the protein. Two AspN peptides, corresponding to K1-L17 and D49-T52 of HEWL were not detected with sufficient intensity to allow accurate determination of labelling.

In addition to the masking of residues in the HEWL binding cleft, two additional structural details were revealed by carbene footprinting with **2**. First, a reproducible and significant increase in labelling at S60/R61 was observed on addition of the NAG_5_ ligand (Fig. 2a,b). This apparently counterintuitive result can be explained by detailed examination of HEWL's structure in the bound and unbound states. In the absence of substrate, R61 participates in an ionic bond with D48 (Fig. 2c). This intramolecular interaction is disrupted upon NAG_5_ binding, making R61 more accessible for labelling by **2**. Thus, carbene footprinting with our probe is able to reveal established subtle structural changes associated with binding events. Second, a clear reduction in labelling was seen at M105, which–although not directly part of the binding cleft itself–is known to be affected by substrate binding. Termed the hydrophobic box, the region of HEWL around M105, is known to become more rigid when a NAG_n_ molecule is bound^30^, which is highly likely to reduce the carbene labelling efficiency at this site.

### Mapping the diubiquitin binding site of USP5

**Figure 3:**
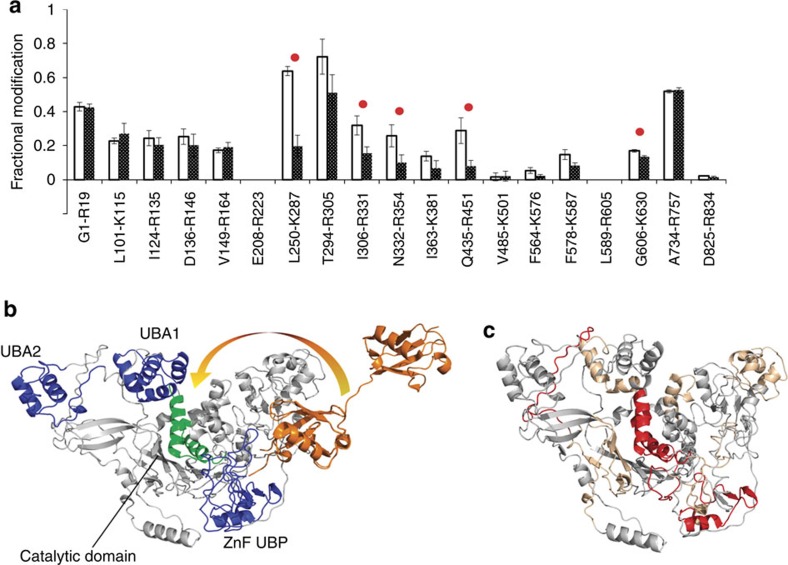
Carbene footprinting of USP5 reveals di-ubiquitin-binding sites. (**a**) Fractional modification (by **2**) of USP5 peptides in the presence (black bars) and absence (white bars) of di-ubiquitin. Error bars are±s.d. (*n*=3) and significant differences (Student's *t*-test, *P*<0.05) are highlighted with a red dot. (**b**) Model of USP5 (based on PDB 3IHP) with di-ubiquitin (orange, PDB 2W9N) bound. The ubiquitin-binding domains are shown in blue, and the catalytic domain in green. The motion required for the ZnF-UBP-bound substrate to approach the catalytic site is indicated by an arrow. (**c**) Model of USP5 (based on PDB 3IHP) showing the locations of the five peptides (red) masked from labelling by di-ubiquitin binding, and their correspondence to the ZnF-UBP and catalytic domains.

Following our successful proof-of-concept study mapping the model HEWL-NAG_5_ protein–ligand system, we sought to apply carbene footprinting to a less well characterized, and more complex protein interaction. The deubiquitinating enzyme ubiquitin specific protease 5 (USP5), of current interest in our laboratory, provides an excellent example of such a protein. The enzyme is responsible for the disassembly of unanchored poly-ubiquitin (poly-Ub) chains^31^, which are isopeptide-linked polymers of ubiquitin that arise in the cell during proteasomal degradation of poly-Ub modified proteins, or via *de novo* assembly. Recent work has identified important physiological roles for unanchored poly-Ub in regulating the 26S proteasome^32^, acting as second messengers in NF-κB signalling^33^, and controlling innate immune response signalling^34^. As a result, USP5 has attracted considerable interest as a potential drug target. This 100 kDa monomeric, multi-domain cysteine protease has three canonical ubiquitin-binding domains: two ubiquitin associated (UBA) domains and a high-affinity Zn-finger ubiquitin-binding protein (ZnF-UBP) domain (Fig. 3b)^35^. The latter possesses a deep binding pocket to accommodate the free C-terminus of the proximal Ub moiety within unanchored poly-Ub chains (a key recognition feature), whilst the former are believed to bind more distal Ub subunits in longer chains. USP5's active site, which is located between these domains and also has an affinity for Ub, cleaves the Ub–Ub isopeptide bond proximal to the C-terminus. A recent crystal structure of USP5 has raised a number of questions about this mechanism: in particular it shows the enzyme in a form where the ZnF-UBP domain is pointing away from the active site^35^. This orientation would prevent a poly-Ub substrate (or even diubiquitin, di-Ub) from interacting with both regions simultaneously (Fig. 3b), and would disfavour the required avidity effects that underlie poly-Ub recognition by the UBDs. USP5 is a dynamic protein, with several flexible linker regions, and we postulated that the ZnF-UBP domain may move relative to the active site to deliver the substrate. To test this hypothesis, footprinting using diazirine **2** was performed on the catalytically inactive C335A USP5, both in the presence and absence of one equivalent of K48-linked di-Ub. Following irradiation and subsequent trypsin digestion, the differential labelling pattern of USP5 was quantified by LC-MS (see the ‘Methods' section, Supplementary Figs 27–85 and Supplementary Tables 39–41 for details). Given the large sizes of the enzyme and substrate (ca. 100 kDa and 17 kDa, respectively), peptide-level analysis was found to be more than adequate to identify binding patches on the former. Figure 3a shows the amount of labelling on each detected USP5 peptide with and without the addition of di-Ub. Large (»10%) and statistically significant differences in labelling were observed for some peptides, and these results mapped on to the X-ray structure of USP5 (Fig. 3c). The data clearly show reduced labelling on the high-affinity ZnF-UBP domain and, importantly, on the active site domain, which is a biologically rational result. Since previous work by us and others has shown that USP5 binds di-Ub with a stoichiometry of 1:1 (refs 31, 36), this result strongly suggests that the protein undergoes a conformational change allowing di-Ub to bind both at the ZnF-UBP and the catalytic site (in contrast to the conformation seen in the X-ray structure). This proposal is supported by mutagenesis and isothermal titration calorimetry findings that show cooperative binding of di-Ub to both the high-affinity ZnF-UBP site and the lower affinity catalytic domain^31^. In addition to labelling differences in these two regions of USP5, a very small (5%), but statistically significant, masking of peptide G606-K630 was seen. This corresponds to a flexible loop adjacent to the two UBA domains, and may reflect conformational change associated with substrate binding. Although the differential nature of the experimental design means that differences in the data should depend upon the presence of the binding partner alone, as with HEWL (see above), we tested against any detrimental effects of 10 mM **2** on the structure of USP5 using an enzyme assay (Supplementary Fig. 68). No deterioration of the protein activity was seen, which indicated the preservation of native protein structure in the presence of probe **2**.

## Discussion

Methods that permit high-resolution mapping of protein interaction sites with improved throughput and sensitivity are highly sought after. We have discovered and demonstrated that differential carbene footprinting with diazirine **2**, in the presence and absence of a binding partner, together with MS-based analysis can provide reliable, high-quality data for both protein-small molecule ligand, and protein–protein systems. Using a bespoke, highly reactive carbene label, this approach has the benefit of fast, efficient and irreversible labelling. These properties confer significant advantages over: HDX, where back-exchange can complicate interpretation; covalent labelling, where slow reaction rates and specific reactivity can give poor spatial and temporal resolution; and photoleucine-based carbene footprinting, which requires high probe concentrations for acceptable levels of labelling. Hydroxyl radical footprinting, and FPOP in particular, is a very promising technique, but does require exposure of the protein to potentially damaging hydrogen peroxide and shorter wavelength ultraviolet radiation. Diazirine **2** appears benign until photoactivation, which occurs rapidly at near-ultraviolet wavelengths (typically ≈350 nm), and its structural features make it a highly efficient labelling reagent. It was not designed to be an accurate measure of solvent accessibility of a protein, but rather for use in a differential, ±binding partner assay. Once formed, the carbene has a lifetime of a few ns, reacting with either protein, buffer or water molecules. By conducting labelling experiments on flash frozen samples we were able to reduce diffusion effects^23^,^24^, and hence it was thought likely that the sites of protein labelling were reflective of preferential diazirine **2**-amino acid sidechain interactions, rather than the inherent reactivity profile of the carbene. This notion was supported by the amino acid-labelling distribution of the hydrophobic and anionic probe **2** (on both HEWL and USP5), which generally favoured hydrophobic and basic (cationic) residues. The spin state of aryltrifluoromethyl carbenes, such as **3**, is relatively complicated; *p*-toluyl(trifluoromethyl)carbene, for example, has a triplet ground state, but it principally reacts through its low-lying singlet state^28^. This property allows aryltrifluoromethyl carbenes to insert into the C–H bonds of non-polar amino acid sidechains, as well as the X–H bonds (where X=O, N, S) found on polar amino acid sidechains, and hence expands their utility in protein labelling.

The binding of carbohydrate substrates, such as NAG_*n*_ oligomers, to HEWL has been studied in detail over the last 50 years, and the precise protein–ligand interactions are well described. The importance of W62, for example, in making van der Waals contacts with a sugar ring in the oligomeric substrate has been demonstrated by X-ray and NMR studies^37^. This residue is highly masked in our differential carbene labelling measurements, as are nearby Q57, I58 and N59, which also lie within the binding cleft. In contrast, R60, positioned between these groups of residues, exhibits an increase in both labelling and solvent exposure upon substrate binding (see the ‘Results' section). These findings are in good agreement with X-ray data, and highlight the accuracy and resolution of the footprinting technique applied to this system.

In contrast, the interactions between USP5 and its poly-Ub substrates are more complex and less well studied than those of lysozyme. USP5 is a large, dynamic enzyme with specific Ub-binding regions in addition to the active-site domain itself. The ZnF-UBP domain has a particularly high affinity for the C-terminus of Ub (ref. 35), and this interaction is key in the recognition of unanchored poly-Ub chains over anchored forms. A recent X-ray crystal structure of USP5 shows the ZnF-UBP domain pointing away from the active site in a manner seemingly incompatible with substrate delivery. ZnF-UBP being flanked by flexible loop regions, can show significant domain motion, and potentially enable it to assist in substrate delivery to the active site. The carbene footprinting results described here show that peptides found on both the ZnF-UBP and catalytic domains of inactivated USP5 are masked from labelling on addition of K48-linked di-Ub. Since the stoichiometry of enzyme-substrate binding is known to be 1:1 (refs 31, 36, 38), and isothermal titration calorimetry data show cooperative binding of the proximal and distal units of di-Ub at the ZnF-UBP and catalytic site, respectively, this finding provides further evidence for an alternative conformation of USP5 where the ZnF-UBP binding pocket is in close spatial proximity to the active site such that di-Ub is bound at both sites. Thus, the conformation of USP5 seen in the crystal structure may represent a form different from the catalytically active species. This finding shows that carbene protein footprinting can be used to provide important insights into complex systems.

In summary, we have shown that differential carbene labelling of proteins with diazirine **2** in the presence and absence of small molecule or protein-binding partners allows high-quality mapping of interaction sites.

## Methods

### Synthesis of diazirine probe 2

Unless otherwise stated, reactions were carried out in flame-dried glassware, under an atmosphere of argon with magnetic stirring. Tetrahydrofuran (THF) was freshly distilled over sodium and benzophenone; dichloromethane (DCM) was freshly distilled over CaH_2_ under a nitrogen atmosphere. NEt_3_ was distilled from CaH_2_ and stored over 4 Å molecular sieves. All other reagents were obtained from commercial sources and used without further purification. Reactions were monitored using thin layer chromatography, and visualized using ultraviolet light and stained using a basic KMnO_4_ (potassium permanganate) solution. Column chromatography was performed using Merck silica gel 60 as the stationary phase. Petrol refers to petroleum spirit (b.p. 40–60 °C).

NMR spectra (^1^H, ^13^C and ^19^F) were recorded on Bruker AV400 (400 MHz), DPX400 (400 MHz), AV3400 (400 MHz) and DPX300 (300 MHz) spectrometers, in CDCl_3_, DMSO-D^6^ or CD_3_OD. Infrared spectra were obtained using a Bruker Tensor 27 FT-IR spectrophotometer as a solution in CHCl_3_, with the peaks recorded as υ_max_ (cm^−1^) or using an attenuated total reflectance (ATR) adaptor for the Nicolet IsT Spectrometer. High-resolution mass spectra (HRMS) were obtained using a Bruker MicroTOF time of flight mass spectrometer operating in electrospray ionization mode, or a Fisons AutoSpec magnetic sector mass spectrometer in electron impact ionization (EI) mode. Melting point data was collected using a Stuart SMP3. Spectral data showed agreement with the literature^27^.

### 2,2,2-trifluoro-1-(*p*-tolyl)ethanone 4


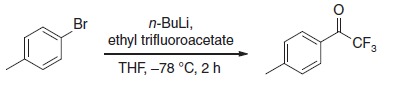


To a solution of 4-bromotoluene (3.00 g, 17.5 mmol) in anhydrous THF (20 ml) at −78 °C was added *n*-BuLi (1.50 M in hexane, 12.3 ml and 18.4 mmol) and the solution stirred for 15 min. Ethyl trifluoroacetate (2.30 ml, 19.3 mmol) was then added dropwise over 5 min, the solution slowly warmed to room temperature over 2 h before addition of 2 M HCl_(aq)_ (10 ml). Volatiles were removed under reduced pressure, before the product was extracted into the organic layer of a work up between EtOAc (50 ml) and 2 M HCl_(aq)_ (50 ml). The organic layer was dried over MgSO_4_, concentrated under reduced pressure to yield a colourless oil, which was used without further purification. A separate reaction was purified for analytical data.

δ_H_ (CDCl_3_, 400 MHz): 7.97–8.00 (2H, m), 7.34–7.38 (2H, m), 2.47 (3H, s); δ_**C**_(CDCl_3_, 101 MHz): 180.1 (q, *J*=35 Hz), 147.0, 130.3, 129.8, 127.5, 116.8 (q, *J*=291 Hz), 21.9; δ_F_ (CDCl_3_, 377 MHz): −71.3; υ_max_ (cm^−1^): 1,714, 1,608, 1,176, 939.

### 2,2,2-trifluoro-1-(*p*-tolyl)ethanone oxime 5


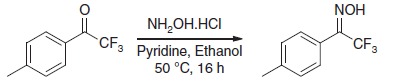


To a solution of crude 2,2,2-trifluoro-1-(*p*-tolyl)ethanone (from the previous step) in pyridine (10 ml) and ethanol (5 ml) was added hydroxylamine hydrochloride (1.22 g, 17.5 mmol). The reaction mixture was heated at 50 °C for 16 h, cooled and diluted with EtOAc (100 ml), and the organic phase washed with 2 M HCl_(aq)_ (50 ml), dried over MgSO_4_, and concentrated under reduced pressure. Purification by column chromatography (5% EtOAc/Petrol) yielded the title compound as a white solid (2.77 g, 78% over two steps). m.p. 77–80 °C (ethanol).

δ_H_ (CDCl_3_, 400 MHz): 9.61 (1H, s), 7.50–7.55 (2H, m), 7.32–7.36 (2H, m), 2.45 (3H, s); δ_C_ (CDCl_3_, 101 MHz): 147.5 (q, *J*=32 Hz), 141.2, 129.3, 128.6, 122.8, 120.7 (q, *J=*274 Hz), 21.4; δ_F_ (CDCl_3_, 377 MHz): −66.6; υ_max_ (cm^−1^): 3,561, 1,612, 1,516, 1,381; HRMS (EI): 203.0551 [M^+^] found, C_9_H_8_F_3_NO_2_ requires 203.0558.

### 2,2,2-trifluoro-1-(*p*-tolyl)ethanone *O*-tosyl oxime 6


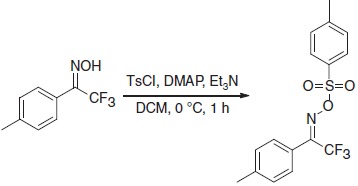


To a solution of 2,2,2-trifluoro-1-(*p*-tolyl)ethanone oxime (1.30 g, 6.40 mmol) and 4-(dimethylamino)pyridine (DMAP) (39 mg, 0.32 mmol) in anhydrous dichloromethane (DCM; 30 ml) was added triethylamine (2.23 ml, 16.0 mmol) followed by tosyl chloride (1.46 g, 7.70 mmol) at 0 °C. The reaction mixture was stirred at room temperature for 1 h before the organic layer was washed with H_2_O, dried over MgSO_4_ and concentrated under reduced pressure. Purification by column chromatography (5% EtOAc/Petrol) yielded the title compound as a white solid (2.21 g, 97%). m.p. 101–103 °C (Et_2_O/hexane).

δ_H_ (CDCl_3_, 400 MHz): 7.87–7.93 (2H, m), 7.20–7.42 (6H, m), 2.49 (3H, s), 2.41 (3H, s); δ_C_(CDCl_3_, 101 MHz): 154.0 (q, *J*=33 Hz), 146.1, 142.5, 131.3, 129.9, 129.5, 129.3, 128.5, 121.7, 119.7 (q, *J*=278 Hz), 21.8, 21.6; δ_F_(CDCl_3_, 377 MHz): −66.6; υ_max_ (cm^−1^): 3,043, 2,926, 1,598, 1,389; HRMS (electrospray ionization): 380.0546 found, C_16_H_14_F_3_NNaO_3_S (M+Na) requires 380.0539.

### 3-(*p*-tolyl)-3-(trifluoromethyl)diaziridine 7


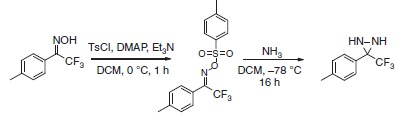


To a solution of 2,2,2-trifluoro-1-(*p*-tolyl)ethanone oxime (**5**, 2.50 g, 12.3 mmol) and DMAP (75 mg, 0.62 mmol) in anhydrous dichloromethane (30 ml) was added triethylamine (4.29 ml, 30.8 mmol) followed by tosyl chloride (2.82 g, 14.80 mmol) at 0 °C. The reaction mixture was stirred at room temperature for 1 h, then the organic layer was washed with H_2_O (50 ml), dried over MgSO_4_ and concentrated under reduced pressure. The crude tosylate **6** was then dissolved in anhydrous dichloromethane (3 ml), added to liquid nitrogen (3 ml) at −78 °C in a microwave vial, sealed and stirred for 16 h.

The microwave vial was de-capped, excess ammonia evaporated, and the residue diluted in dichloromethane (50 ml). The organic phase was washed with H_2_O (50 ml), dried over MgSO_4_ and concentrated under reduced pressure. Purification by column chromatography (20% EtOAc/Petrol) yielded the title compound as a white solid (1.40 g, 56%). m.p. 42–44 °C (hexane).

δ_H_(CDCl_3_, 300 MHz): 7.48–7.53 (2H, m), 7.20–7.26 (2H, m), 2.77 (1H, d, *J*=8.6 Hz), 2.39 (3H, s), 2.19 (1H, d, *J*=8.6 Hz); δ_C_(CDCl_3_, 75 MHz): 140.3, 129.4, 129.1, 128.0, 123.6 (q, *J*=278 Hz), 57.9 (q, *J*=36 Hz), 21.3; δ_F_(CDCl_3_, 282 MHz): −75.7; υ_max_ (cm^−1^): 3,282, 3,009, 1,731, 1,602, 1,394; HRMS (EI): 202.0719 [M^+·^] found, C_9_H_9_F_3_N_2_ requires 202.0718.

### 4-(3-(trifluoromethyl)-3*H*-diazirin-3-yl)benzoic acid 8


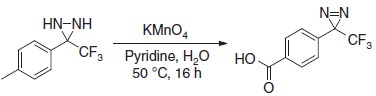


To a homogenous solution of 3-(*p*-tolyl)-3-(trifluoromethyl)-3*H*-diaziridine (**7**, 600 mg, 2.97 mmol) in pyridine (10 ml) and H_2_O (10 ml) was added KMnO_4_ (1.90 g, 11.9 mmol) and the reaction stirred at 50 °C for 16 h. The solution was cooled, diluted with H_2_O (10 ml) and acidified with 2 M HCl_(aq)_ (50 ml) before the addition of 10% sodium bisulfite_(aq)_ (20 ml). The product was extracted into Et_2_O (50 ml), before the organic layer was separated, basified with 2 M NaOH_(aq)_ (60 ml), extracted into aqueous layer before being acidified once more and extracted into Et_2_O (100 ml). The product was dried over MgSO_4_ and concentrated under reduced pressure to yield the title compound **8** as a white solid (200 mg, 29%). m.p. 121–122 °C (Et_2_O/hexane).

δ_H_ (DMSO-d^6^, 400 MHz): 8.02–8.07 (2H, m), 7.38–7.42 (2H, m); δ_C_ (DMSO-d^6^, 101 MHz): 166.3, 132.4, 131.9, 130.1, 126.6, 121.7 (q, *J*=275 Hz), 28.1 (q, *J*=40 Hz); δ_F_ (DMSO-d^6^, 377 MHz): −64.4; υ_max_ (cm^−1^): 3,513, 3,061, 1,737, 1,700 and 1,611.

### Sodium 4-(3-(trifluoromethyl)-3*H*-diazirin-3-yl)benzoate 2


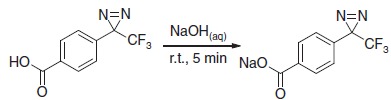


To a solution of sodium hydroxide (16 mg, 0.39 mmol) in H_2_O (1 ml) was added 4-(3-(trifluoromethyl)-3*H*-diazirin-3-yl)benzoic acid (**8**, 100 mg, 0.43 mmol), and the suspension stirred for 5 min. The suspension was filtered, and the filtrate concentrated under reduced pressure to yield the title compound as a white solid (98 mg, 89%).

δ_H_ (CD_3_OD, 400 MHz): 7.98–8.02 (2H, m), 7.20–7.25 (2H, m); δ_C_ (CD_3_OD, 101 MHz): 174.0, 141.1, 131.7, 131.0, 127.0, 123.8 (q, *J*=274 Hz), 29.7 (q, *J*=40 Hz); δ_F_ (CD_3_OD, 282 MHz): −66.9; υ_max_ (cm^−1^): 1,592, 1,546, 1,418, 1,343, 1,144, 942 and 777.

### Protein footprinting

HEWL, penta-*N*-acetylchitopentaose (NAG_5_), dithiotreithol (DTT), iodoacetamide, calmodulin, equine cytochrome *c*, horse heart myoglobin, melittin and ubiquitin were purchased from Sigma-Aldrich (Poole, UK). Trypsin and AspN were obtained from Promega (Southhampton, UK). Lys48 poly-ubiquitin ladder, wild-type USP5 and Lys48 diubiquitin were purchased from Boston Biochem (Cambridge, MA, USA). C335A USP5 was expressed and purified as previously described^34^. Photoleucine **1** was purchased from Thermo Fisher Scientific, Loughborough, UK, and aryldiazirine **2** synthesized as described above.

### Photochemical labelling of lysozyme

An aqueous solution of lysozyme (160 μM) in 25 mM ammonium acetate or 20 mM Tris/150 mM NaCl was mixed with an equal volume of a 20 mM solution of the diazirine labelling agent (**1** or **2**) in 20 mM Tris/150 mM NaCl, to give a final label concentration of 10 mM. The mixture was left equilibrating for 5 min at room temperature. Aliquots (6 μl) of this solution were placed in ‘11 mm PP vial crimp/snap 250 ul' sample vials (Thermo Fisher Scientific). To avoid enzymatic cleavage of the pentasaccharide substrate, NAG_5_ was added to the mixture, to a final concentration of 80 μM, just before snap-freezing the samples in liquid nitrogen (77 K). Freezing was found to be important for efficient and reproducible labelling. For lysozyme footprinting in the absence of substrate, the NAG_5_ solution was substituted by an equal volume of buffer. The labelling reaction was initiated by irradiation (16 s) of the mixture using a Spectra Physics Explorer 349 laser (an actively Q-switched Nd:YLF laser operating at 349 nm, with a repetition frequency of 1,000 Hz, and a pulse energy of 125 μJ, Newport, Didcot, UK). The laser beam was directed into the open top of the sample vial using a small 45° mirror.

*Photochemical labelling of C335A USP5*. A solution of C335A USP5, Lys48 di-ubiquitin (30 μM each, 10 mM ammonium acetate, or 20 mM Tris/150 mM NaCl) and diazirine **2** (in 20 mM Tris/150 mM NaCl, to a final concentration of 10 mM) was snap-frozen and irradiated at 349 nm as described for lysozyme.

*Labelling efficiency*. Solutions of calmodulin, myoglobin, cytochrome *c*, ubiquitin or melittin (70 μM in 20 mM Tris/150 mM NaCl) and photoleucine **1** (100 mM in 20 mM Tris/150 mM NaCl) or diazrine **2** (10 mM in 20 mM Tris/150 NaCl) were snap-frozen and irradiated at 349 nm for 4 s in the case of diazirine **2** (except calmodulin, which required only 1 s), and 15 s in the case of photoleucine **1**. Samples were diluted to 5 μM with mobile phase A (H_2_O/acetonitrile 95/5+0.1% formic acid) and analysed directly by LC-MS.

*Protein digestion*. Following separation by SDS–polyacrylamide gel electrophoresis (SDS–PAGE; 15% acrylamide), protein bands were excised, reduced (DTT, 10 mM in 100 mM ammonium bicarbonate), alkylated (iodoacetamide, 55 mM in 10 mM ammonium bicarbonate) and digested at 37 °C with either trypsin (1:15 protease/protein ratio in 10 mM ammonium bicarbonate) or AspN (1:70 protease/protein ratio in 5 mM Tris-HCl) for 16 h (ref. 39). Formic acid (0.5 μl) was added to the supernatant (50 μl) to inactivate the proteases. The resulting solutions were analysed by LC/MS without further dilution.

*LC-MS analysis*. Measurement of intact mono-ubiquitin, lysozyme or protein digests was carried out on a Dionex Ultimate 3000 Nano LC (Dionex, Camberley, UK) system using a commercially available C4 trapping column (Dionex) and a custom packed WP C4 column (5μm, 100 Å, Phenomenex, Macclesfield, UK) with a Picofrit tip (75 μM i.d. × 150 mm, new objective, supplied by Aquilant, Basingstoke, UK). The mobile phases A and B consisted of 95/5 water/acetonitrile (v/v) and 5/95 water/acetonitrile (v/v), respectively, and both contained 0.1% formic acid. The samples (1 μl) were injected in load-trapping mode. Peptides were eluted using a 30 min linear gradient of mobile phase B from 0–70% at a flow rate of 0.3 μl min^−1^ followed by column equilibration. The high-performance liquid chromatography system was coupled to a Thermo Scientific (Hemel Hempstead, UK) LTQ FT Ultra mass spectrometer equipped with a commercial nanoelectrospray ionization source. A 1.7 kV voltage was applied to a coated PicoTip emitter (New Objective). The capillary temperature was set at 275 °C, with inner capillary voltage value set on 37 V and tube lens value of 145 V. Spectra were acquired in positive ion mode over a 400–2,000 *m/z* range at a nominal resolution of 100,000 (at *m/z*=400). The instrument was controlled by Xcalibur software (Thermo Fisher). Identification of USP5 peptides was conducted using an automated data-dependent acquisition mode, followed by manual examination of the raw data. For data-dependent acquisition the four most intense ions for each scan were isolated within a window of 8 Th and subjected to collision induced dissociation (CID) using a nominal energy of 35.0. Signals with +1 charge state were rejected. The data were searched against a custom database including the C335A USP5 sequence using Bioworks software (Thermo Fisher Scientific).

The proportion of labelling at the peptide level was determined by integrating the signals for each labelled and unlabelled peptide ion. Partial, or even complete chromatographic separation was seen for some labelling isomers (based on the position of the label along the peptide chain), and spectra were combined across the entire peak or set of peaks to ensure that the quantities of all labelled forms were included in the subsequent calculations. CID experiments for residue-level labelling identification/quantitation were performed at a nominal energy of 15.0. Each manually selected labelled precursor ion was isolated within a window of 10.0 Th (although the relatively wide isolation window lead in one case to co-isolation of another peptide, this did not impact upon the precision of the quantitation, and the associated improvement in total signal was found to be beneficial) and the activation time was set at 30 ms with an activation *Q*-value of 0.250. The scans for each labelled peptide were combined to give an average spectrum containing labelled and unlabelled fragments.

Data analysis: the fractional modification per peptide, and the average number of labels per residue were determined using the approach described by Jumper *et al*.^24^ (see Supplementary Methods) and corroborated using quantification by parallel reaction monitoring. Protein structures were displayed using PYMOL.

*Lysozyme activity assay*. Cell suspensions of *Micrococcus lysodeikticus* ATCC (Sigma-Aldrich, Poole, UK, 1 ml, 0.01% in 60 mM potassium phosphate buffer, pH 6.2) were equilibrated in a Multiskan Go microplate spectrophotometer (Thermo Scientific) at 25 °C for 5 min with or without the presence of 10 mM diazirine **2**. A solution of lysozyme (50 μl, 0.1 mg ml^−1^) was added to each suspension, and the decrease in absorbance at 450 nm was measured every 60 s for a total of 10 min. As a control, a cell suspension without the addition of either lysozyme or **2** was similarly monitored.

*USP5 activity assay*. The deubiquitination assay was performed under conditions (1:4 enzyme: substrate molar ratio) previously used by Komander *et al*.^40^ to uncover poly-Ub linkage selectivity of different deubiquitinating enzymes. Briefly, USP5 (Boston Biochem, Cambridge, MA) was diluted to 0.2 mg ml^−1^ in 10 × deubiquitinating buffer (500 mM Tris (pH 7.5), 1500, mM NaCl, 100 mM DTT), to yield a 1 × deubiquitinating buffer and pre-incubated at room temperature for 10 min. 10 μl of the pre-incubated USP5, in the presence or absence of 3 μl of aryldiazirine **2** (100 mM), was then mixed with 3 μg of K63-linked tetraubiquitin (Boston Biochem, Cambridge, MA), 3 μl of 10 × deubiquitinating buffer and made up to 30 μl with water. 6 μl aliquots were removed at the indicated time points and quenched via the addition of 9 μl of SDS–PAGE gel application buffer (0.15 M Tris, 8 M Urea, 2.5% (w/v) SDS, 20% v/v glycerol, 10% (w/v) 2-mercaptoethanol, 3% (w/v) DTT, 0.1% (w/v) bromophenol blue, pH 6.8 (HCl)). 50% of each sample was resolved on a 5–20% SDS–PAGE, western blotted and visualized with anti-ubiquitin (VU-1, Life sensors, Malvern, PA) for the presence of poly-Ub disassembly. Densitometric analysis of the indicated tetraubiquitin band (Supplementary Fig. 17A) was completed using ImageJ software^41^, to quantify the disappearance of tetraubiquitin over the time course in the presence and absence of aryldiazirine **2**.

### Data Availability

Data supporting the findings of this study are available within the article, and its Supplementary Information file, and from the corresponding author upon reasonable request. The MS raw data have been deposited to the ProteomeXchange Consortium ( http://proteomecentral.proteomexchange.org) via the PRIDE partner repository with the data set identifier PXD004971.

## Additional information

**How to cite this article**: Manzi, L. *et al*. Carbene footprinting accurately maps binding sites in protein–ligand and protein–protein interactions. *Nat. Commun.* **7**, 13288 doi: 10.1038/ncomms13288 (2016).

**Publisher's note:** Springer Nature remains neutral with regard to jurisdictional claims in published maps and institutional affiliations.

## Supplementary Material

Supplementary Information Supplementary Figures, Supplementary Tables, Supplementary Methods, Supplementary References
