# Supplementary material for: Carbene footprinting accurately maps binding sites in protein–ligand and protein–protein interactions
Source: Nat Commun. 2016 Nov 16;7:13288. doi: 10.1038/ncomms13288 (PMC5116083; doi:10.1038/ncomms13288)
Supplement: Supplementary Information — Supplementary Figures, Supplementary Tables, Supplementary Methods, Supplementary References [file ncomms13288-s1.pdf]

27/07/2015 11:41:49

Acquisition Time (sec)	3.4210	Comment	UserID a_ba1 SampleID ab211data SupervisorID moses Lab Phone No. 13540 Slot Number 19				
Date	24 May 2013 16:49:20	Date Stamp	24 May 2013 16:49:20				
File Name	C:\Users\Andrew\Documents\Uni\Nottingham\NMR\Total NMR Backup\200-299\a_ba1.ab211-data\1\pdata\1\1r					Frequency (MHz)	400.13
Nucleus	1H	Number of Transients	16	Origin	av400	Original Points Count	16384
Owner	nmruser	Points Count	32768	Pulse Sequence	zg30	Receiver Gain	181.00
SW(cyclical) (Hz)	4789.27	Solvent	CHLOROFORM-d	Spectrum Offset (Hz)	2195.1875	Spectrum Type	STANDARD
Sweep Width (Hz)	4789.13	Temperature (degree C)	25.160				

a_ba1.ab211-data.001.001.1r.esp

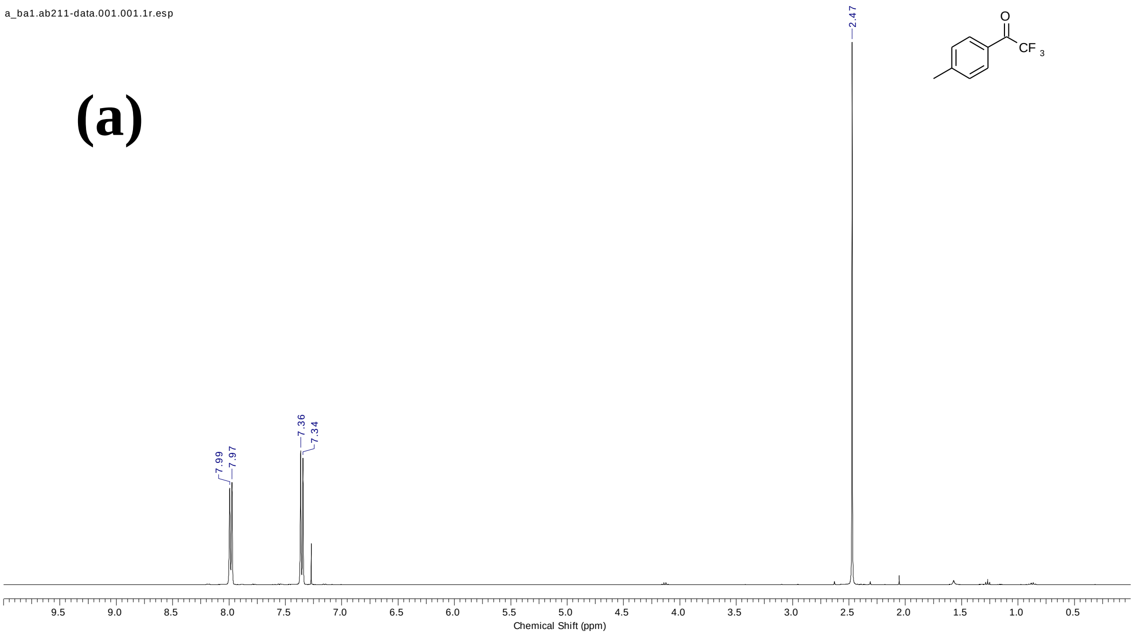

27/07/2015 11:55:46

Acquisition Time (sec)	0.6521	Comment	UserID a_ba1 SampleID ab211data SupervisorID moses Lab Phone No. 13540 Slot Number 19				
Date	25 May 2013 02:27:28	Date Stamp	25 May 2013 02:27:28				
File Name	C:\Users\Andrew\Documents\Uni\Nottingham\NMR\Total NMR Backup\200-299\a_ba1.ab211-data\4\pdata\1\1r					Frequency (MHz)	100.61
Nucleus	13C	Number of Transients	4096	Origin	av400	Original Points Count	16384
Owner	nmruser	Points Count	32768	Pulse Sequence	zgpg30	Receiver Gain	20642.50
SW(cyclical) (Hz)	25125.63	Solvent	CHLOROFORM-d	Spectrum Offset (Hz)	11064.6279	Spectrum Type	STANDARD
Sweep Width (Hz)	25124.86	Temperature (degree C)	25.160				

a_ba1.ab211-data.004.001.1r.esp

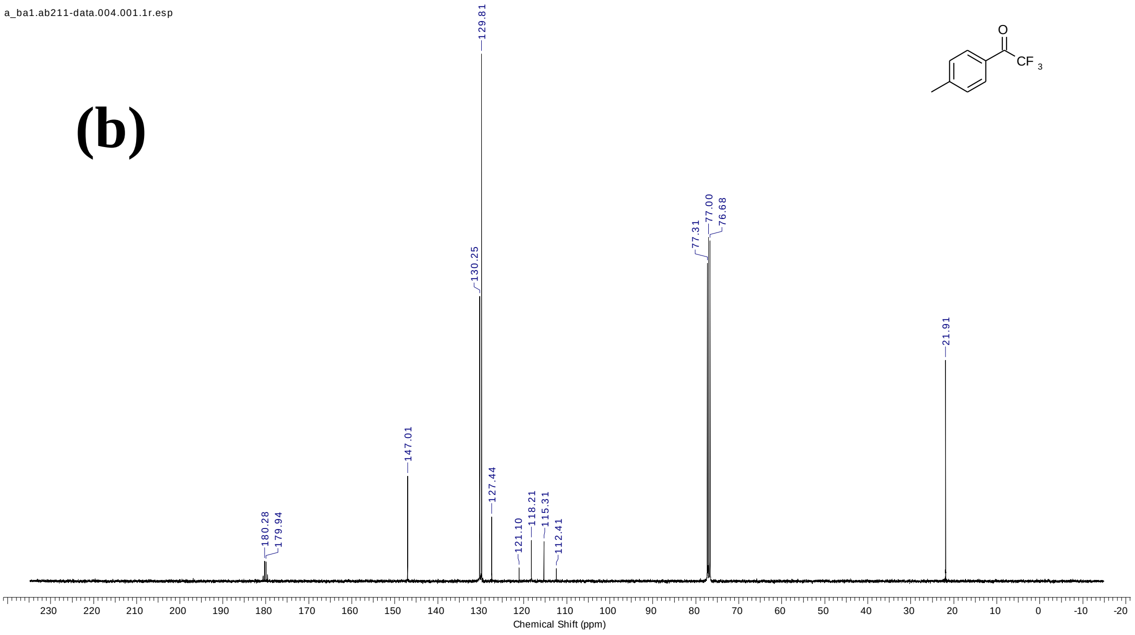

Supplemetary Figure 1. (**a**) ^{1}H and (**b**) ^{13}C NMR spectra of 2,2,2-trifluoro-1-(*p*-tolyl)ethanone **4**.

15/09/2015 08:49:21

Acquisition Time (sec)	3.9846	*Comment*	UserID a_ba1 SampleID ab840data SupervisorID moses Lab Phone No. 13540 Slot Number 24		
Date	14 Sep 2015 21:05:20			*Date Stamp*	14 Sep 2015 21:05:20
File Name	\\brukav3400hd\nmr_data\a_ba1\nmr\a_ba1.ab840data\1\pdata\1\1r			*Frequency (MHz)*	400.07
Nucleus	1H				
Number of Transients	16	*Origin*	spect	*Original Points Count*	32768
Owner	nmruser				
Points Count	65536	*Pulse Sequence*	zg30	*Receiver Gain*	38.97
SW(cyclical) (Hz)	8223.68				
Solvent	CHLOROFORM-d	*Spectrum Offset (Hz)*	2465.1133	*Spectrum Type*	STANDARD
Sweep Width (Hz)	8223.56				
Temperature (degree C)	25.008				

a_ba1.ab840data.001.001.1r.esp

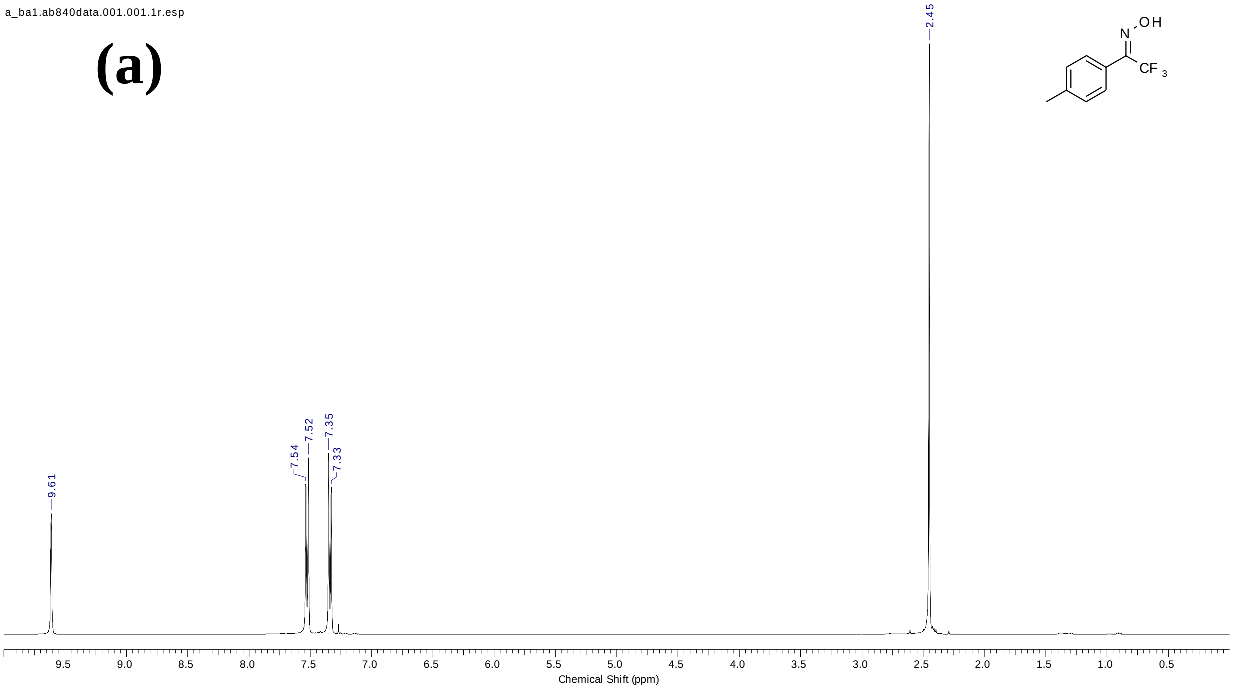

15/09/2015 08:54:25

Acquisition Time (sec)	0.6816	*Comment*	UserID a_ba1 SampleID ab840data SupervisorID moses Lab Phone No. 13540 Slot Number 24		
Date	14 Sep 2015 21:35:12			*Date Stamp*	14 Sep 2015 21:35:12
File Name	\\brukav3400hd\nmr_data\a_ba1\nmr\a_ba1.ab840data\2\pdata\1\1r			*Frequency (MHz)*	100.60
Nucleus	13C				
Number of Transients	1024	*Origin*	spect	*Original Points Count*	16384
Owner	nmruser				
Points Count	32768	*Pulse Sequence*	zgpg30	*Receiver Gain*	109.83
SW(cyclical) (Hz)	24038.46				
Solvent	CHLOROFORM-d	*Spectrum Offset (Hz)*	11060.0391	*Spectrum Type*	STANDARD
Sweep Width (Hz)	24037.73				
Temperature (degree C)	24.997				

a_ba1.ab840data.002.001.1r.esp

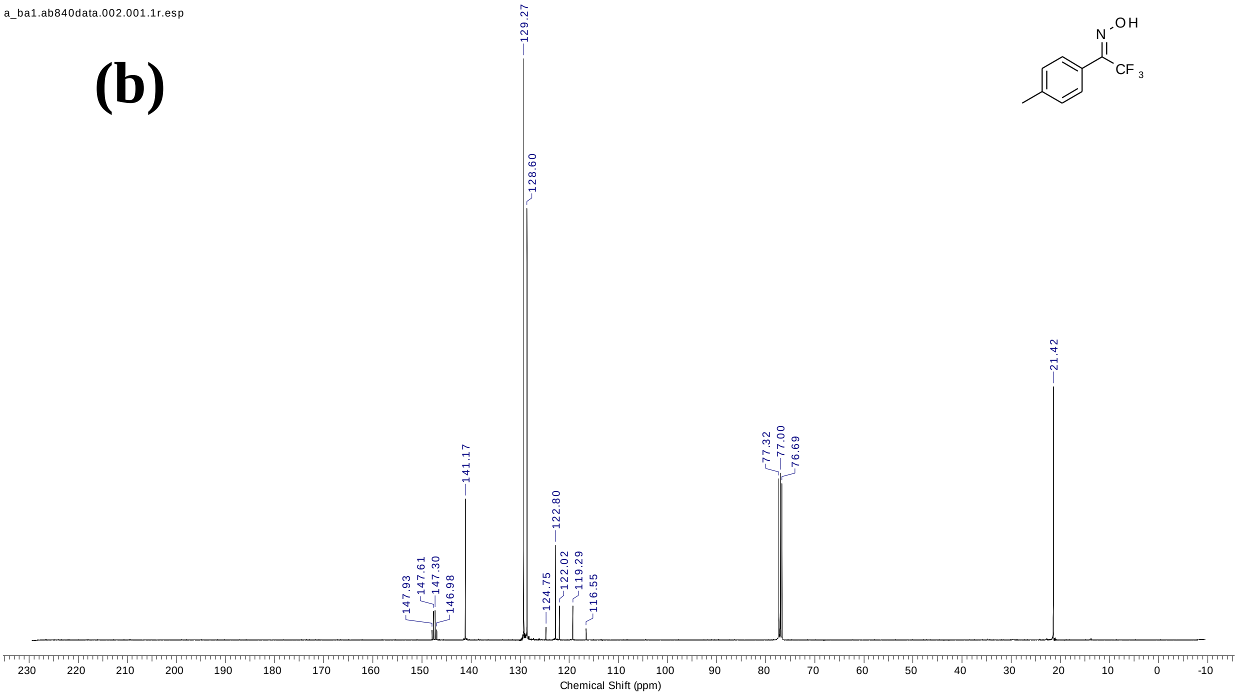

Supplementary Figure 2. (**a**) ^{1}H and (**b**) ^{13}C NMR spectra of 2,2,2-trifluoro-1-(*p*-tolyl)ethanone oxime **5**.

27/07/2015 11:20:44

Acquisition Time (sec)	3.4210	***Comment***	UserID a_ba1 SampleID ab186p SupervisorID moses Lab Phone No. 13540 Slot Number 33				
Date	17 May 2013 16:23:44			***Date Stamp***	17 May 2013 16:23:44		
File Name	C:\Users\Andrew\Documents\Uni\Nottingham\NMR\Total NMR Backup\100-199\a_ba1.ab186p\1\pdata\1\1r					***Frequency (MHz)***	400.13
Nucleus	1H	***Number of Transients***	16	***Origin***	av400	***Original Points Count***	16384
Owner	nmruser	***Points Count***	32768	***Pulse Sequence***	zg30	***Receiver Gain***	181.00
SW(cyclical) (Hz)	4789.27	***Solvent***	CHLOROFORM-d	***Spectrum Offset (Hz)***	2200.7131	***Spectrum Type***	STANDARD
Sweep Width (Hz)	4789.13	***Temperature (degree C)***	25.160				

a_ba1.ab186p.001.001.1r.esp

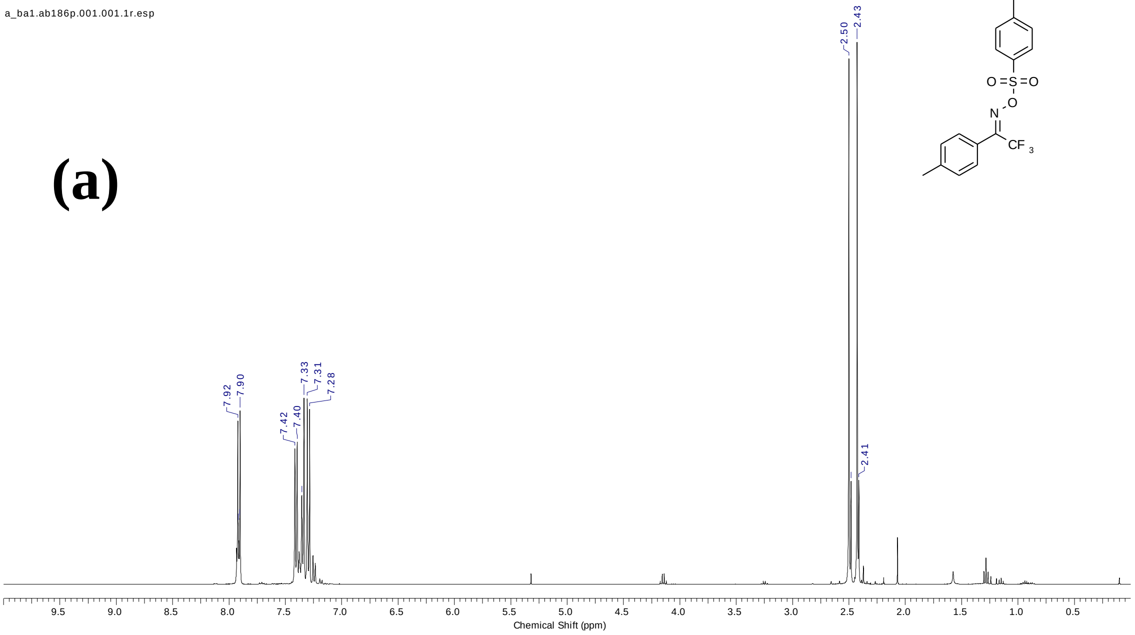

27/07/2015 11:22:37

Acquisition Time (sec)	0.6521	***Comment***	UserID a_ba1 SampleID ab186p SupervisorID moses Lab Phone No. 13540 Slot Number 33				
Date	17 May 2013 19:31:28	***Date Stamp***	17 May 2013 19:31:28				
File Name	C:\Users\Andrew\Documents\Uni\Nottingham\NMR\Total NMR Backup\100-199\a_ba1.ab186p\2\pdata\1\1r					***Frequency (MHz)***	100.61
Nucleus	13C	***Number of Transients***	4096	***Origin***	av400	***Original Points Count***	16384
Owner	nmruser	***Points Count***	32768	***Pulse Sequence***	zgpg30	***Receiver Gain***	18390.40
SW(cyclical) (Hz)	25125.63	***Solvent***	CHLOROFORM-d	***Spectrum Offset (Hz)***	11063.0947	***Spectrum Type***	STANDARD
Sweep Width (Hz)	25124.86	***Temperature (degree C)***	25.160				

a_ba1.ab186p.002.001.1r.esp

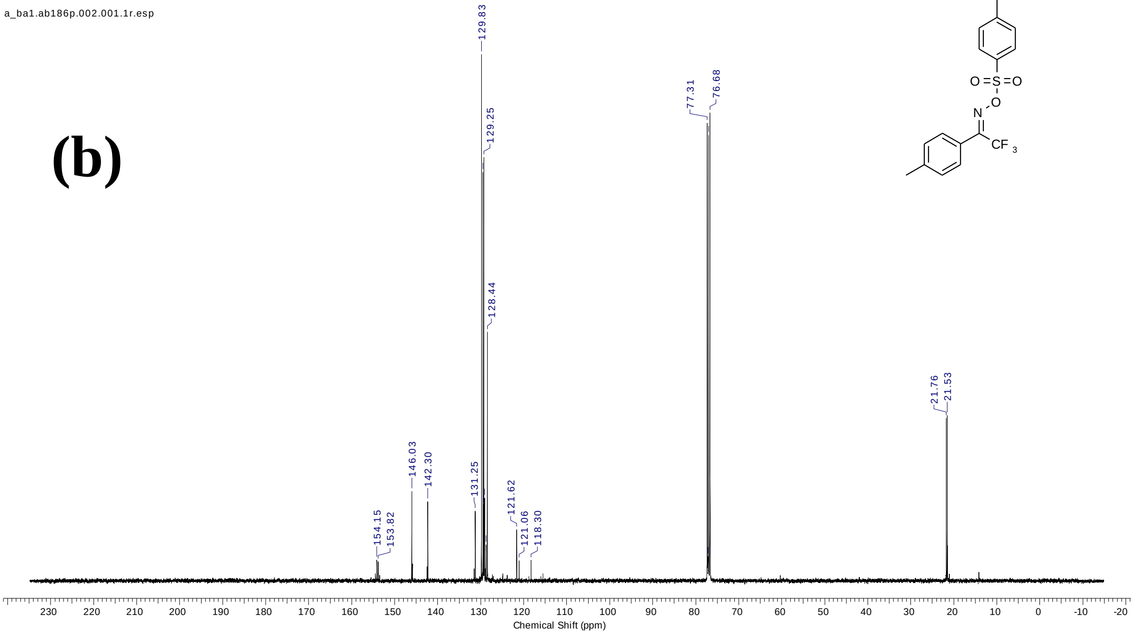

Supplementary Figure 3. (**a**) ^{1}H and (**b**) ^{13}C NMR spectra of 2,2,2-trifluoro-1-(*p*-tolyl)ethanone *O*-tosyl oxime **6**.

27/07/2015 11:13:06

Acquisition Time (sec)	5.3084	Comment	Slot No. 51 Sample ID ab162p SupervisorID moses Lab Phone No. 13540 UserID a_ba1				
Date	27 Mar 2013 19:03:28			Date Stamp	27 Mar 2013 19:03:28		
File Name	C:\Users\Andrew\Documents\Uni\Nottingham\NMR\Total NMR Backup\100-199\a_ba1.ab162p\1\pdata\1\1r					Frequency (MHz)	300.13
Nucleus	1H	Number of Transients	64	Origin	dpx300	Original Points Count	32768
Owner	nmruser	Points Count	65536	Pulse Sequence	zg30	Receiver Gain	406.40
SW(cyclical) (Hz)	6172.84	Solvent	CHLOROFORM-d	Spectrum Offset (Hz)	1850.1608	Spectrum Type	STANDARD
Sweep Width (Hz)	6172.75	Temperature (degree C)	25.000				

a_ba1.ab162p.001.001.1r.esp

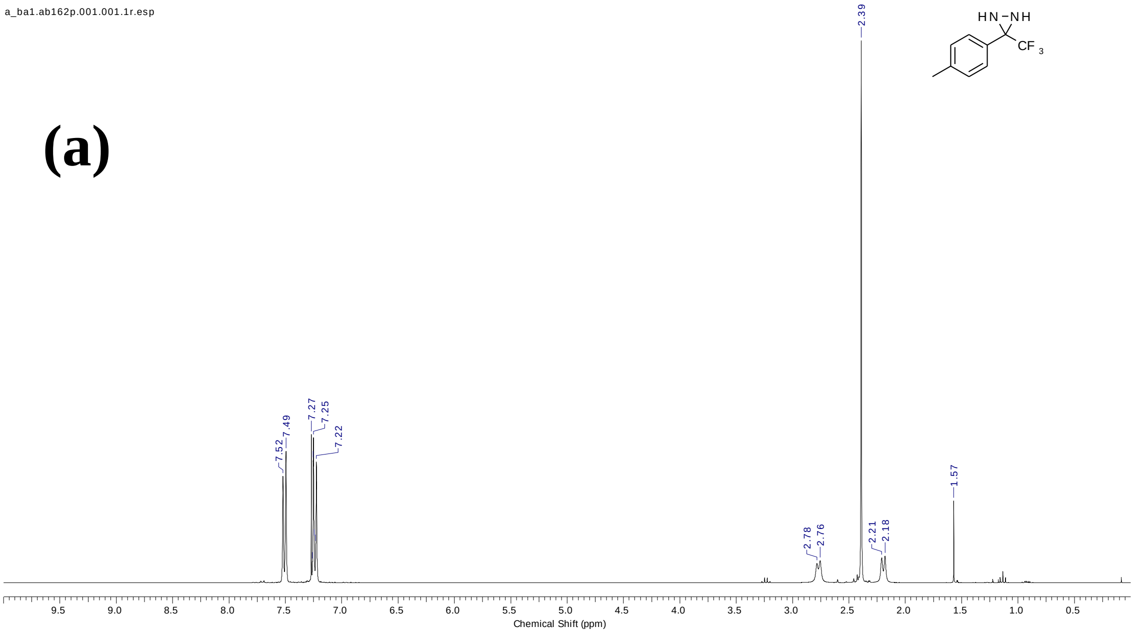

27/07/2015 11:17:43

Acquisition Time (sec)	0.8700	Comment	Slot No. 51 Sample ID ab162p SupervisorID moses Lab Phone No. 13540 UserID a_ba1				
Date	28 Mar 2013 23:51:28	Date Stamp	28 Mar 2013 23:51:28				
File Name	C:\Users\Andrew\Documents\Uni\Nottingham\NMR\Total NMR Backup\100-199\a_ba1.ab162p\3\pdata\1\1r					Frequency (MHz)	75.47
Nucleus	13C	Number of Transients	4096	Origin	dpx300	Original Points Count	16384
Owner	nmruser	Points Count	32768	Pulse Sequence	zgpg30	Receiver Gain	10321.30
SW(cyclical) (Hz)	18832.39	Solvent	CHLOROFORM-d	Spectrum Offset (Hz)	8300.4121	Spectrum Type	STANDARD
Sweep Width (Hz)	18831.82	Temperature (degree C)	25.000				

a_ba1.ab162p.003.001.1r.esp

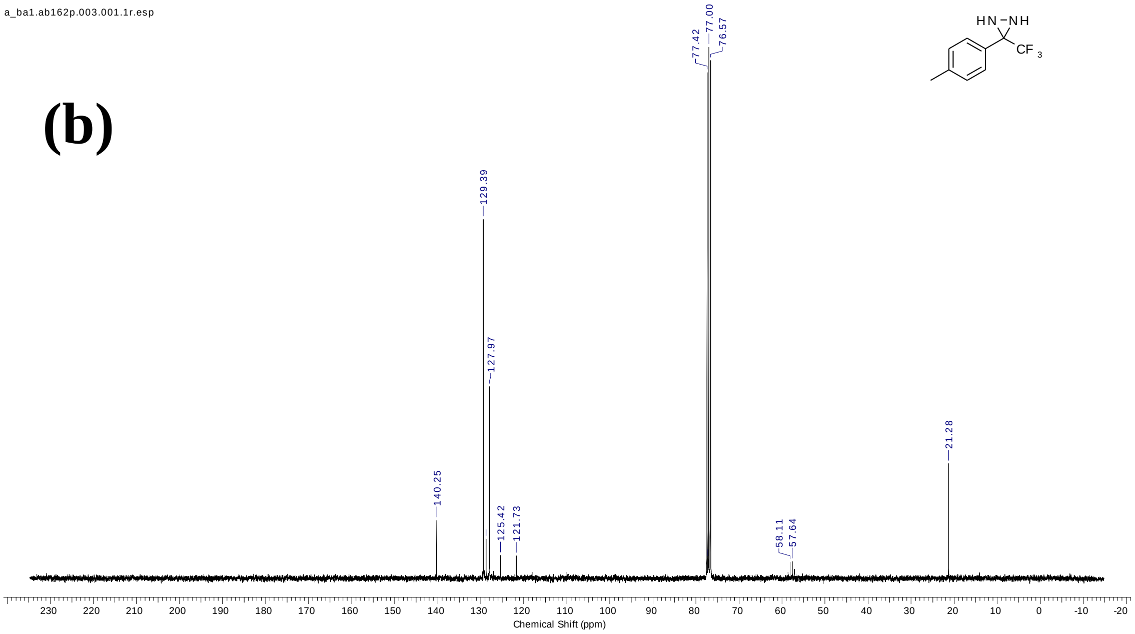

Supplementary Figure 4. (**a**) ^{1}H and (**b**) ^{13}C NMR spectra of 3-(*p*-tolyl)-3-(trifluoromethyl)diaziridine **7**.

27/07/2015 11:29:48

Parameter	Value	Parameter	Value	Parameter	Value
Acquisition Time (sec)	3.4210	***Comment***	UserID a_ba1 SampleID ab189pure SupervisorID moses Lab Phone No. 13540 Slot Number 41		
Date	09 May 2013 06:28:32	***Date Stamp***	09 May 2013 06:28:32		
File Name	C:\Users\Andrew\Documents\Uni\Nottingham\NMR\Total NMR Backup\100-199\a_ba1.ab189pure\1\pdata\1\1r			***Frequency (MHz)***	400.13
Nucleus	1H	***Number of Transients***	16	***Origin***	av400
Original Points Count	16384	***Owner***	nmruser	***Points Count***	32768
Pulse Sequence	zg30	***Receiver Gain***	181.00	***SW(cyclical) (Hz)***	4789.27
Solvent	DMSO-d6	***Spectrum Offset (Hz)***	2197.4238	***Spectrum Type***	STANDARD
Sweep Width (Hz)	4789.13	***Temperature (degree C)***	25.160		

a_ba1.ab189pure.001.001.1r.esp

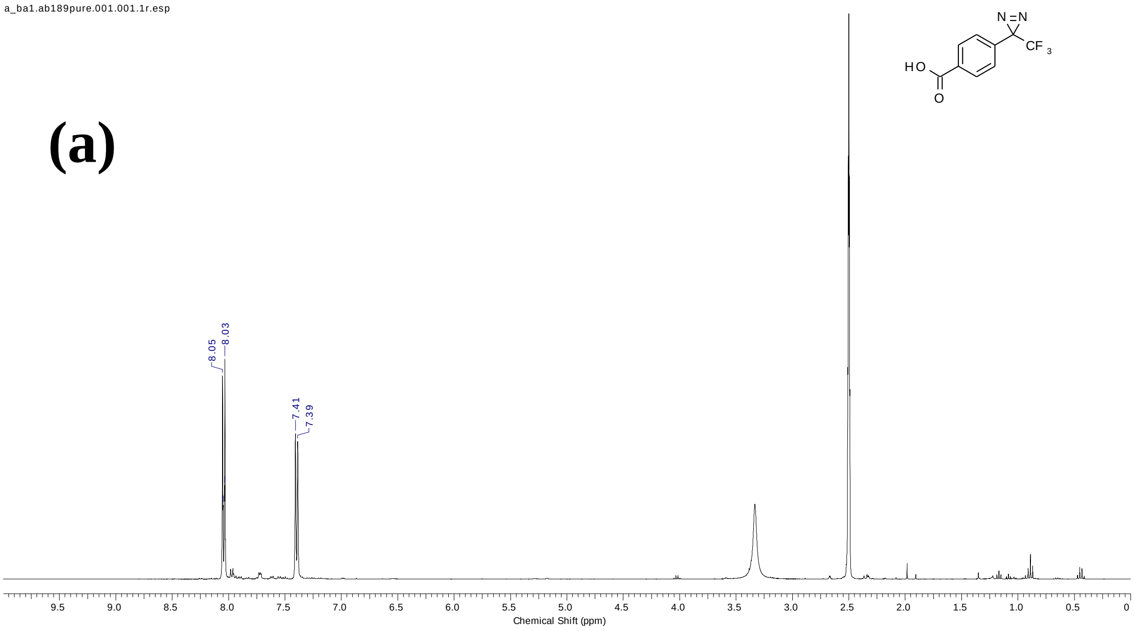

27/07/2015 11:31:22

Parameter	Value	Parameter	Value	Parameter	Value
Acquisition Time (sec)	0.6521	***Comment***	UserID a_ba1 SampleID ab189pure SupervisorID moses Lab Phone No. 13540 Slot Number 41		
Date	09 May 2013 19:37:52	***Date Stamp***	09 May 2013 19:37:52		
File Name	C:\Users\Andrew\Documents\Uni\Nottingham\NMR\Total NMR Backup\100-199\a_ba1.ab189pure\2\pdata\1\1r			***Frequency (MHz)***	100.61
Nucleus	13C	***Number of Transients***	4096	***Origin***	av400
Original Points Count	16384	***Owner***	nmruser	***Points Count***	32768
Pulse Sequence	zgpg30	***Receiver Gain***	18390.40	***SW(cyclical) (Hz)***	25125.63
Solvent	DMSO-d6	***Spectrum Offset (Hz)***	11019.1729	***Spectrum Type***	STANDARD
Sweep Width (Hz)	25124.86	***Temperature (degree C)***	25.160		

a_ba1.ab189pure.002.001.1r.esp

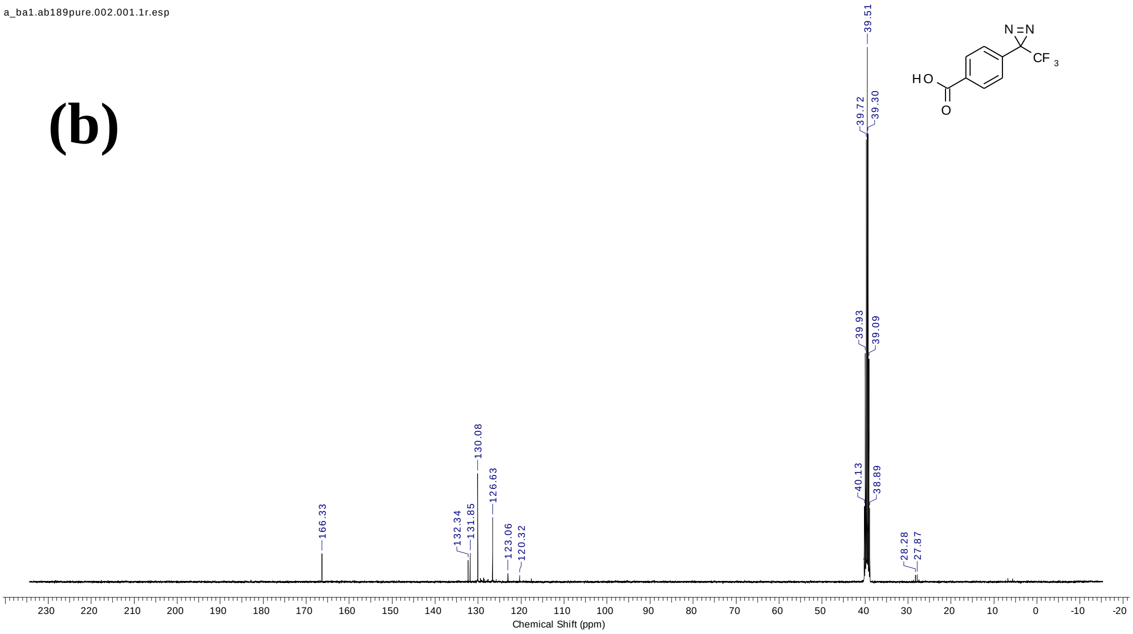

Supplementary Figure 5. (**a**) ^{1}H and (**b**) ^{13}C NMR spectra of 4-(3-(trifluoromethyl)-3*H*-diazirin-3-yl)benzoic acid **8**.

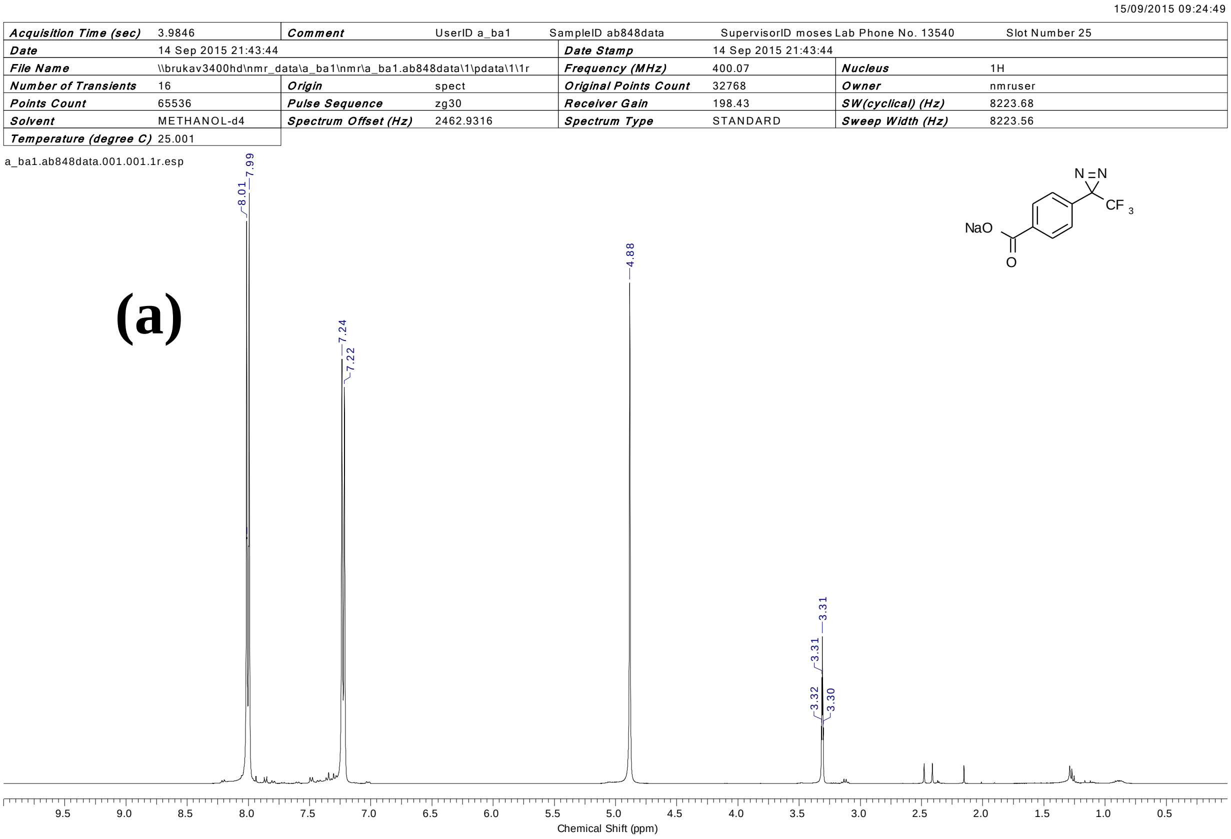

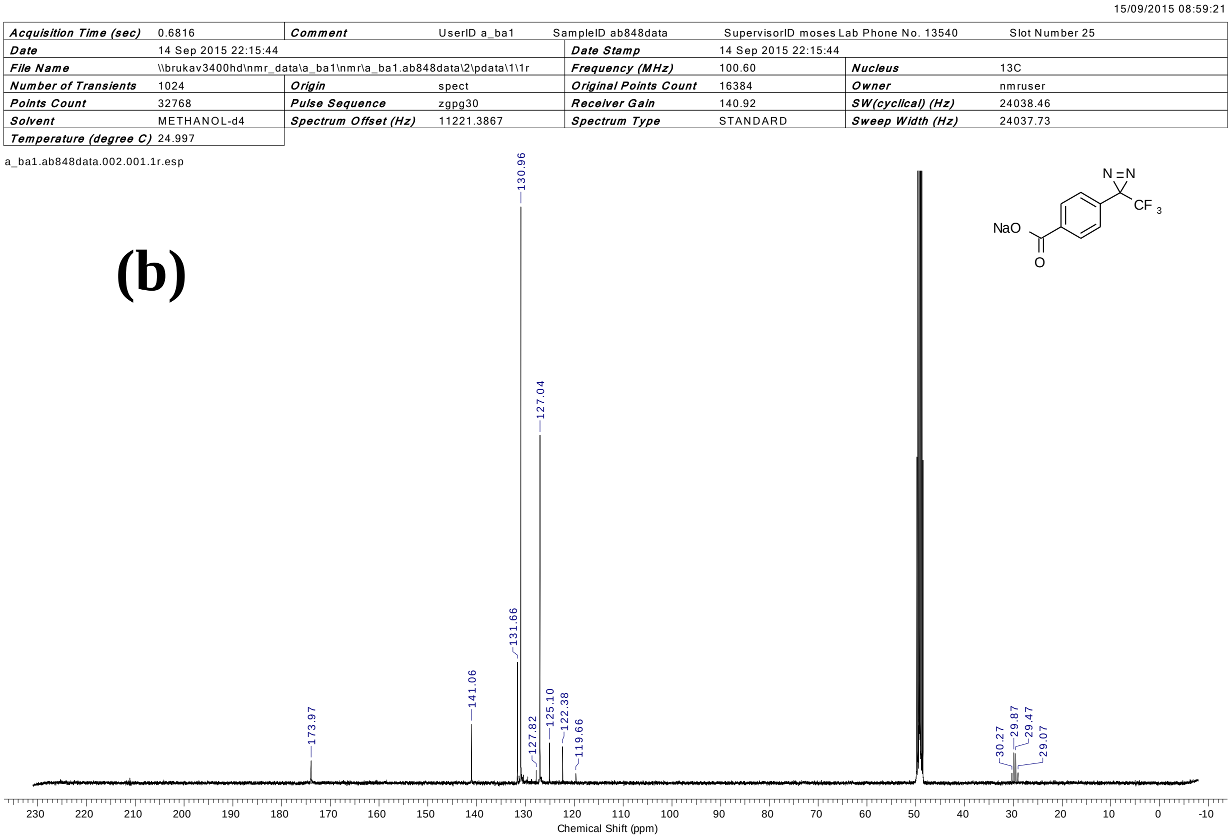

Supplementary Figure 6. (**a**) ^{1}H and (**b**) ^{13}C NMR spectra of Sodium 4-(3-(trifluoromethyl)-3*H*-diazirin-3-yl)benzoate **2**.

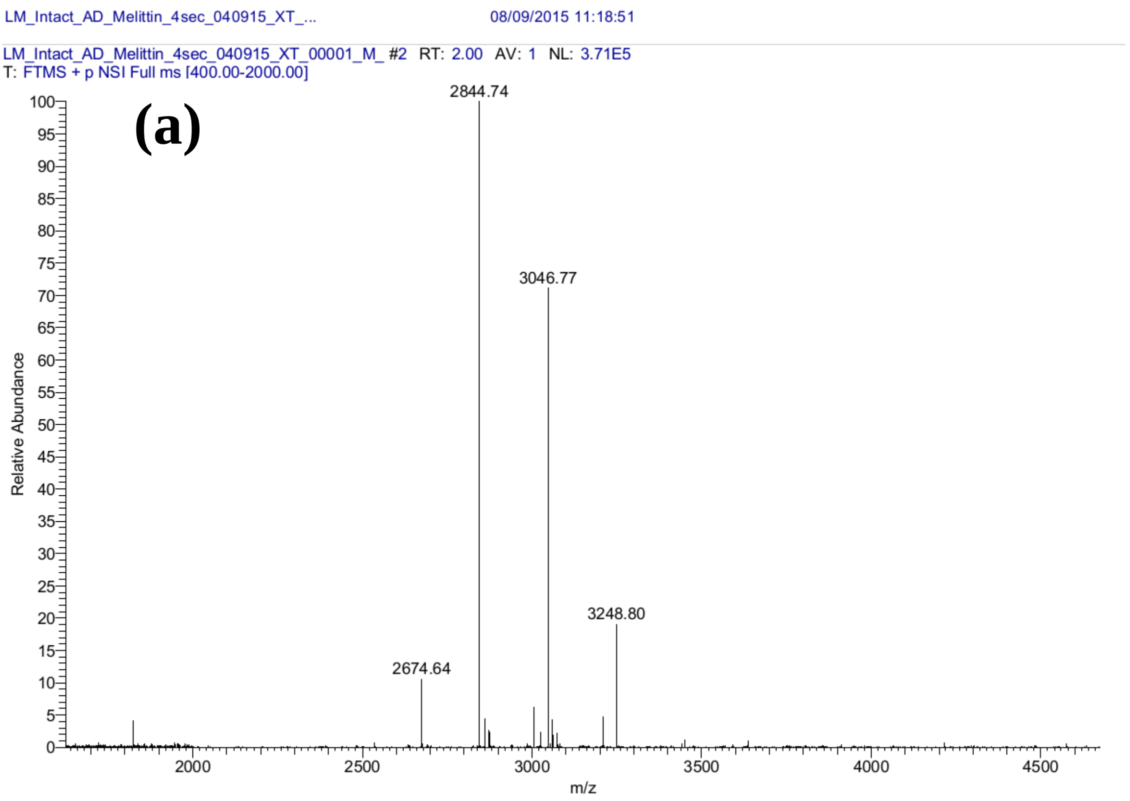

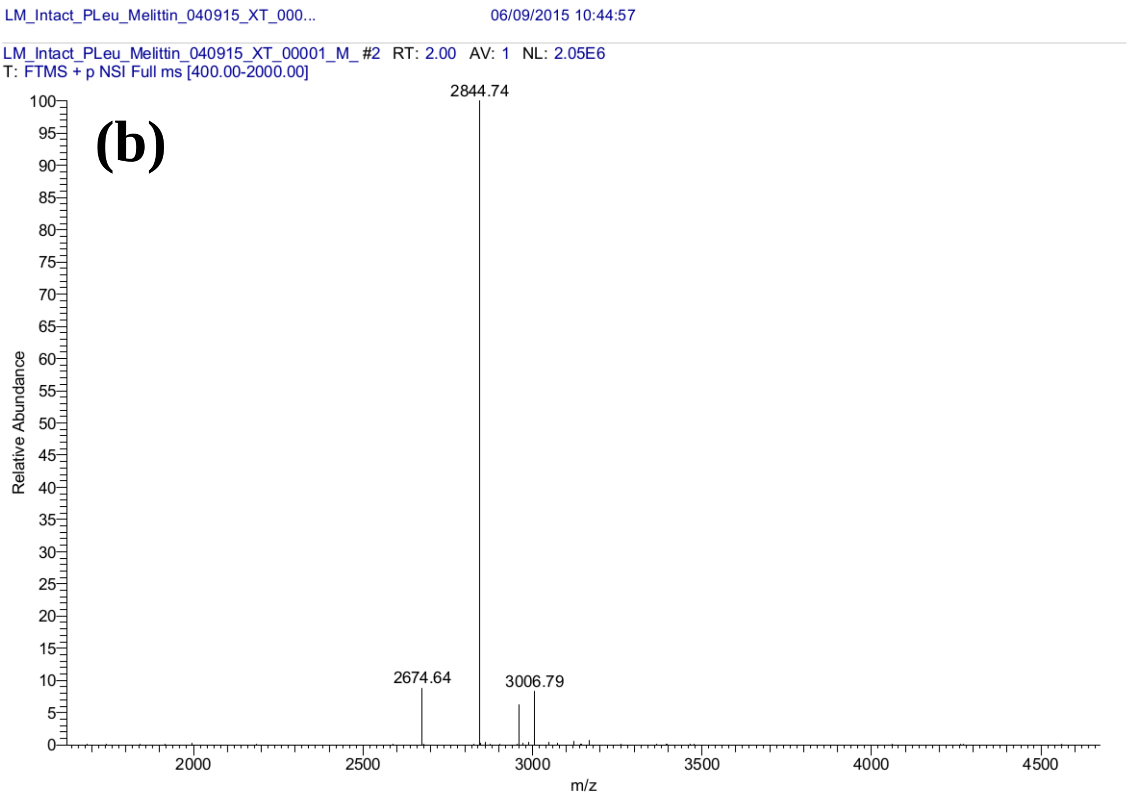

Supplementary Figure 7. Deconvoluted ESI-MS spectrum of melittin (M_r 2844.7 (monoisotopic)) labelled with (**a**) aryldiazirine **2**, and (**b**) photoleucine **1**.

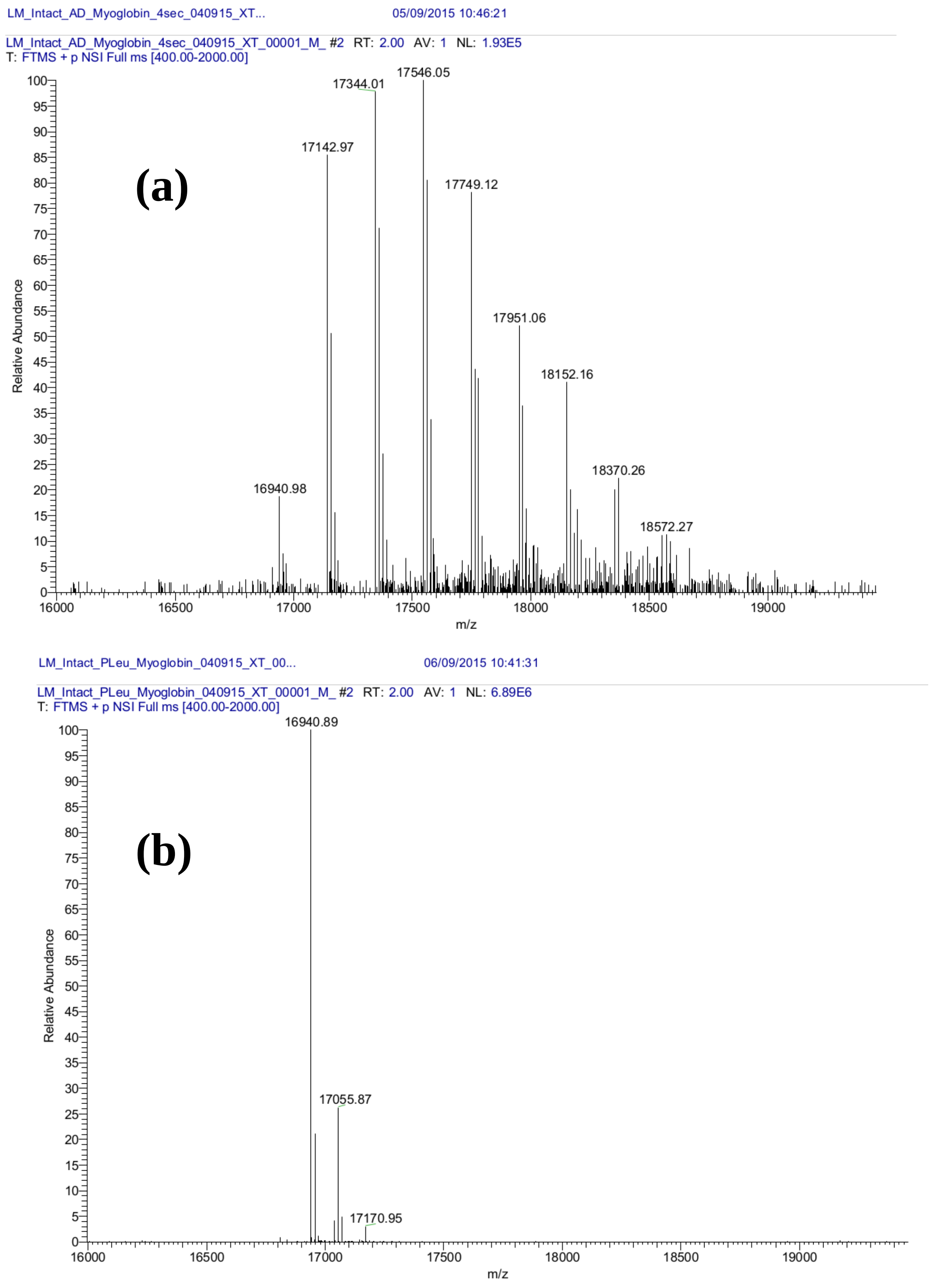

Supplementary Figure 8. Deconvoluted ESI-MS spectrum of myoglobin (M_r 16940.9 (monoisotopic) labelled with (**a**) aryldiazirine **2**, and (**b**) photoleucine **1**.

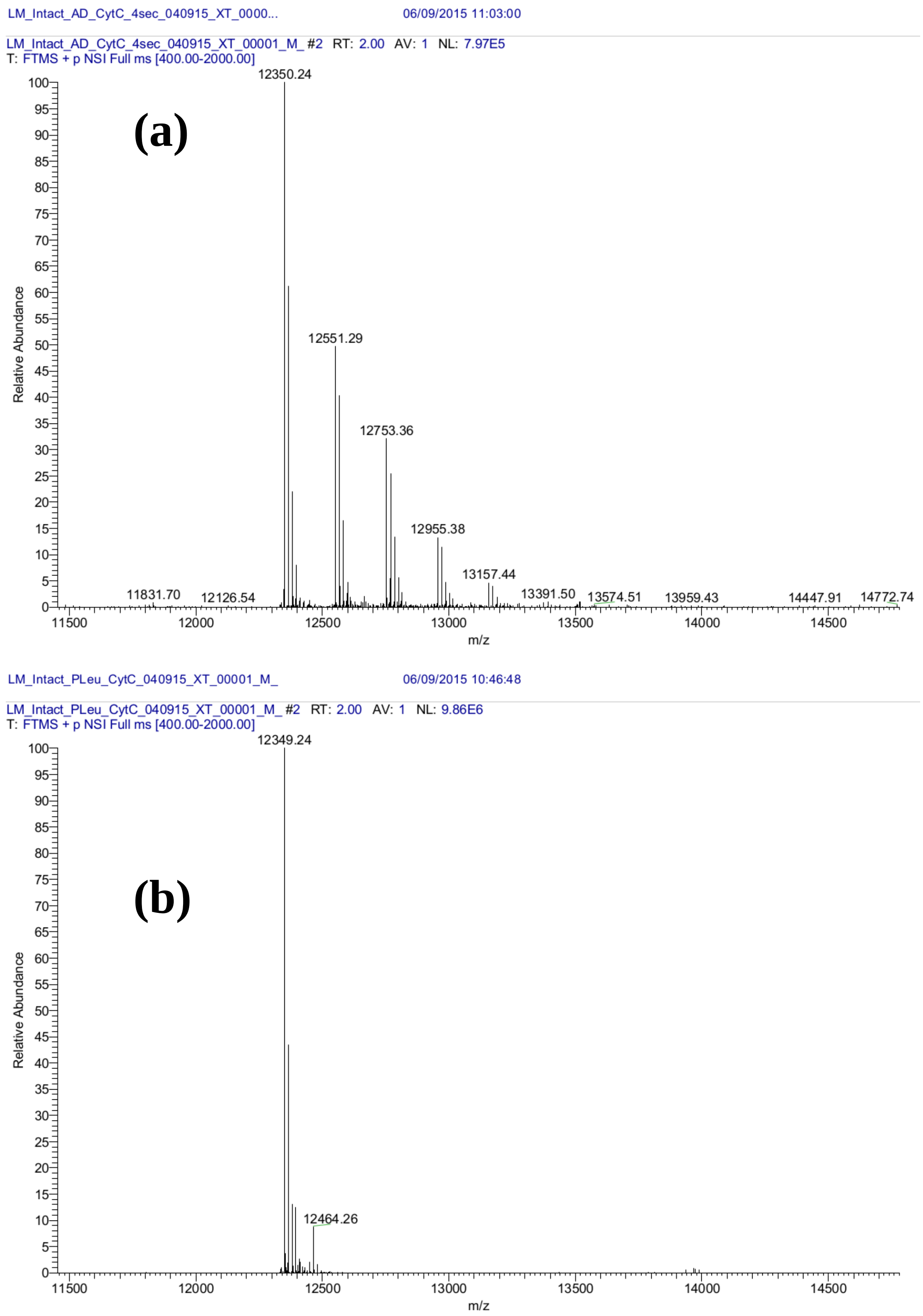

Supplementary Figure 9. Deconvoluted ESI-MS spectrum of cytochrome C (M_r 12350.2 (monoisotopic)) labelled with (**a**) aryldiazirine **2**, and (**b**) photoleucine **1**.

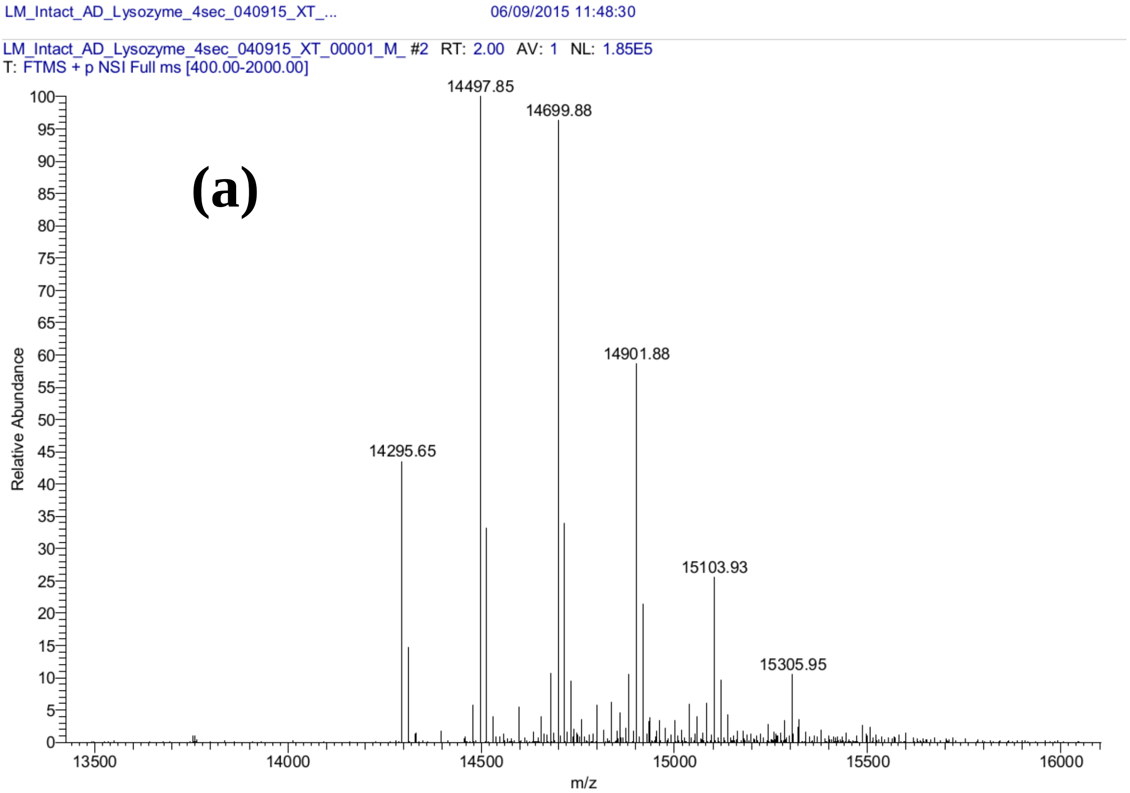

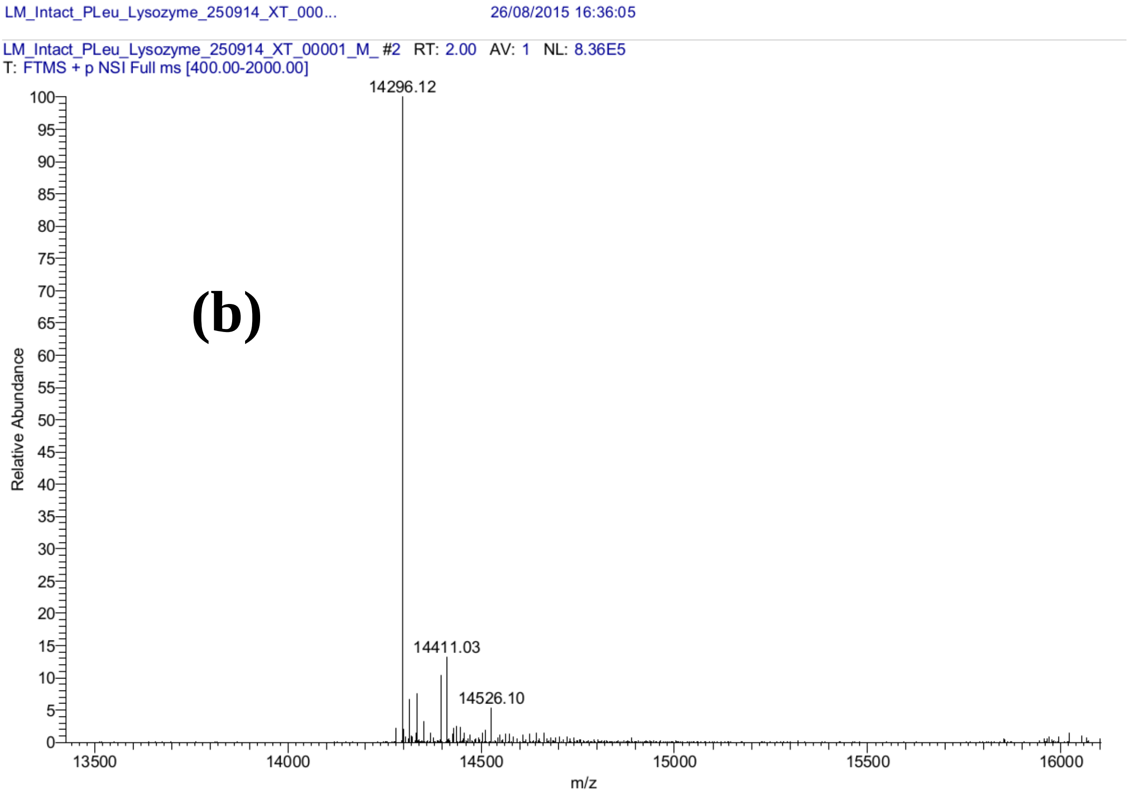

Supplementary Figure 10. Deconvoluted ESI-MS spectrum of HEWL (M_r 14295.8 (monoisotopic)) labelled with (**a**) aryldiazirine **2**, and (**b**) photoleucine **1**.

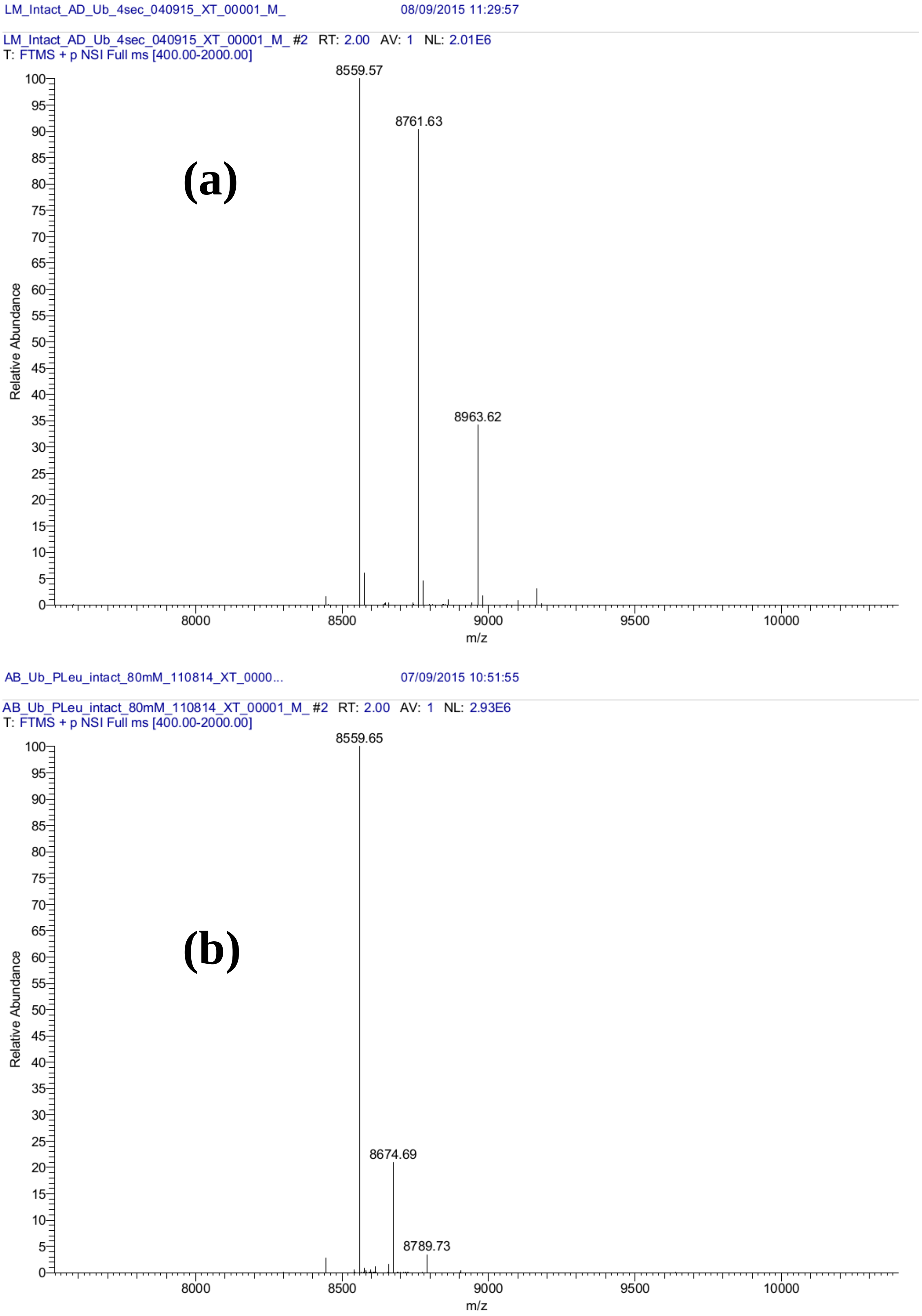

Supplementary Figure 11. Deconvoluted ESI-MS spectrum of ubiquitin (M_r 8559.6 (monoisotopic)) labelled with (**a**) aryldiazirine **2**, and (**b**) photoleucine **1**.

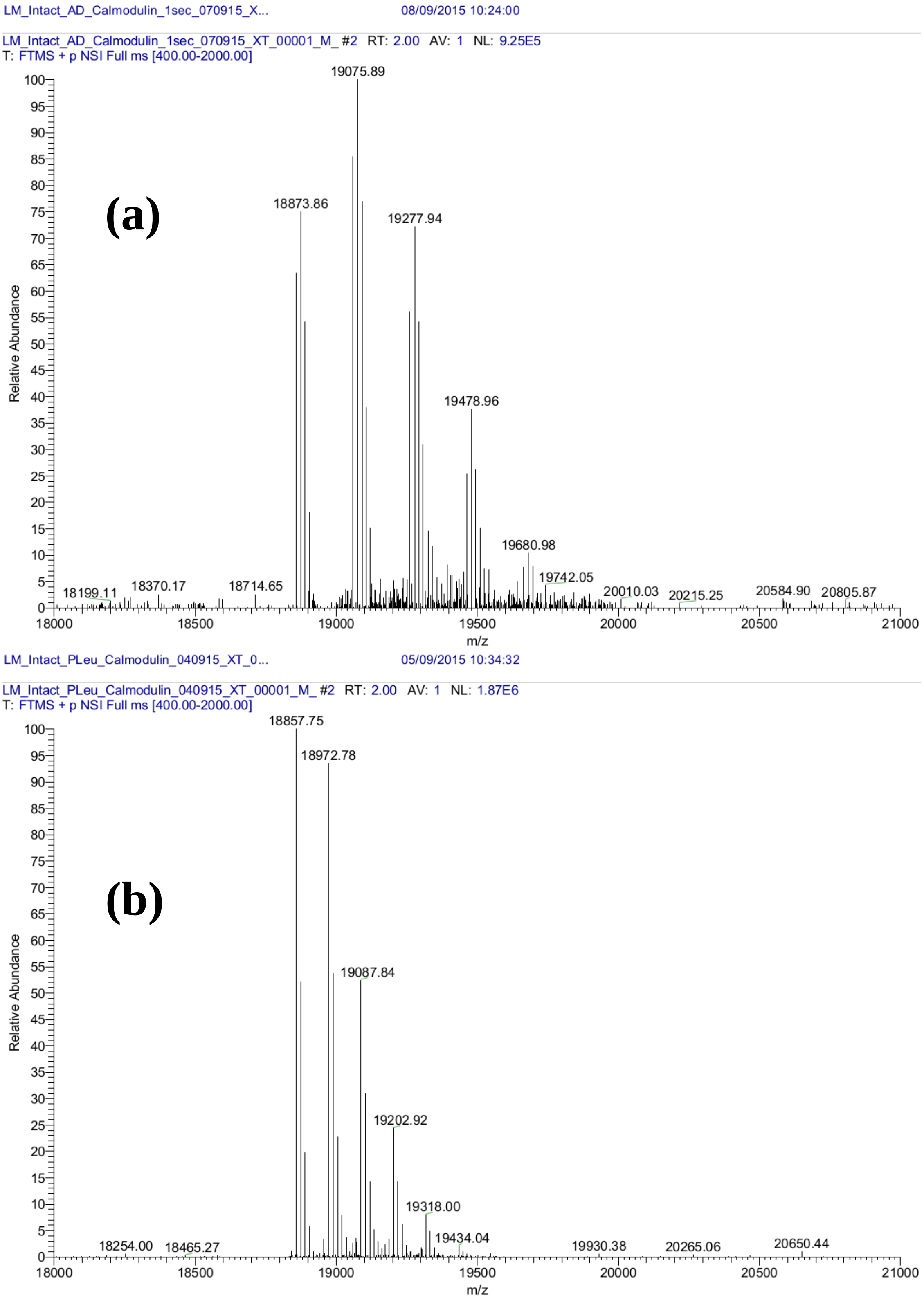

Supplementary Figure 12. Deconvoluted ESI-MS spectrum of calmodulin (M_r 18857.8 (monoisotopic)) labelled with (**a**) aryldiazirine **2**, and (**b**) photoleucine **1**.

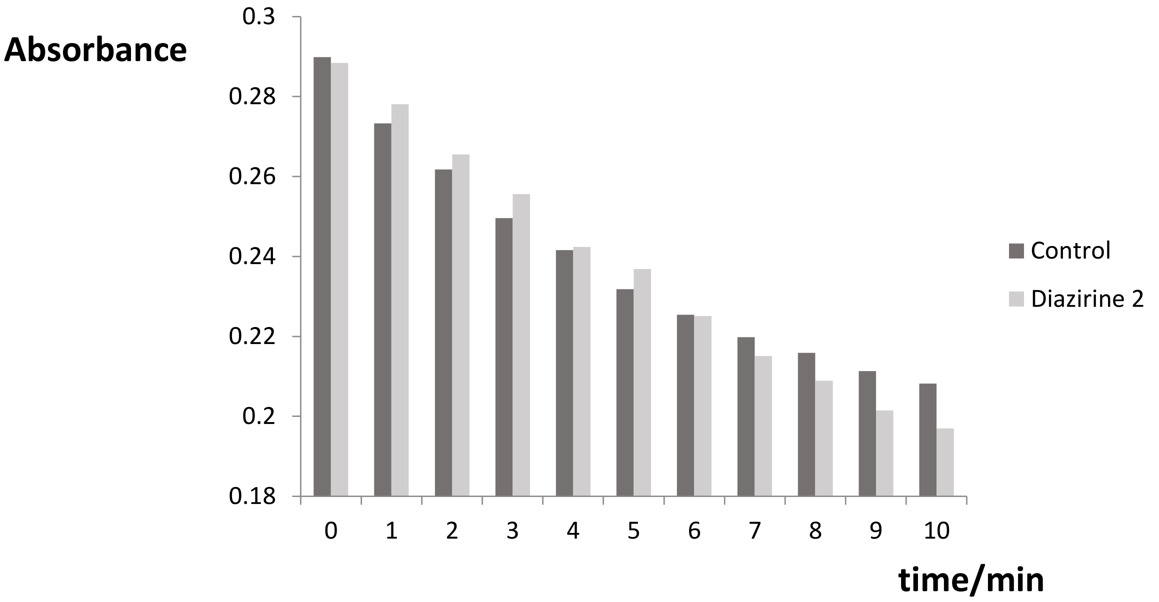

Supplementary Figure 13 - HEWL activity assay. Plot of absorbance vs. time for lysozyme activity using *Micrococcus lysodeikticus* cells (see Methods for details) showing that the presence of diazirine **2** has no effect on the activity of the enzyme, and by inference its structure. Control (+ lysozyme, - **2**), diazirine 2 (+ lysozyme, + **2**).

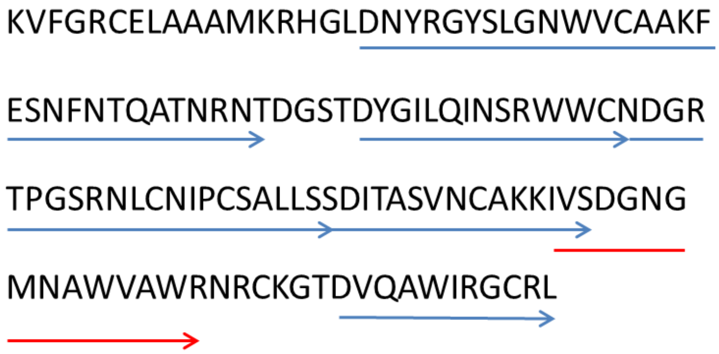

Supplementary Figure 14 - HEWL coverage. Sequence coverage obtained for carbene labeled lysozyme using AspN (blue arrows) and Trypsin (red arrow).

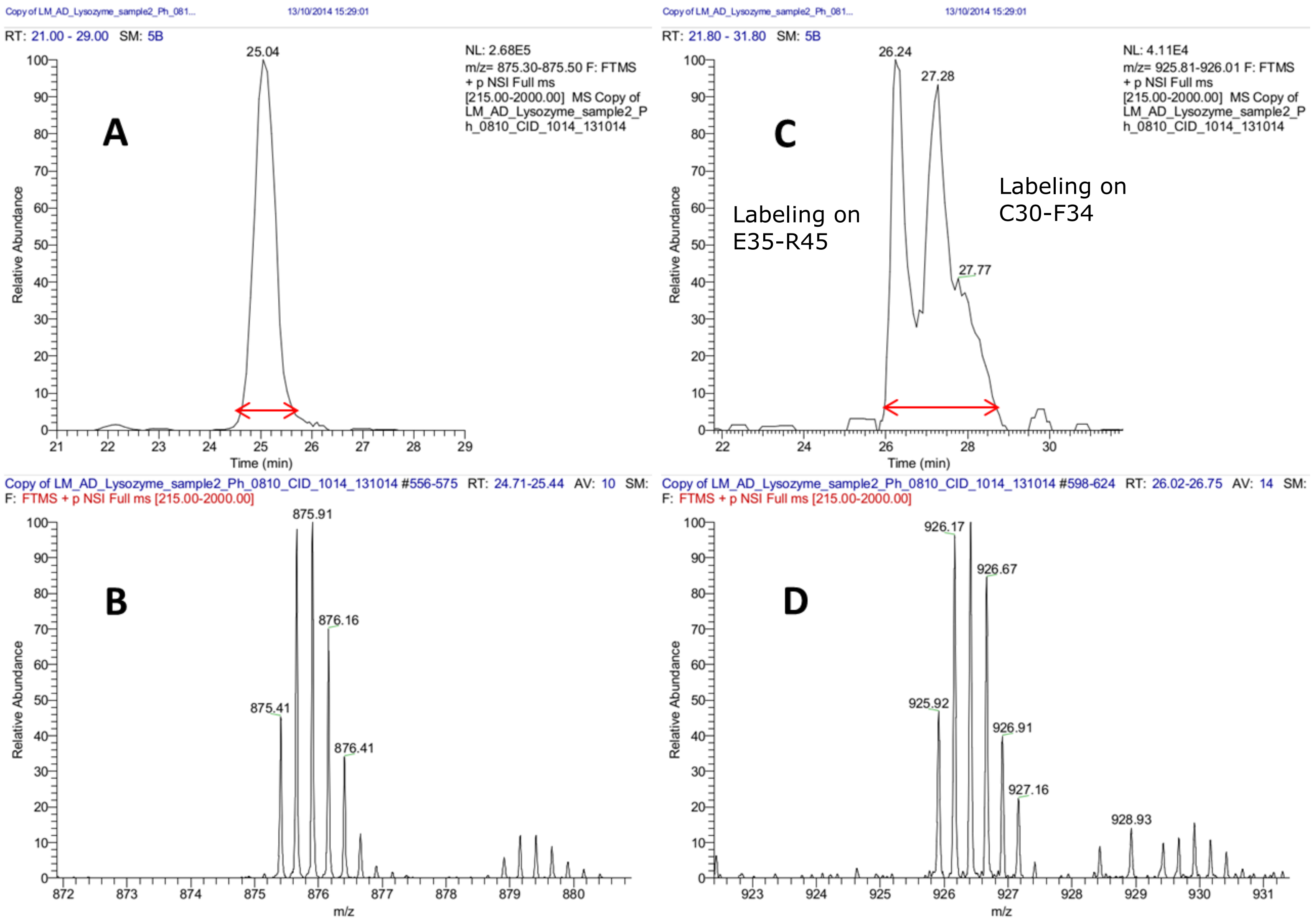

Supplementary Figure 15 – HEWL Peptide D18-T47 XIC and Spectrum: extracted ion chromatograms (XIC) for the unlabeled and labeled precursors (A and C, respectively, mass tolerance *m/z* 0.1), and spectra of the unlabeled and labeled precursor ions (B and D, respectively). The red arrow indicates approximately the area over which the data has been averaged.

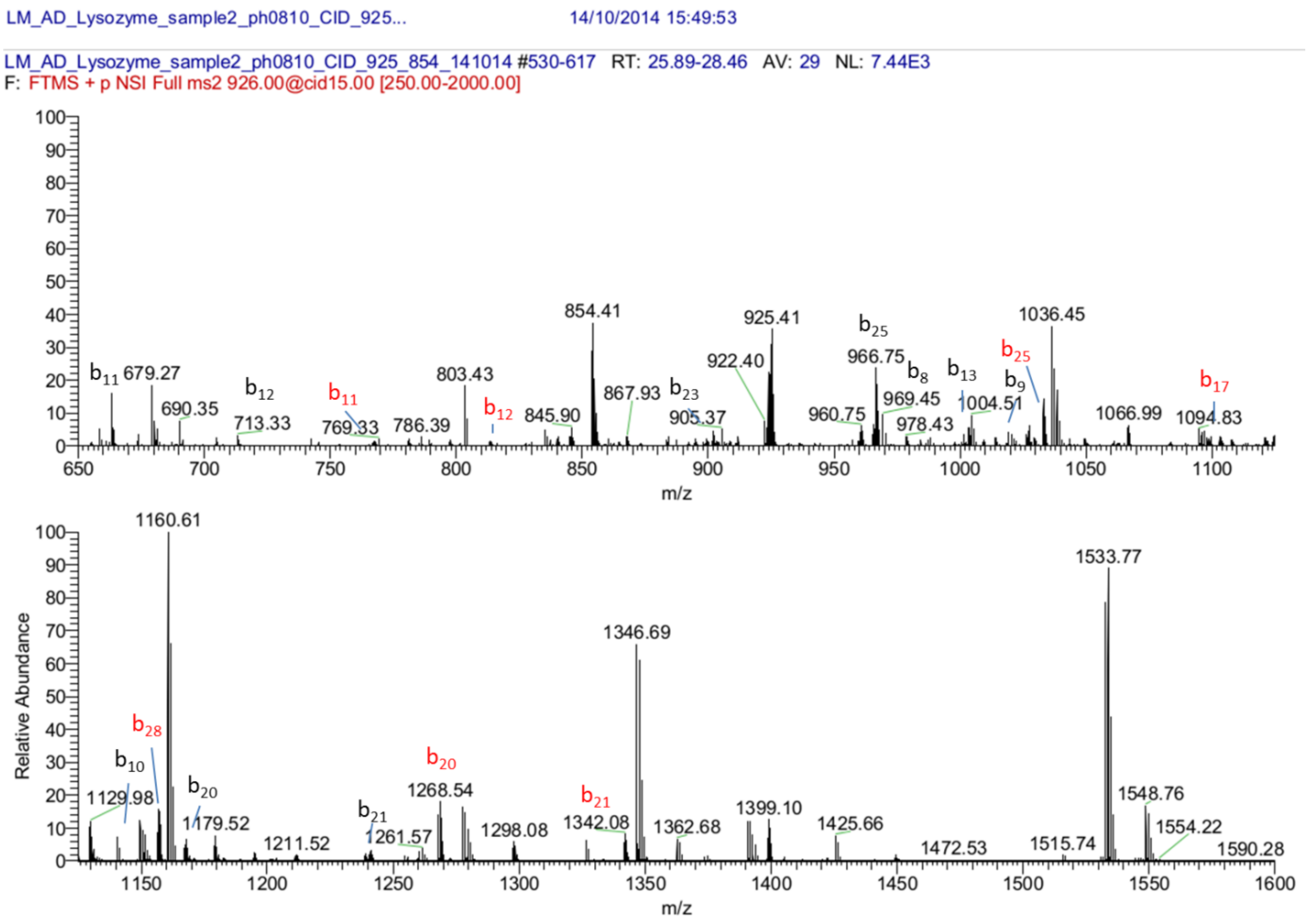

Supplementary Figure 16 – HEWL Labelled Peptide D18-T47 CID Spectrum: example of MS/MS data. Signals labelled in black and red correspond respectively to unlabelled and labelled ions.

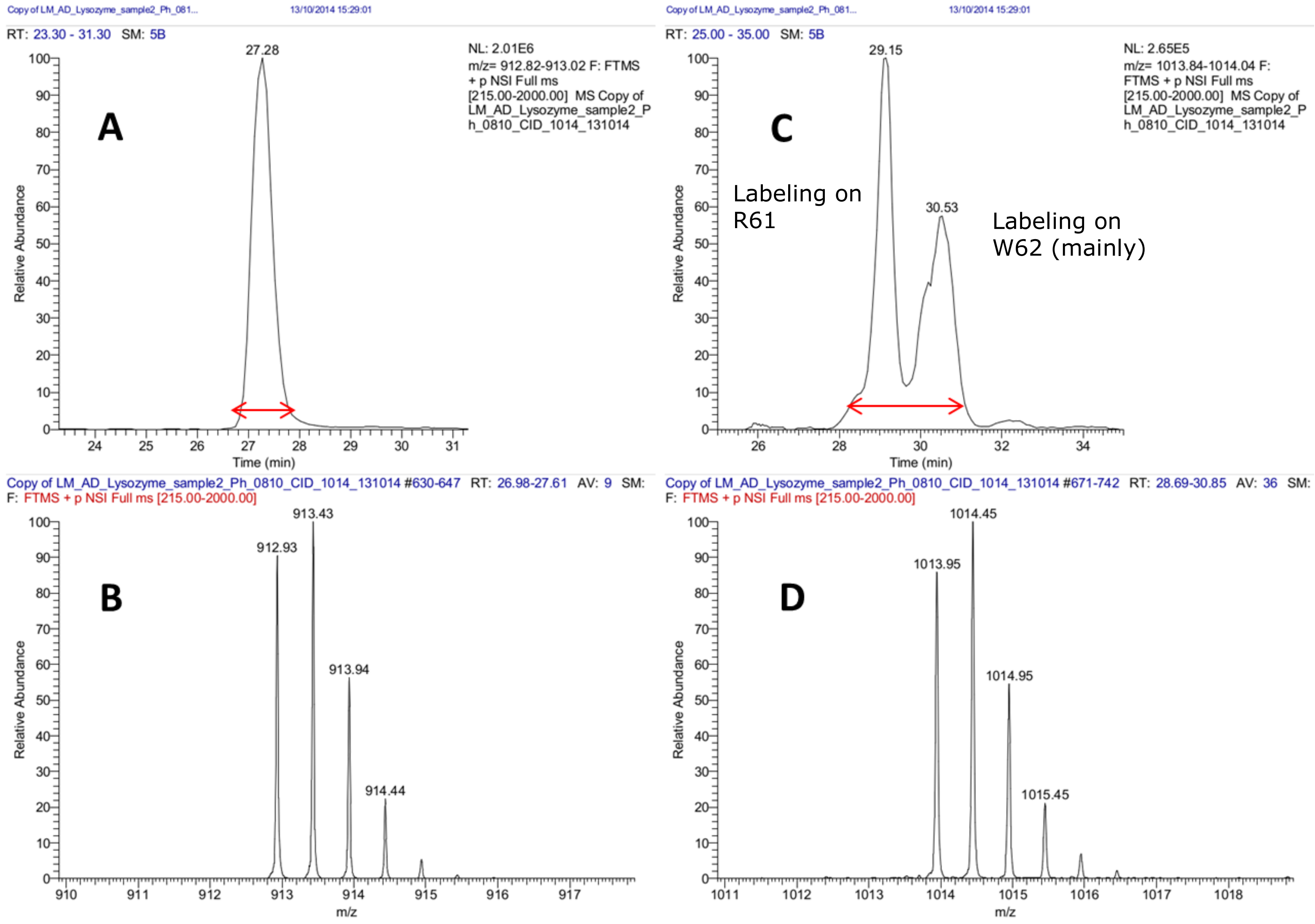

Supplementary Figure 17 – HEWL Peptide D52-N65 XIC and Spectrum: extracted ion chromatograms (XIC) for the unlabeled and labeled precursors (A and C, respectively, mass tolerance *m/z* 0.1), and spectra of the unlabeled and labeled precursor ions (B and D, respectively). The red arrow indicates approximately the area over which the data has been averaged.

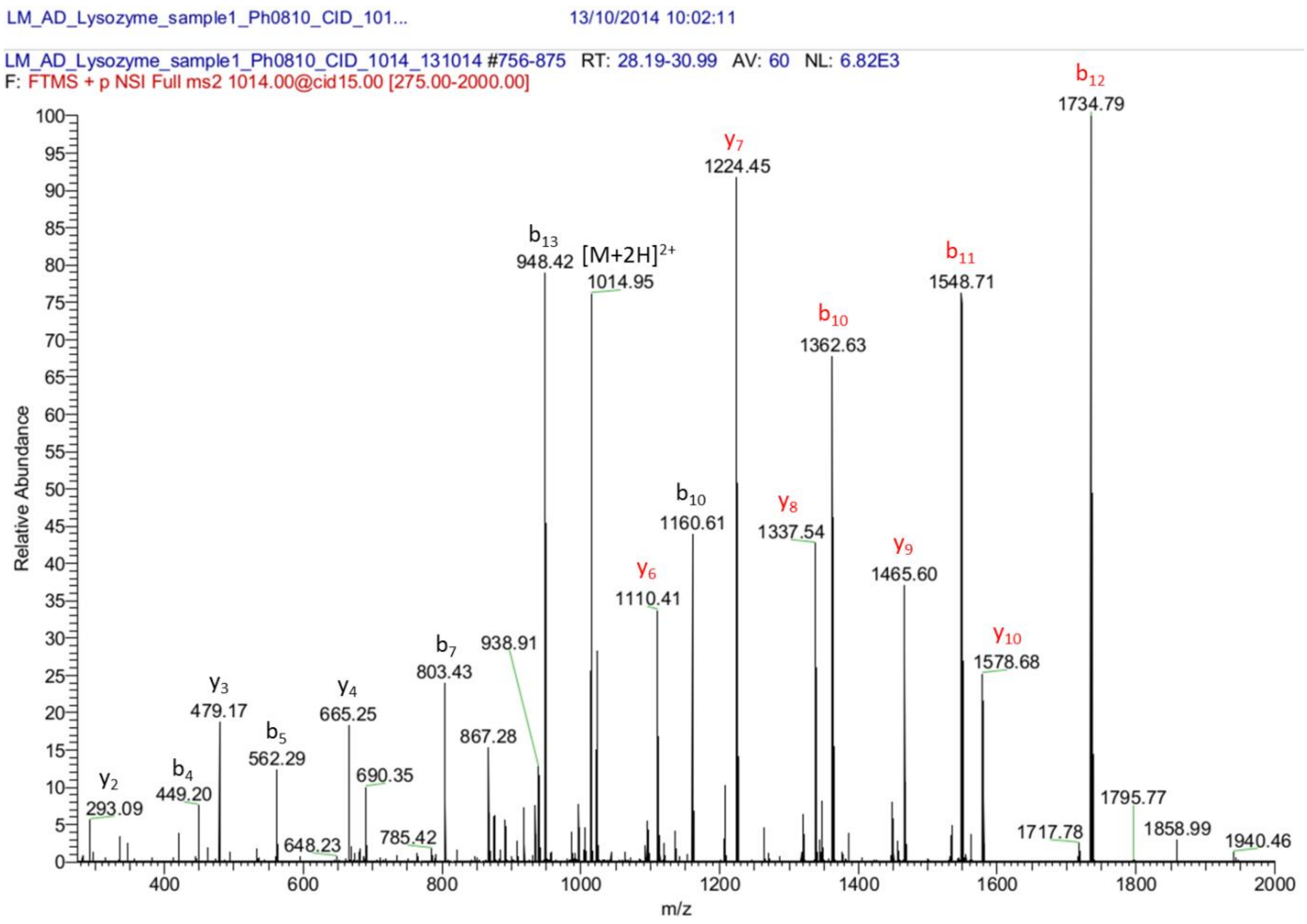

Supplementary Figure 18 – HEWL Labelled Peptide D52-N65 CID Spectrum: example of MS/MS data. Signals labelled in black and red correspond respectively to unlabelled and labelled ions.

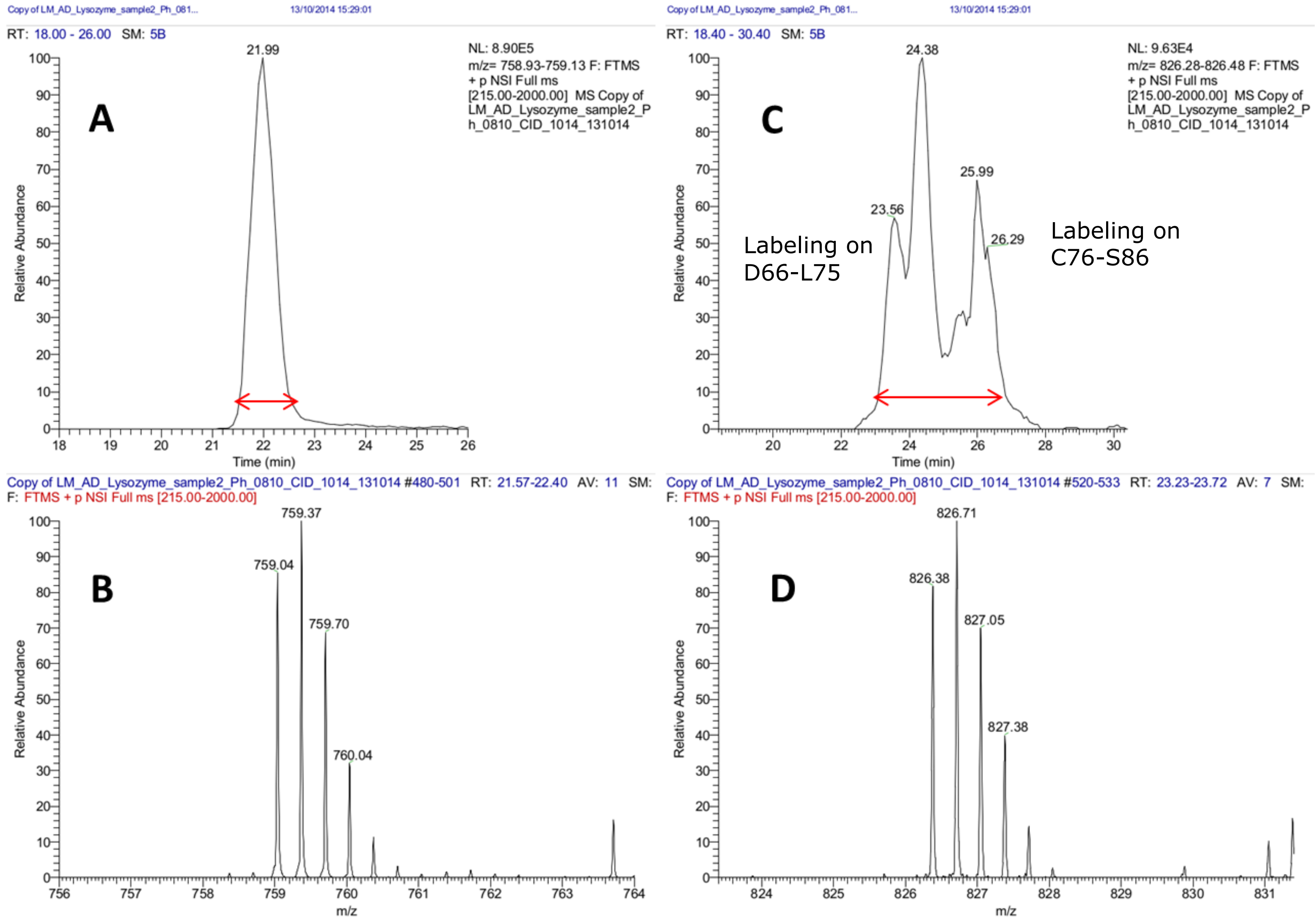

Supplementary Figure 19 – HEWL Peptide D66-S86 XIC and Spectrum: extracted ion chromatograms (XIC) for the unlabeled and labeled precursors (A and C, respectively, mass tolerance *m/z* 0.1), and spectra of the unlabeled and labeled precursor ions (B and D, respectively). The red arrow indicates approximately the area over which the data has been averaged.

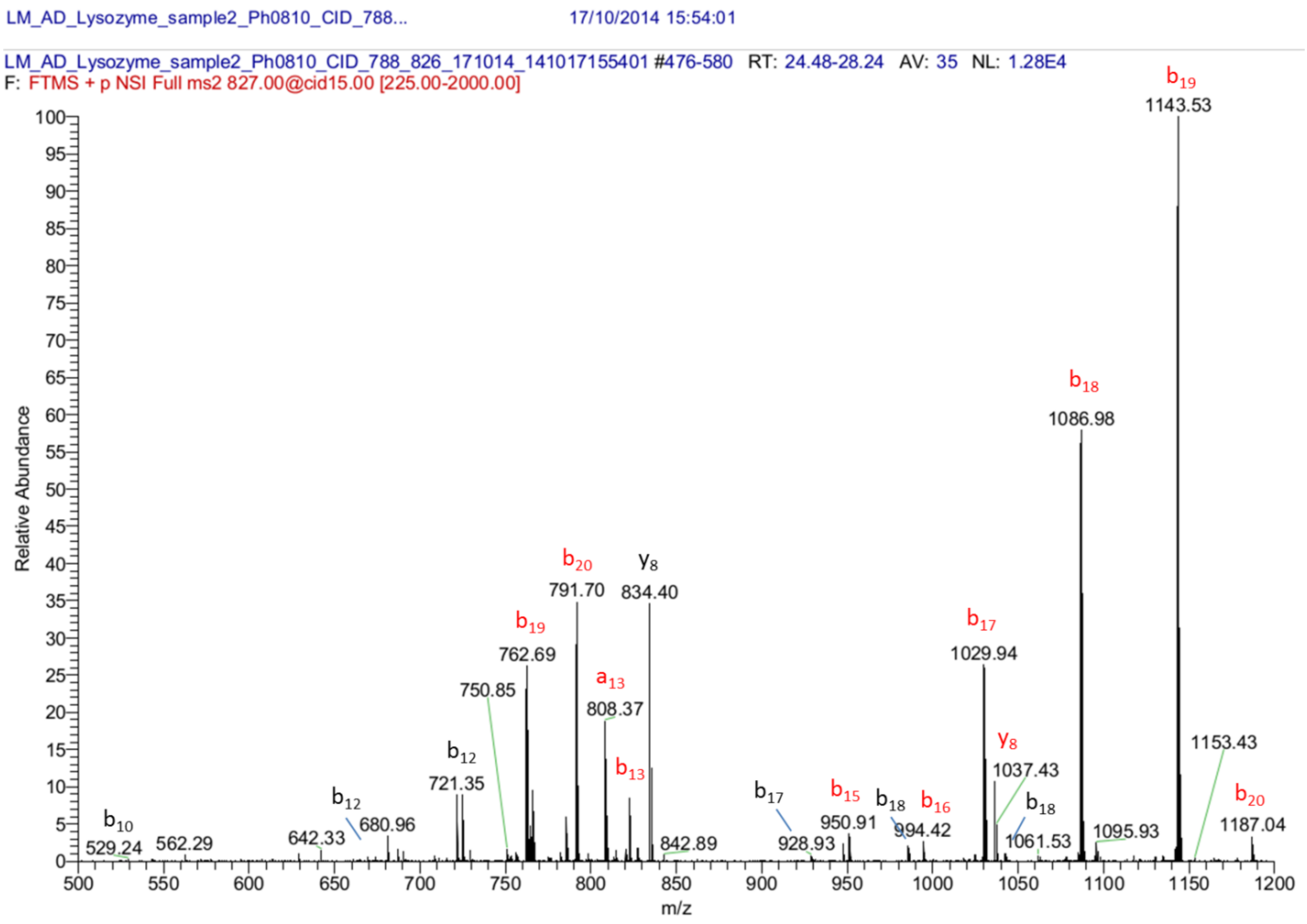

Supplementary Figure 20 – HEWL Labelled Peptide D66-S86 CID Spectrum: example of MS/MS data. Signals labelled in black and red correspond respectively to unlabelled and labelled ions.

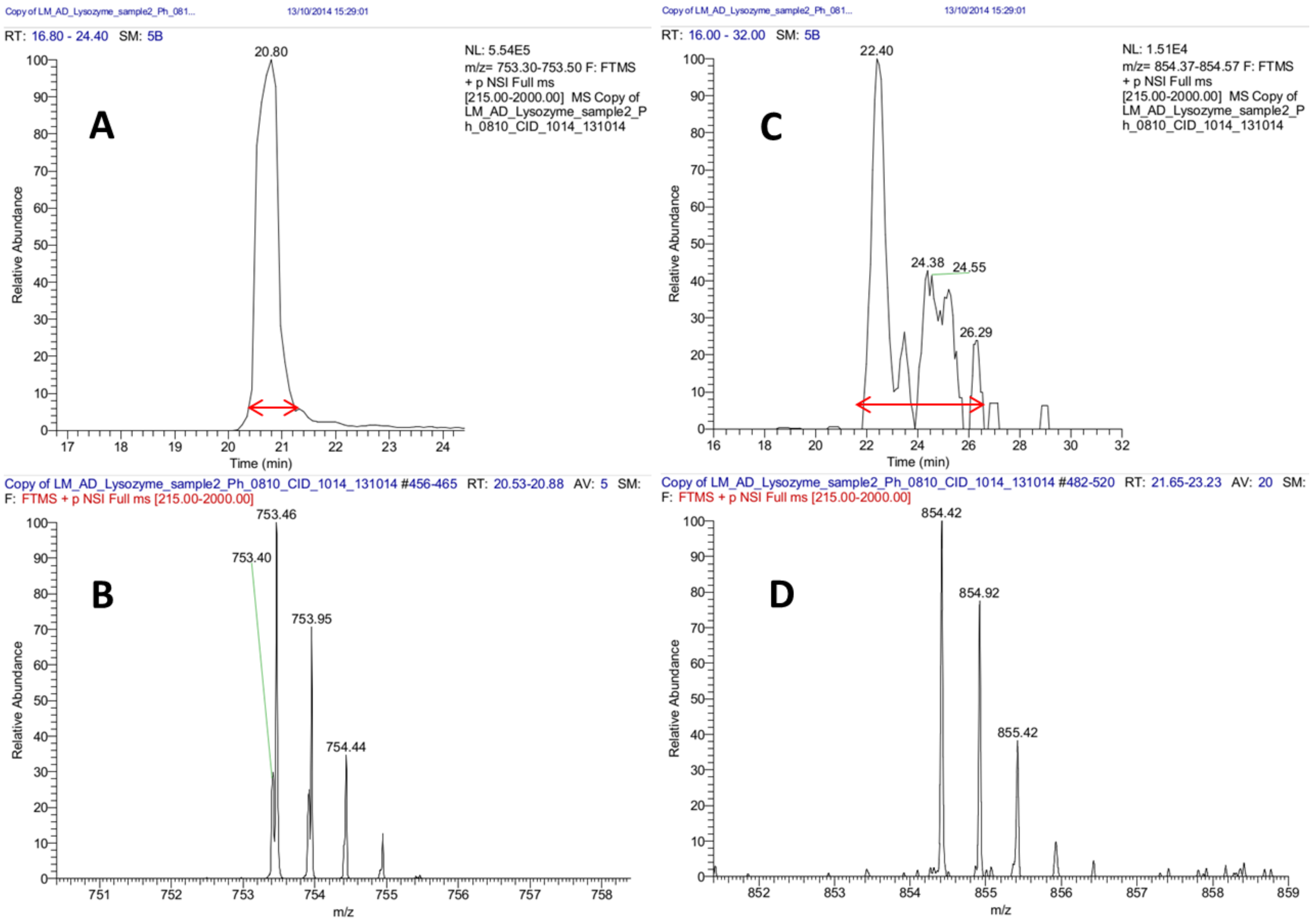

Supplementary Figure 21 – HEWL Peptide D87-S100 XIC and Spectrum: extracted ion chromatograms (XIC) for the unlabeled and labeled precursors (A and C, respectively, mass tolerance *m/z* 0.1), and spectra of the unlabeled and labeled precursor ions (B and D, respectively). The red arrow indicates approximately the area over which the data has been averaged.

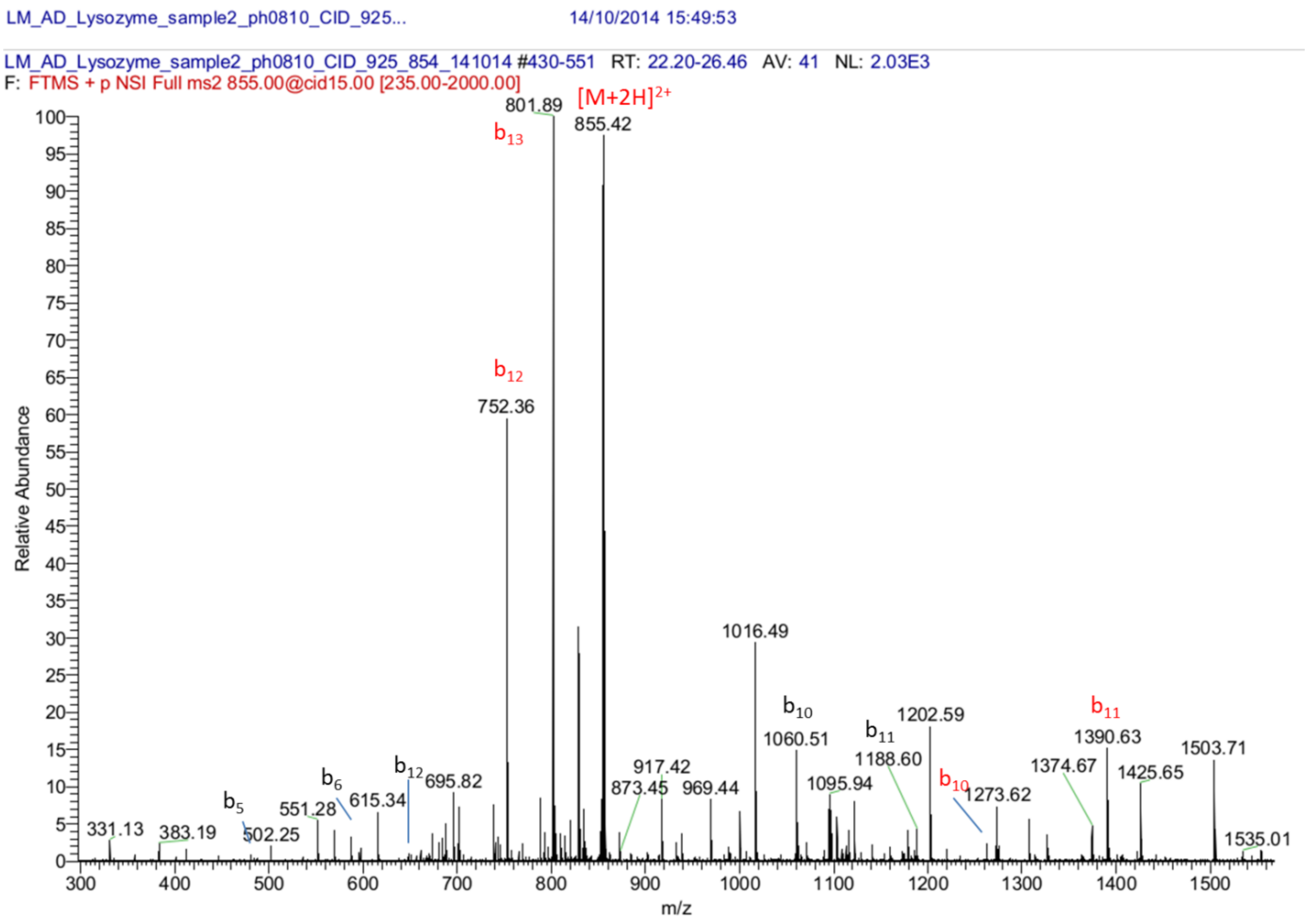

Supplementary Figure 22 – HEWL Labelled Peptide D87-S100 CID Spectrum: example of MS/MS data. Signals labelled in black and red correspond respectively to unlabelled and labelled ions.

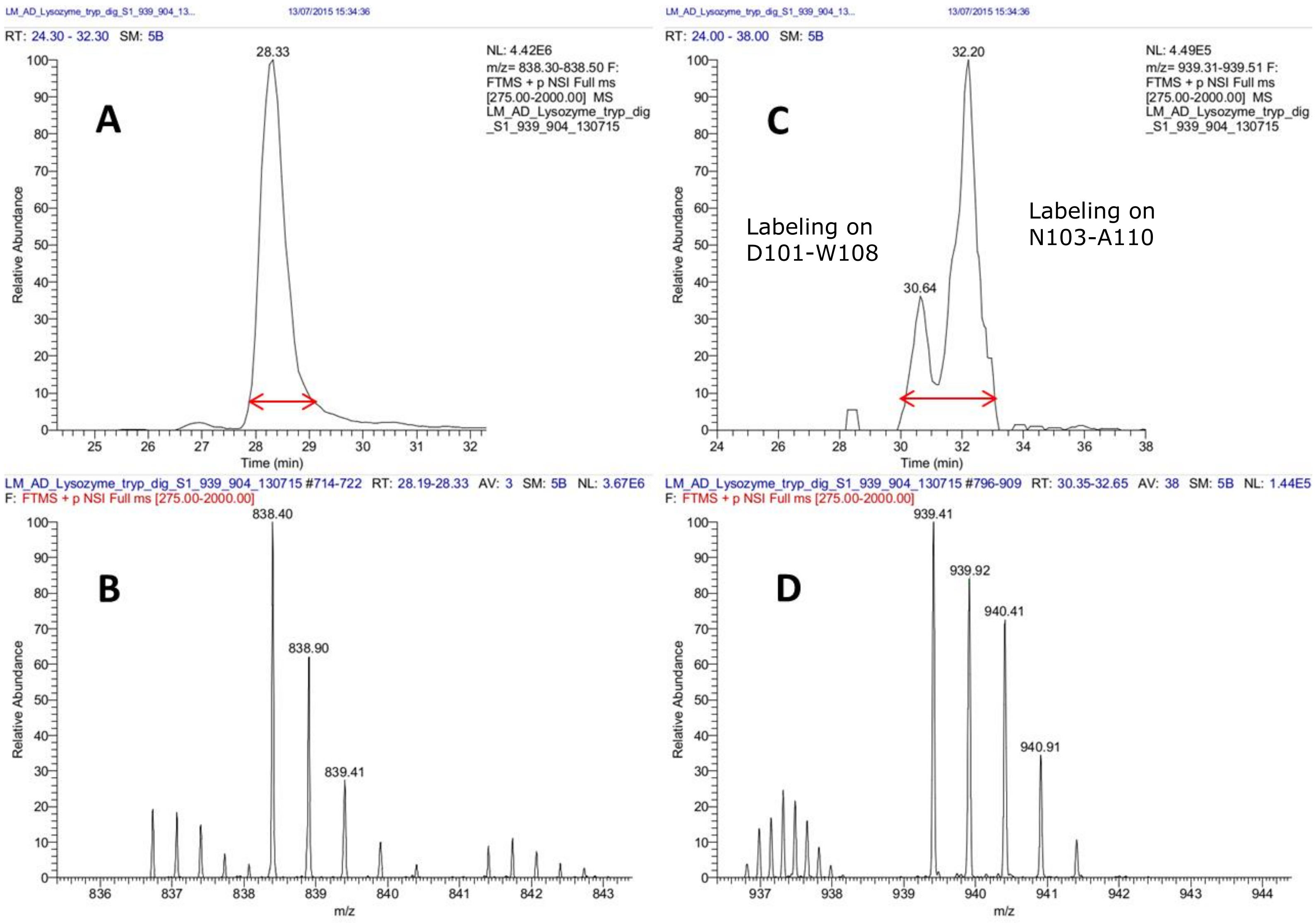

Supplementary Figure 23 – HEWL Peptide I98-R112 XIC and Spectrum: extracted ion chromatograms (XIC) for the unlabeled and labeled precursors (A and C, respectively, mass tolerance *m/z* 0.1), and spectra of the unlabeled and labeled precursor ions (B and D, respectively). The red arrow indicates approximately the area over which the data has been averaged.

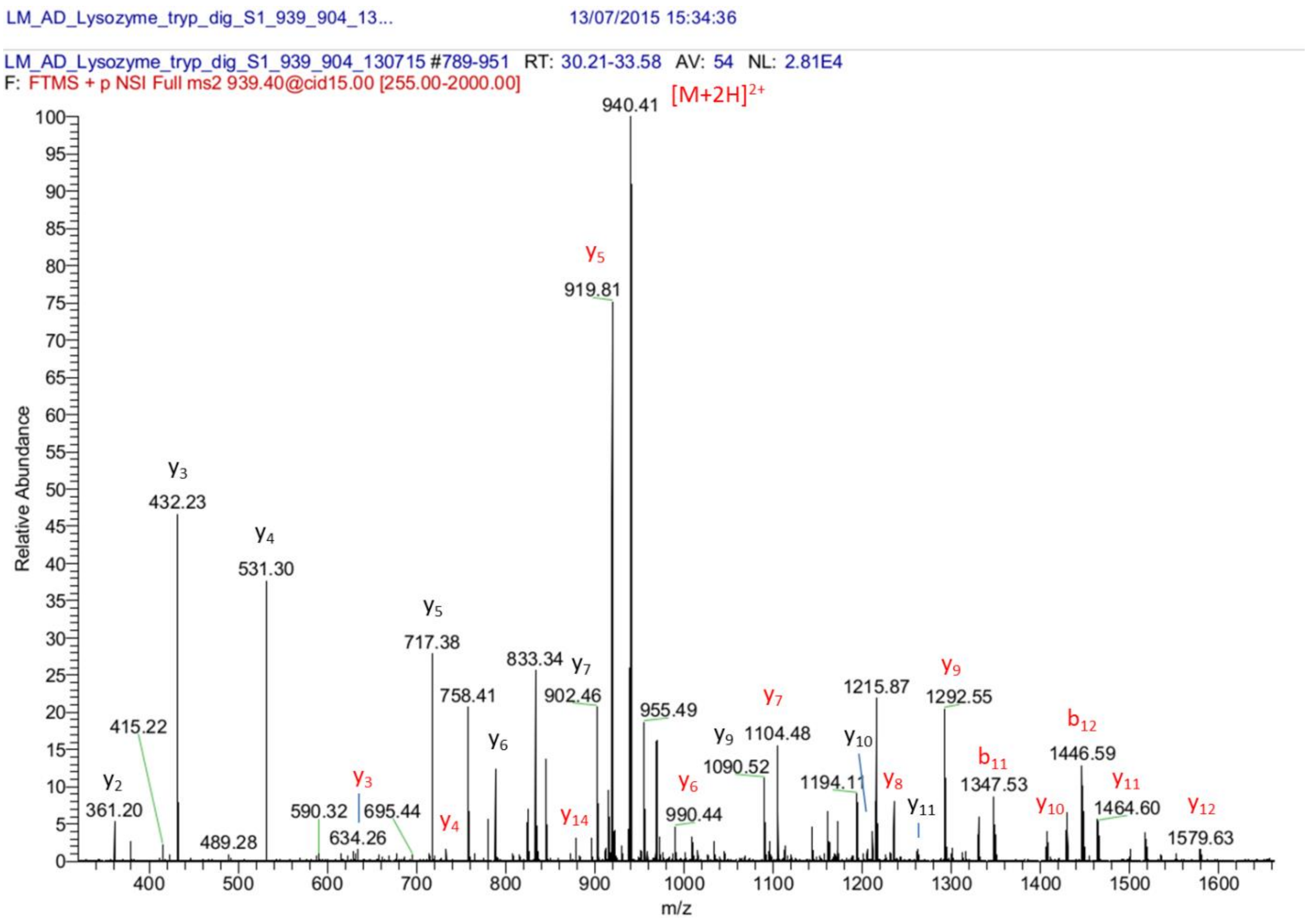

Supplementary Figure 24 – HEWL Labelled Peptide I98-R112 CID Spectrum: example of MS/MS data. Signals labelled in black and red correspond respectively to unlabelled and labelled ions.

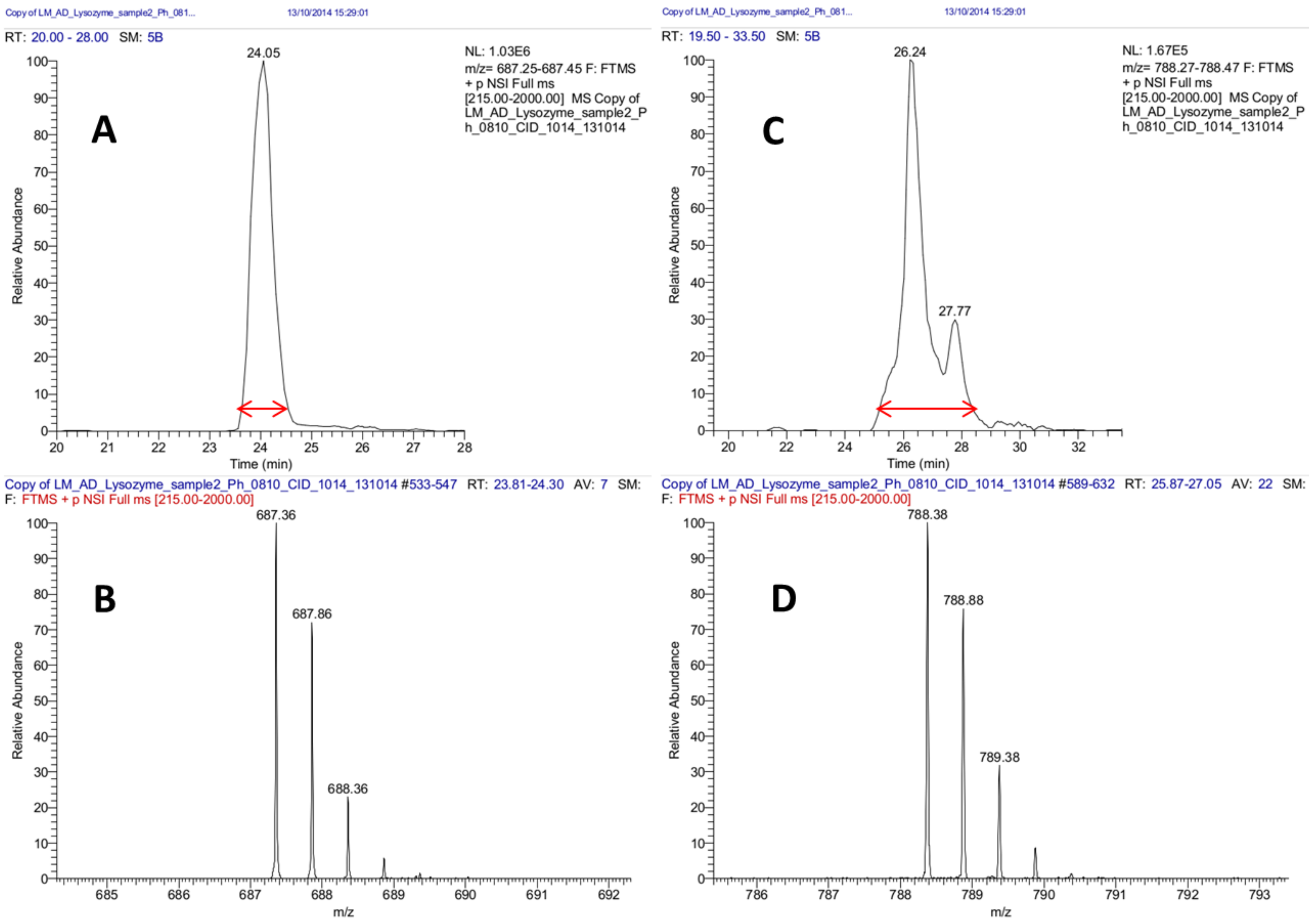

Supplementary Figure 25 – HEWL Peptide D119-L129 XIC and Spectrum: extracted ion chromatograms (XIC) for the unlabeled and labeled precursors (A and C, respectively, mass tolerance *m/z* 0.1), and spectra of the unlabeled and labeled precursor ions (B and D, respectively). The red arrow indicates approximately the area over which the data has been averaged.

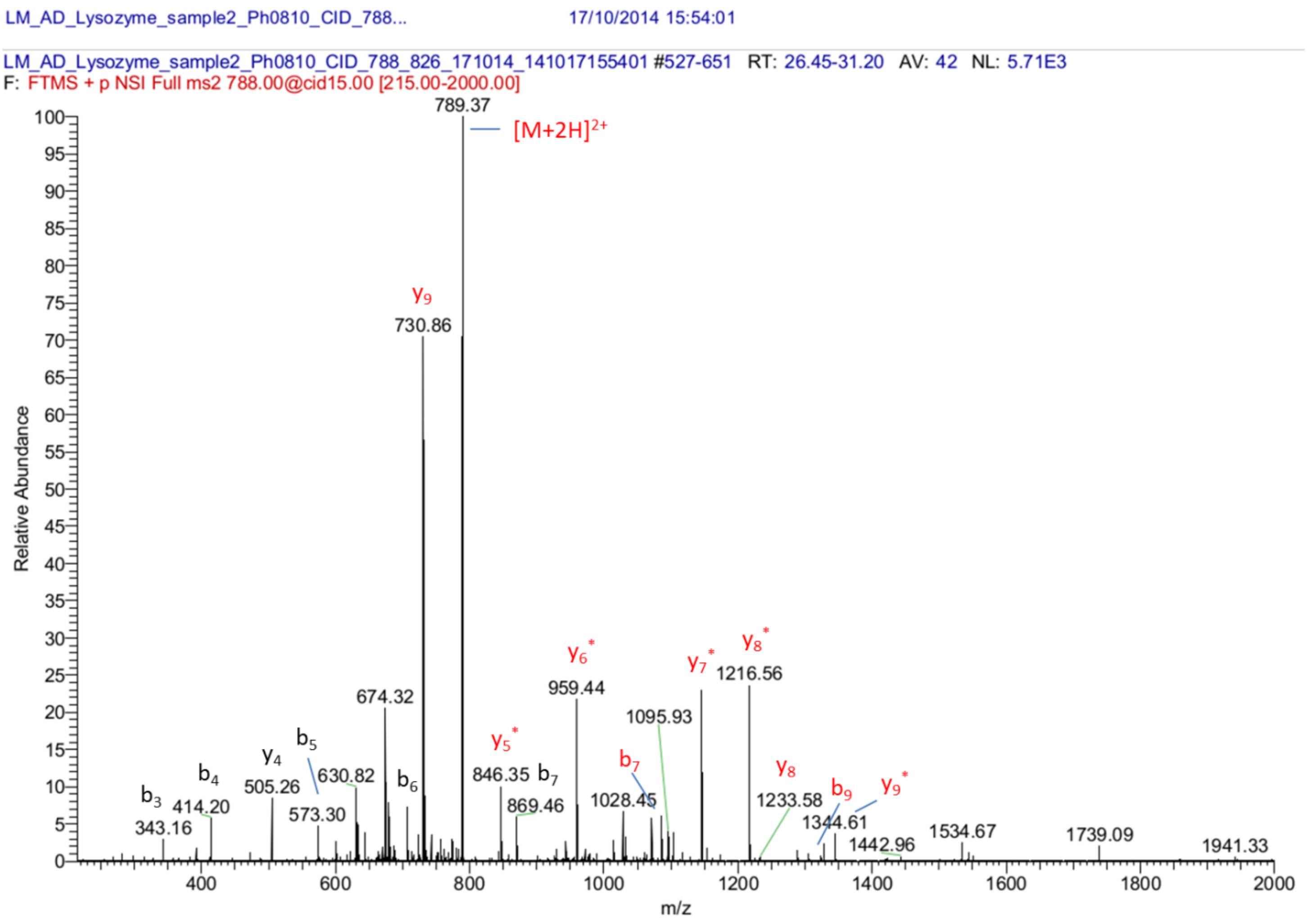

Supplementary Figure 26 – HEWL Labelled Peptide D119-L129 CID Spectrum: example of MS/MS data. Signals labelled in black and red correspond respectively to unlabelled and labelled ions.

GSMAELSEEALLSVLPTIRVPKAGDRVHKDECAFSFDTPESEGGLYICMNTFLGFGKQYVE

RHFNKTGQRVYLHLRRTRRPKEEDPATGTGDPPRKKPTRLAIGVEGGFDLSEEKFELDED

VKIVILPDYLEIARDGLGGLPDIVRDRVTSAVEALLSADSASRKQEVQAWDGEVRQVSKH

AFSLKQLDNPARIPPCGWKCSKCDMRENLWLNLTDGSILCGRRYFDGSGGNNHAVEHY

RETGYPLAVKLGTITPDGADVYSYDEDDMVLDPSLAEHLSHFGIDMLKMQKTDKTMTEL

EIDMNQRIGEWELIQESGVPLKPLFGPGYTGIRNLGNSAYLNSVVQVLFSIPDFQRKYVD

KLEKIFQNAPTDPTQDFSTQVAKLGHGLLSGEYSKPVPESGDGERVPEQKEVQDGIAPR

MFKALIGKGHPEFSTNRQQDAQEFFLHLINMVERNCRSSENPNEVFRFLVEEKIKCLATEK

VKYTQRVDYIMQLPVPMDAALNKEELLEYEEKKRQAEEEKMALPELVRAQVPFSSCLEAY

GAPEQVDDFWSTALQAKSVAVKTTRFASFPDYLVIQIKKFTFGLDWVPKKLDVSIEMPEE

LDISQLRGTGLQPGEEELPDIAPPLVTPDEPKAPMLDESVIIQLVEMGFPMDACRKAVYY

TGNSGAEAAMNWVMSHMDDPDFANPLILPGSSGPGSTSAAADPPPEDCVTTIVSMGF

SRDQALKALRATNNSLERAVDWIFSHIDDLDAEAAMDISEGRSAADSISESVPVGPKVRD

GPGKYQLFAFISHMGTSTMCGHYVCHIKKEGRWVIYNDQKVCASEKPPKDLGYIYFYQR

VA

Supplementary Figure 27 – USP5 Sequence Coverage. Sequence coverage obtained for USP5 from tryptic peptides (red arrows).

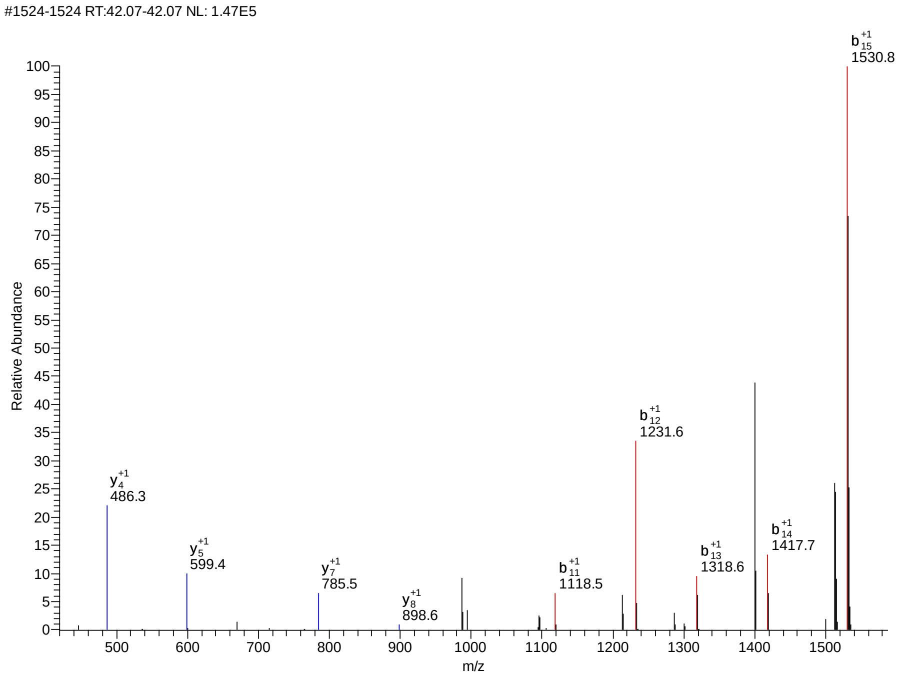

Supplementary Figure 28 – USP5 peptide G1-R19: CID MS/MS spectra of (unlabeled) USP5 tryptic peptides with assignments from BioWorks 3 (ThermoFisher) database searching used to confirm peptide identity.

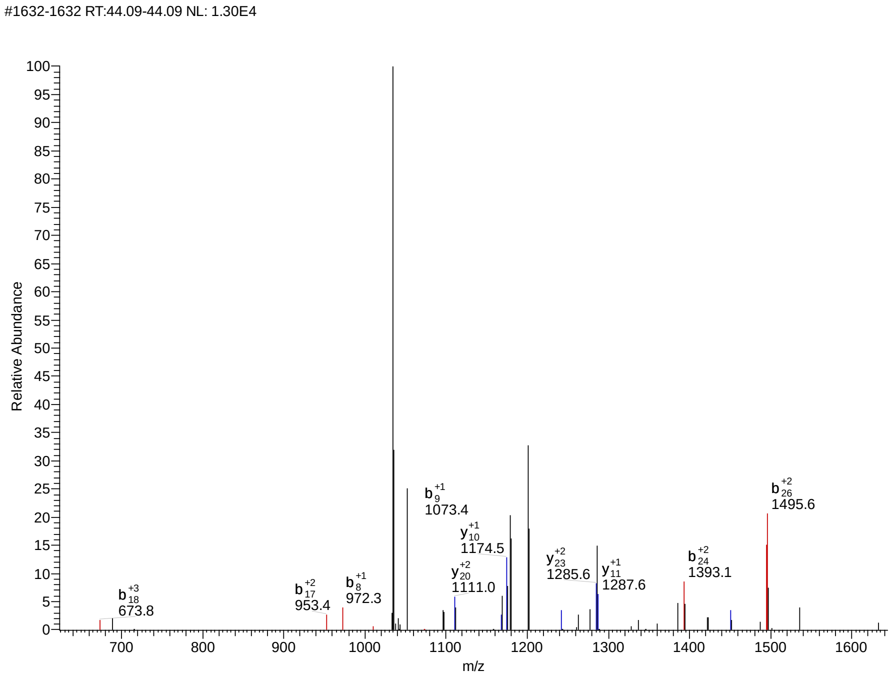

Supplementary Figure 29 – USP5 peptide D30-K57: CID MS/MS spectra of (unlabeled) USP5 tryptic peptides with assignments from BioWorks 3 (ThermoFisher) database searching used to confirm peptide identity.

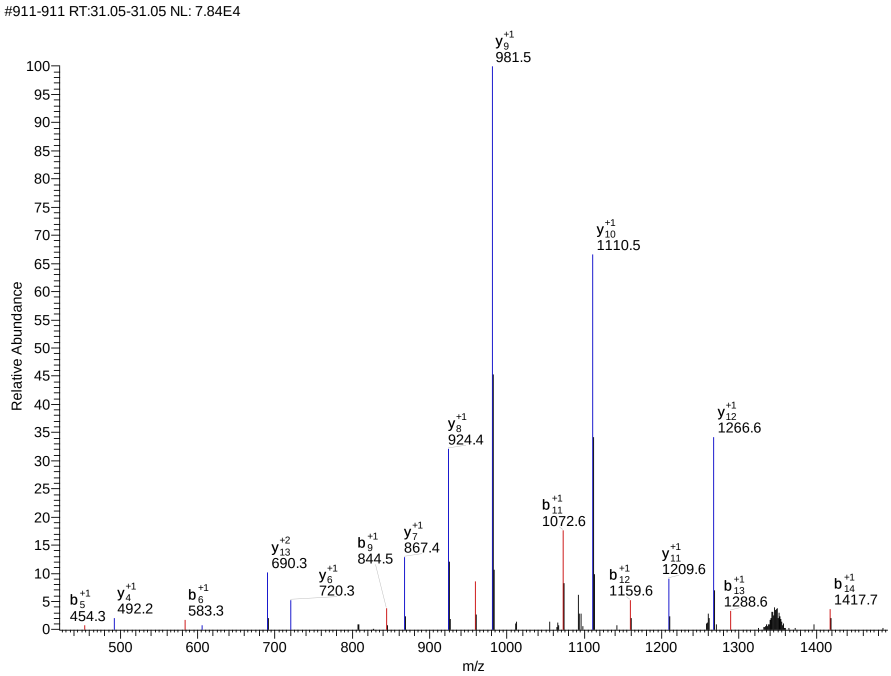

Supplementary Figure 30 – USP5 peptide L101-K115: CID MS/MS spectra of (unlabeled) USP5 tryptic peptides with assignments from BioWorks 3 (ThermoFisher) database searching used to confirm peptide identity.

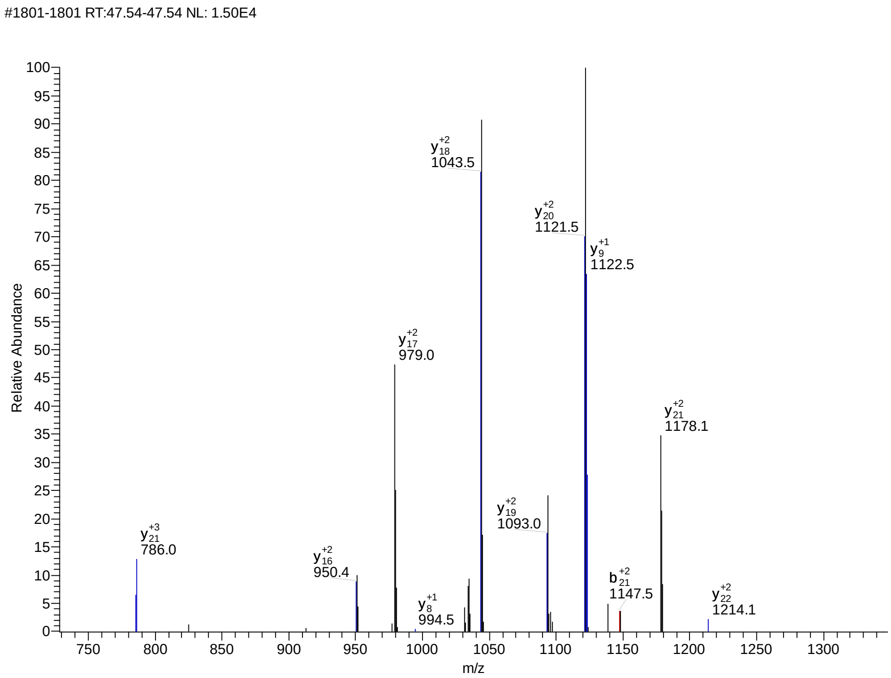

Supplementary Figure 31 – USP5 peptide L101-K123: CID MS/MS spectra of (unlabeled) USP5 tryptic peptides with assignments from BioWorks 3 (ThermoFisher) database searching used to confirm peptide identity.

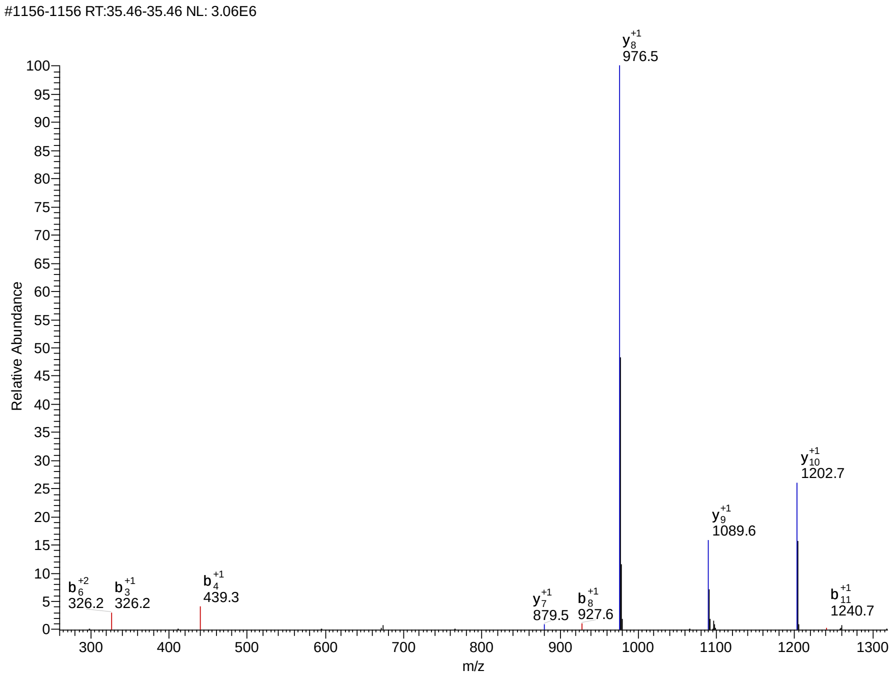

Supplementary Figure 32 – USP5 peptide I124-R135: CID MS/MS spectra of (unlabeled) USP5 tryptic peptides with assignments from BioWorks 3 (ThermoFisher) database searching used to confirm peptide identity.

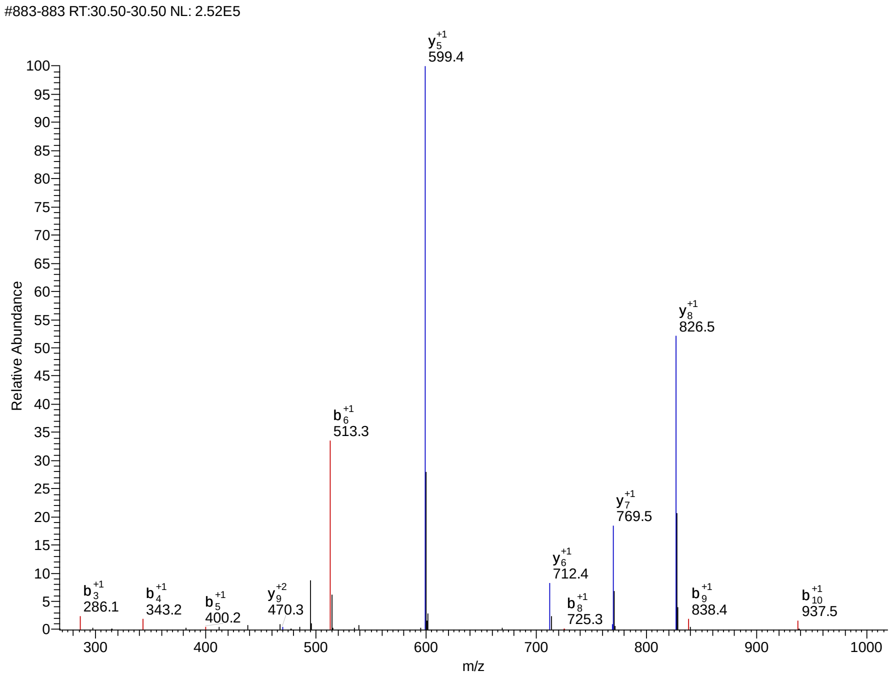

Supplementary Figure 33 – USP5 peptide D136-R146: CID MS/MS spectra of (unlabeled) USP5 tryptic peptides with assignments from BioWorks 3 (ThermoFisher) database searching used to confirm peptide identity.

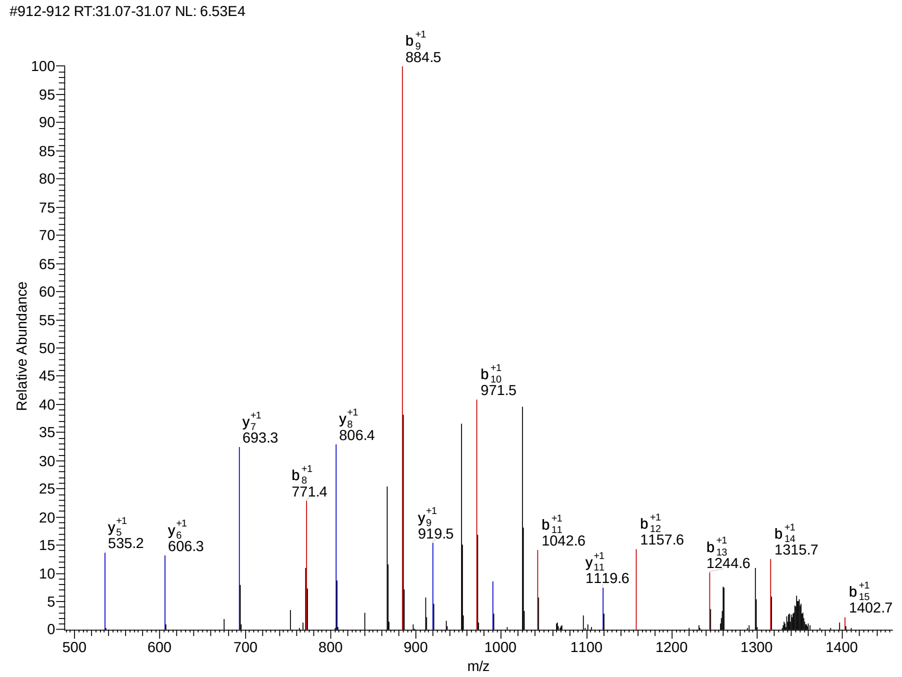

Supplementary Figure 34 – USP5 peptide V149-R164: CID MS/MS spectra of (unlabeled) USP5 tryptic peptides with assignments from BioWorks 3 (ThermoFisher) database searching used to confirm peptide identity.

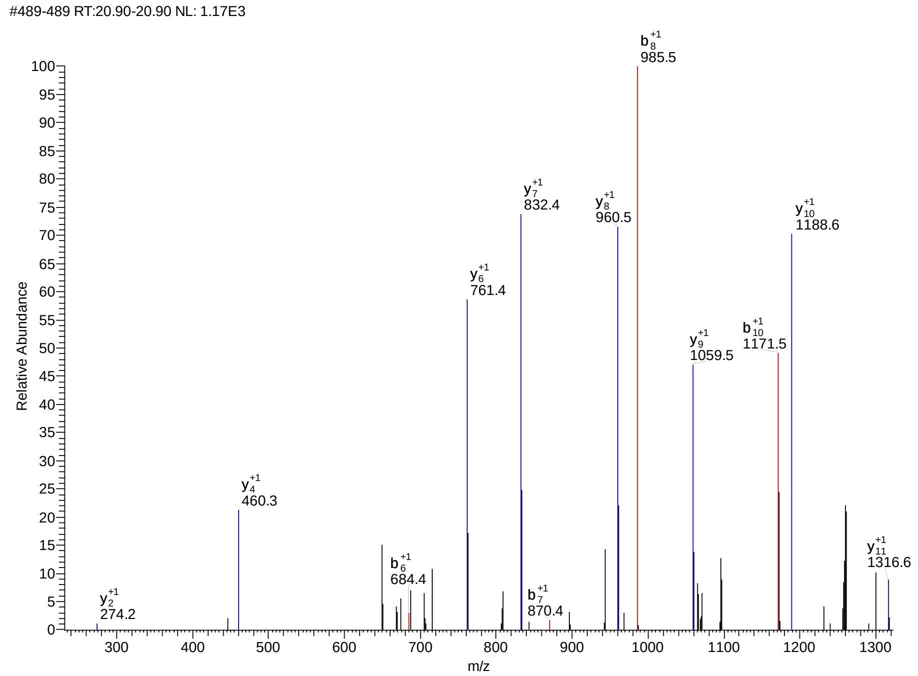

Supplementary Figure 35 – USP5 peptide K165-R176: CID MS/MS spectra of (unlabeled) USP5 tryptic peptides with assignments from BioWorks 3 (ThermoFisher) database searching used to confirm peptide identity.

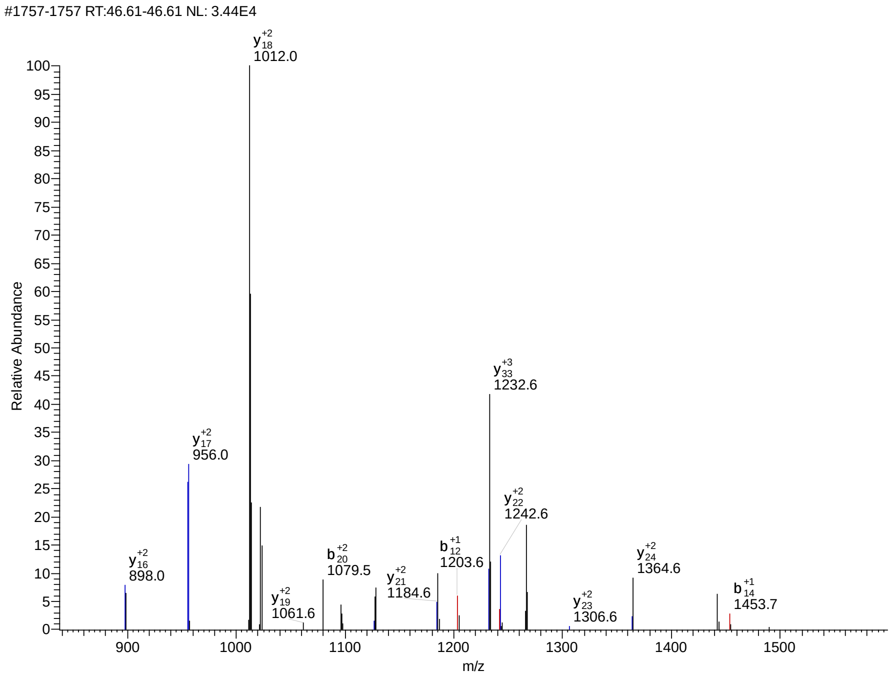

Supplementary Figure 36 – USP5 peptide L250-K287: CID MS/MS spectra of (unlabeled) USP5 tryptic peptides with assignments from BioWorks 3 (ThermoFisher) database searching used to confirm peptide identity.

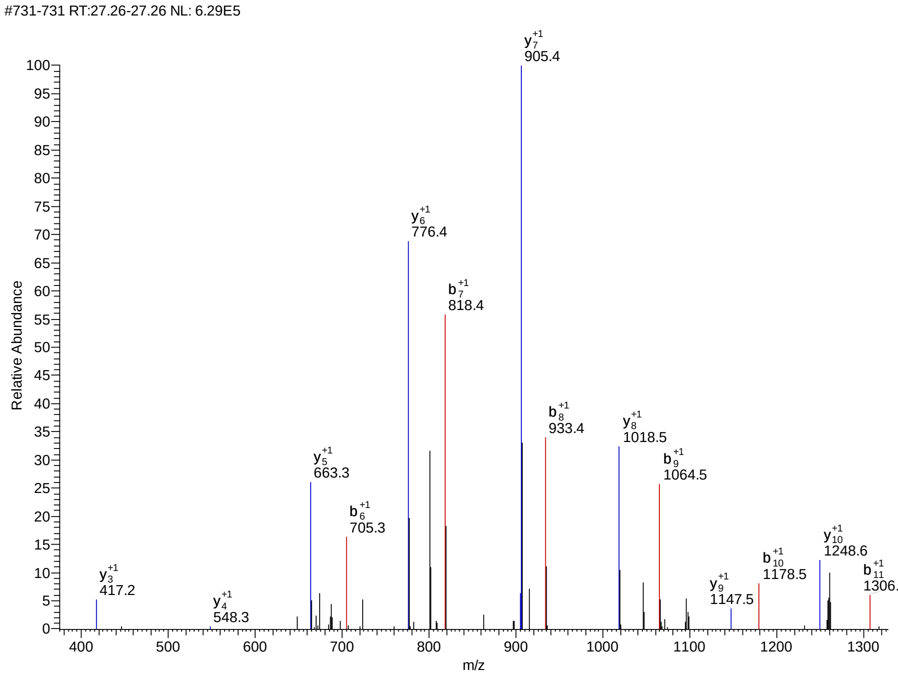

Supplementary Figure 37 – USP5 peptide T294-R305: CID MS/MS spectra of (unlabeled) USP5 tryptic peptides with assignments from BioWorks 3 (ThermoFisher) database searching used to confirm peptide identity.

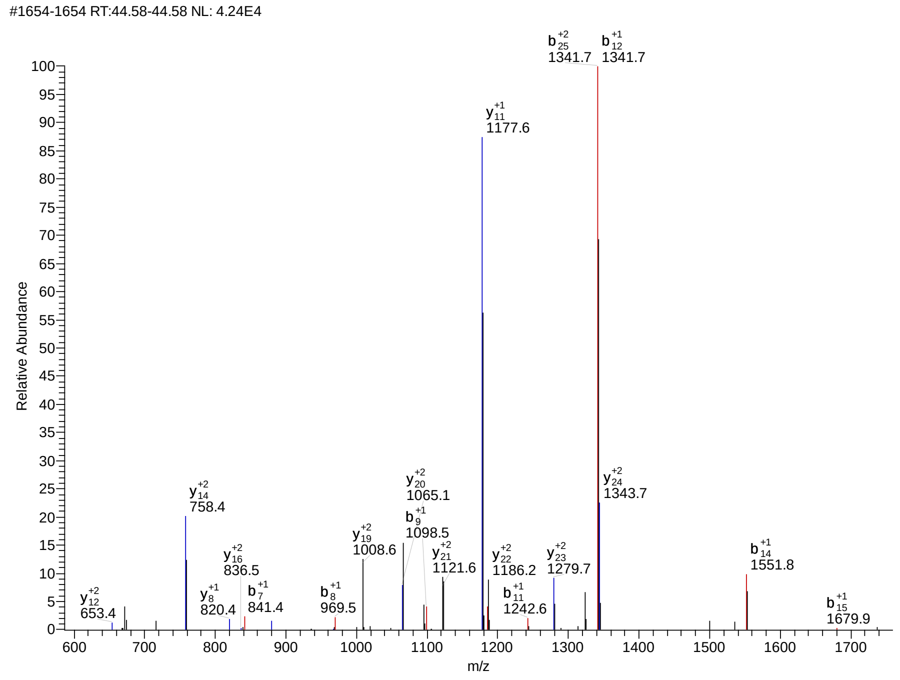

Supplementary Figure 38 – USP5 peptide I306-R331: CID MS/MS spectra of (unlabeled) USP5 tryptic peptides with assignments from BioWorks 3 (ThermoFisher) database searching used to confirm peptide identity.

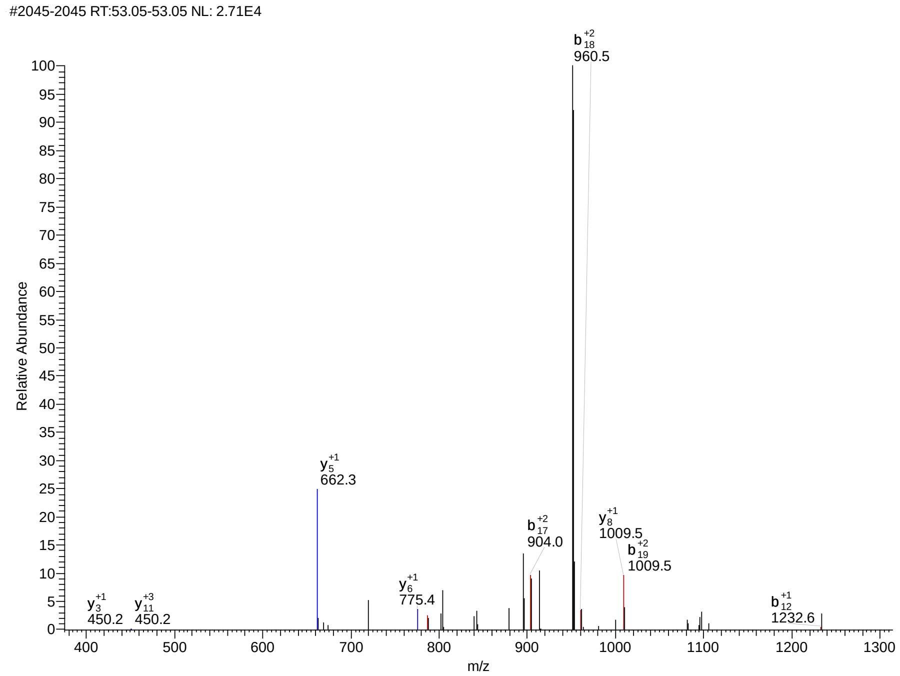

Supplementary Figure 39 – USP5 peptide N332-R354: CID MS/MS spectra of (unlabeled) USP5 tryptic peptides with assignments from BioWorks 3 (ThermoFisher) database searching used to confirm peptide identity.

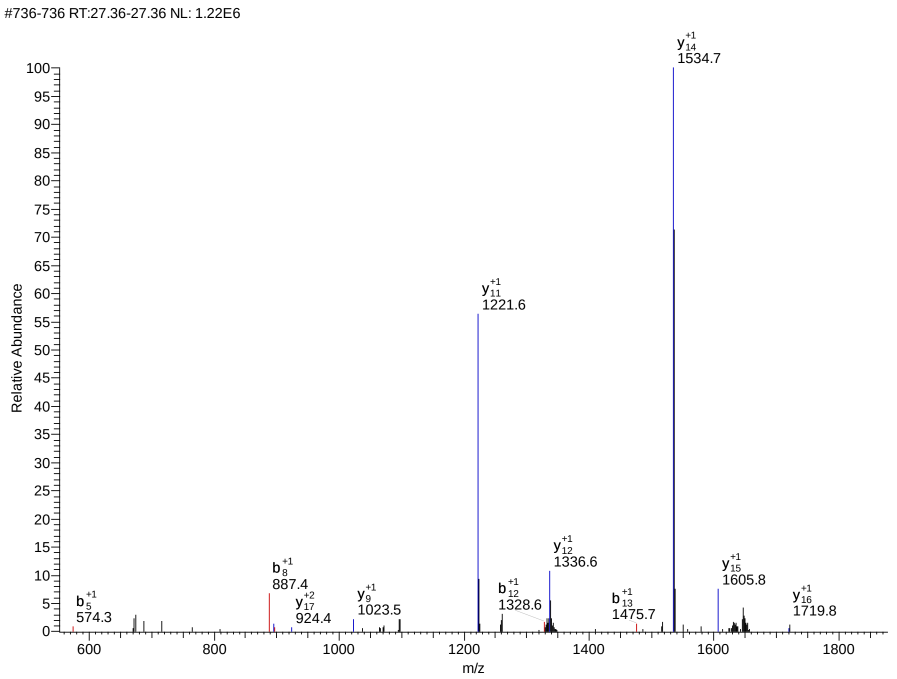

Supplementary Figure 40 – USP5 peptideI363-K381: CID MS/MS spectra of (unlabeled) USP5 tryptic peptides with assignments from BioWorks 3 (ThermoFisher) database searching used to confirm peptide identity.

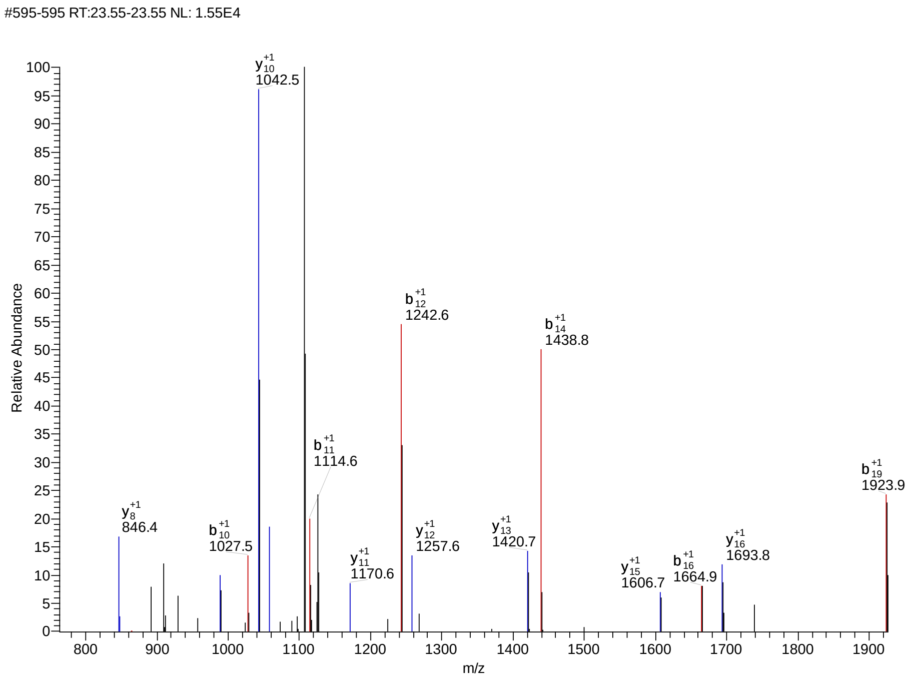

Supplementary Figure 41 – USP5 peptide L382-R403: CID MS/MS spectra of (unlabeled) USP5 tryptic peptides with assignments from BioWorks 3 (ThermoFisher) database searching used to confirm peptide identity.

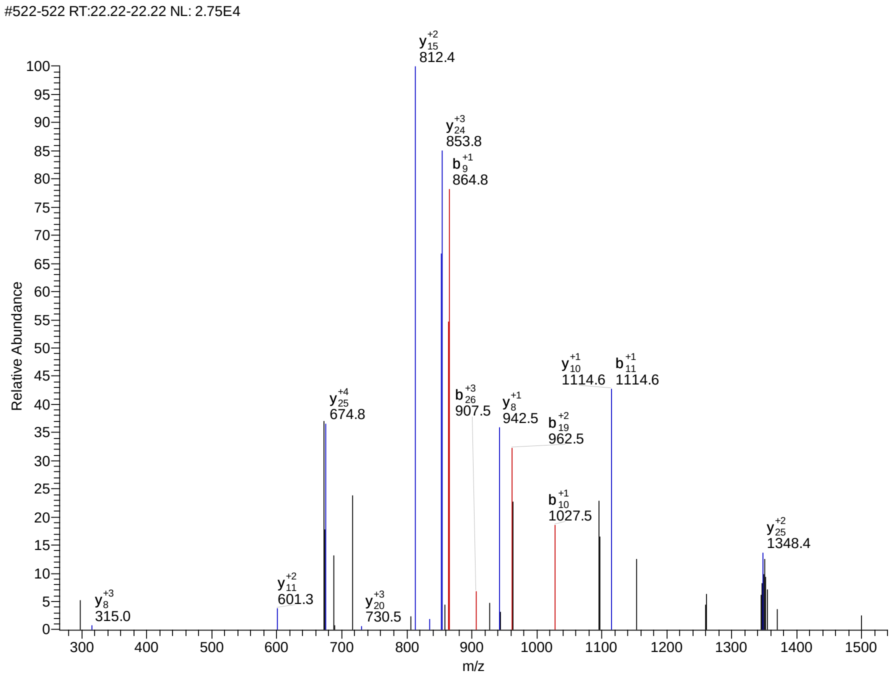

Supplementary Figure 42 – USP5 peptide L382-R408: CID MS/MS spectra of (unlabeled) USP5 tryptic peptides with assignments from BioWorks 3 (ThermoFisher) database searching used to confirm peptide identity.

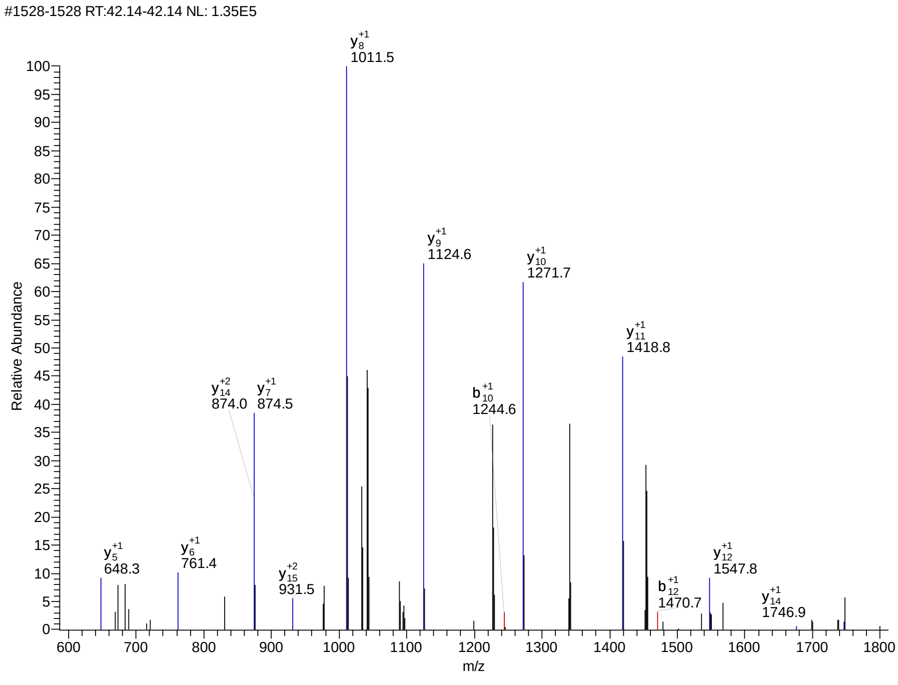

Supplementary Figure 43 – USP5 peptide Q435-R451: CID MS/MS spectra of (unlabeled) USP5 tryptic peptides with assignments from BioWorks 3 (ThermoFisher) database searching used to confirm peptide identity.

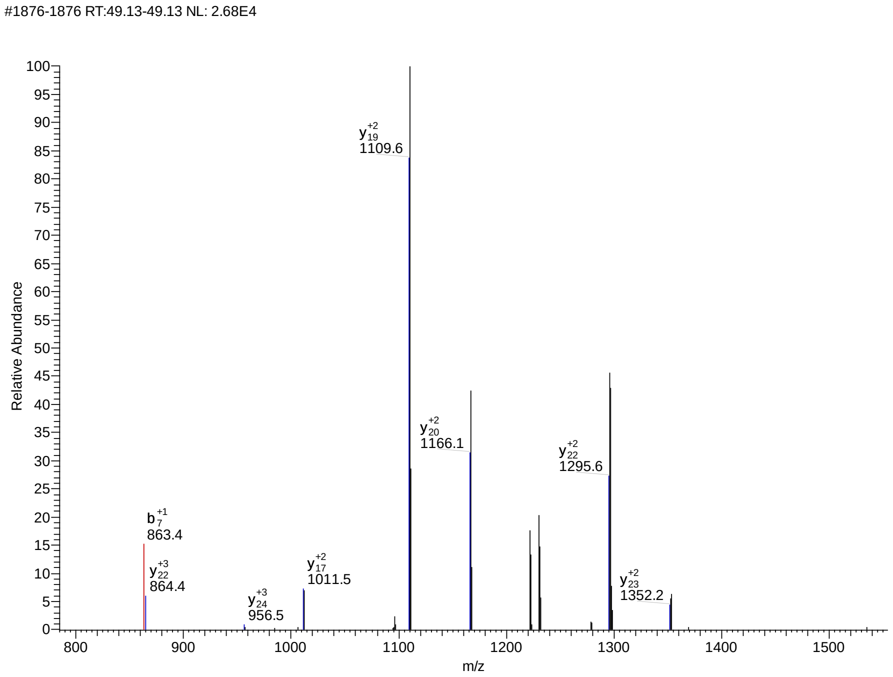

Supplementary Figure 44 – USP5 peptide V485-K510: CID MS/MS spectra of (unlabeled) USP5 tryptic peptides with assignments from BioWorks 3 (ThermoFisher) database searching used to confirm peptide identity.

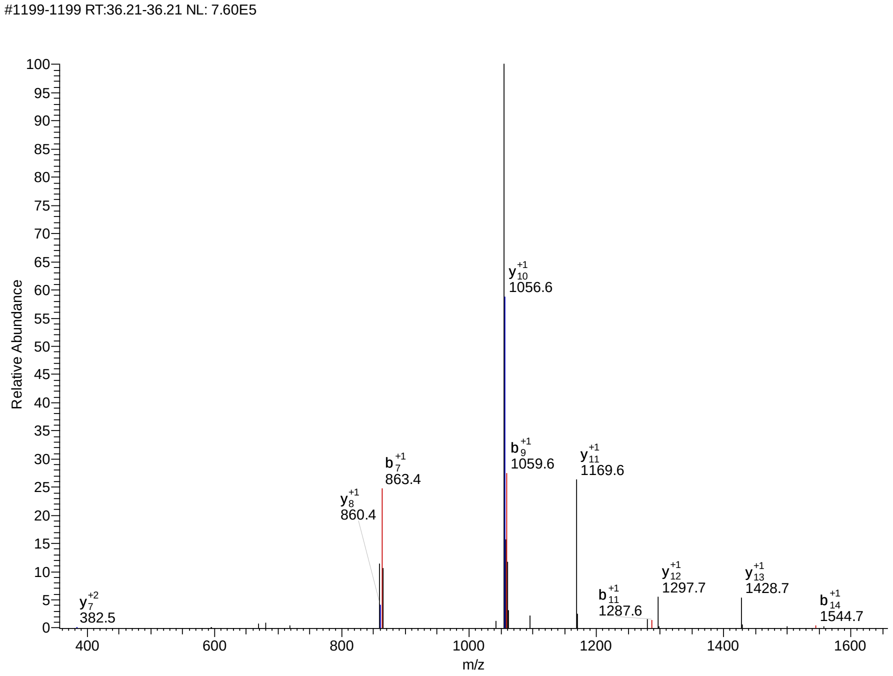

Supplementary Figure 45 – USP5 peptide V485-K501: CID MS/MS spectra of (unlabeled) USP5 tryptic peptides with assignments from BioWorks 3 (ThermoFisher) database searching used to confirm peptide identity.

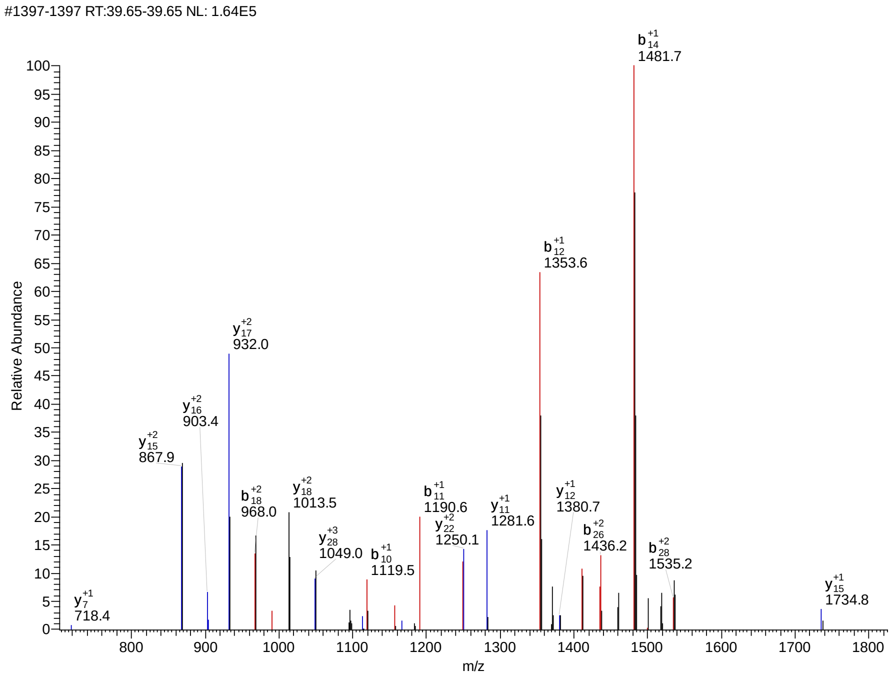

Supplementary Figure 46 – USP5 peptide A527-K555: CID MS/MS spectra of (unlabeled) USP5 tryptic peptides with assignments from BioWorks 3 (ThermoFisher) database searching used to confirm peptide identity.

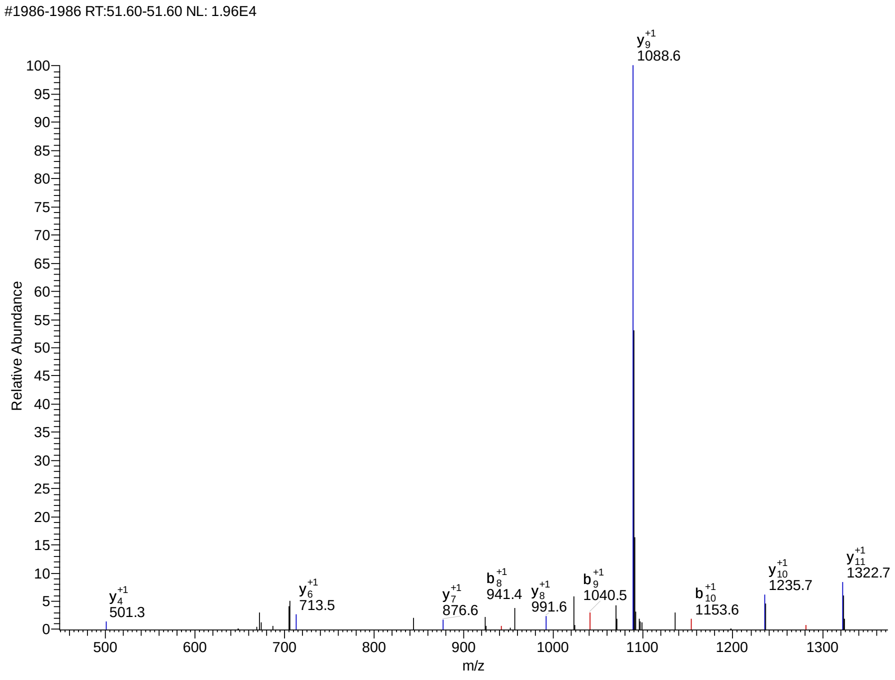

Supplementary Figure 47 – USP5 peptide F564-K576: CID MS/MS spectra of (unlabeled) USP5 tryptic peptides with assignments from BioWorks 3 (ThermoFisher) database searching used to confirm peptide identity.

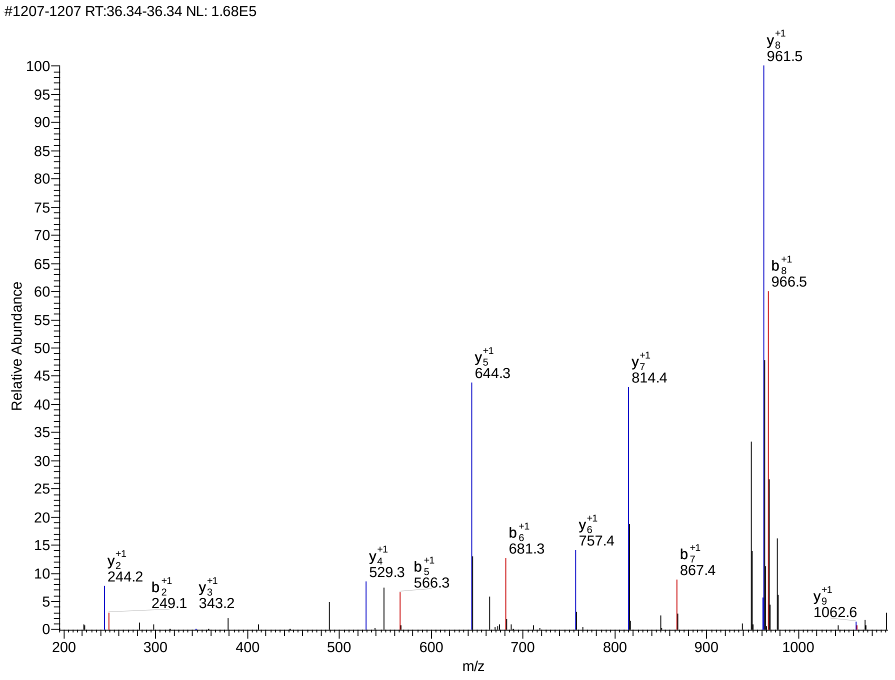

Supplementary Figure 48 – USP5 peptide F578-K587: CID MS/MS spectra of (unlabeled) USP5 tryptic peptides with assignments from BioWorks 3 (ThermoFisher) database searching used to confirm peptide identity.

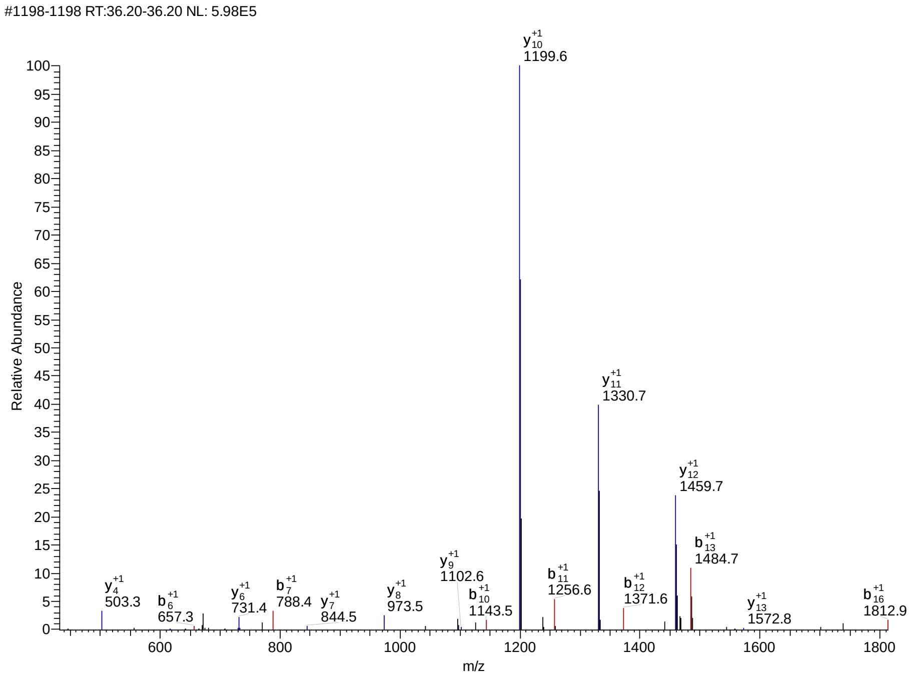

Supplementary Figure 49 – USP5 peptide L589-R605: CID MS/MS spectra of (unlabeled) USP5 tryptic peptides with assignments from BioWorks 3 (ThermoFisher) database searching used to confirm peptide identity.

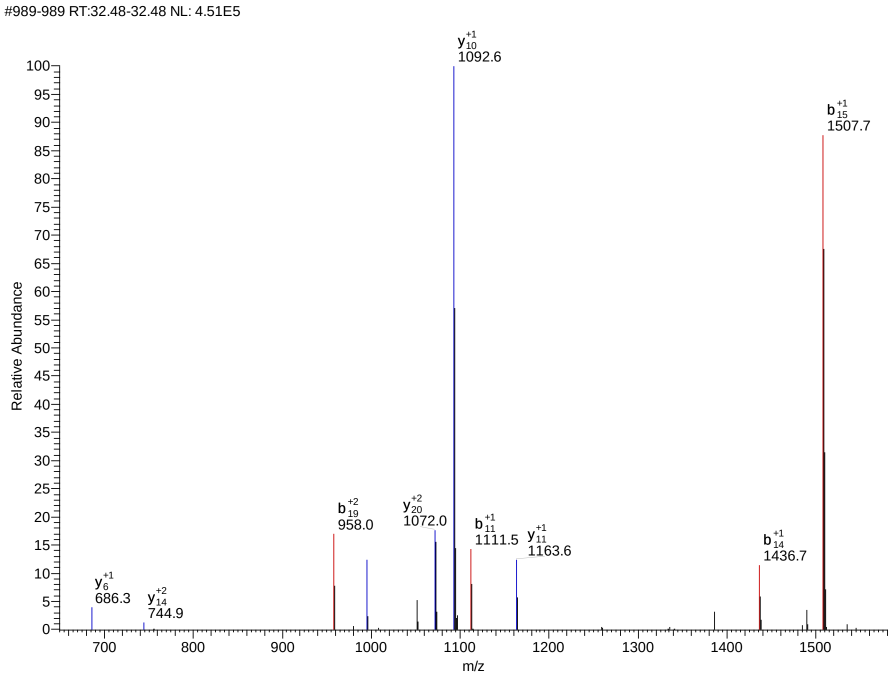

Supplementary Figure 50 – USP5 peptide G606-K630: CID MS/MS spectra of (unlabeled) USP5 tryptic peptides with assignments from BioWorks 3 (ThermoFisher) database searching used to confirm peptide identity.

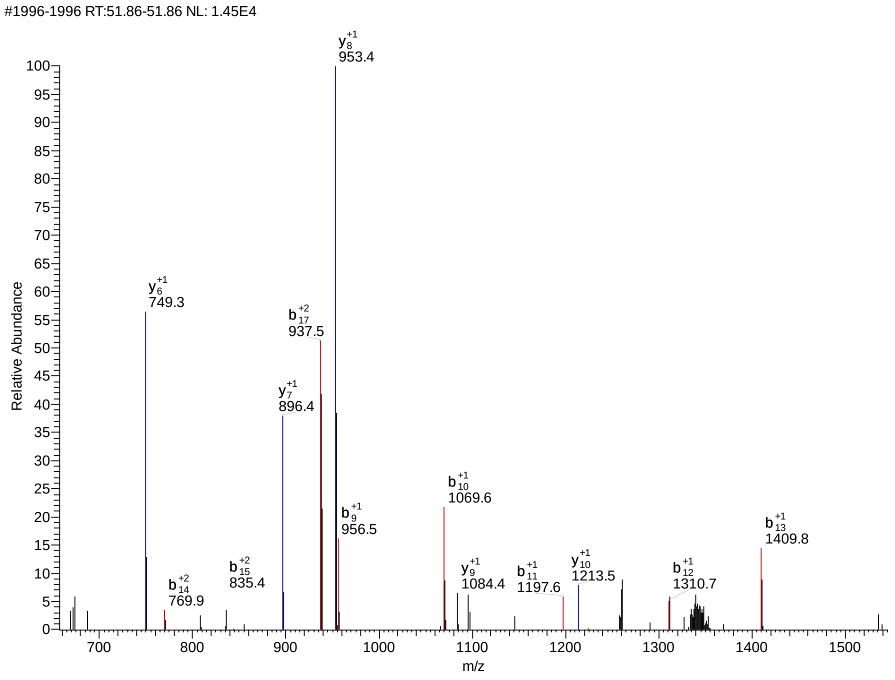

Supplementary Figure 51 – USP5 peptide A631-R653: CID MS/MS spectra of (unlabeled) USP5 tryptic peptides with assignments from BioWorks 3 (ThermoFisher) database searching used to confirm peptide identity.

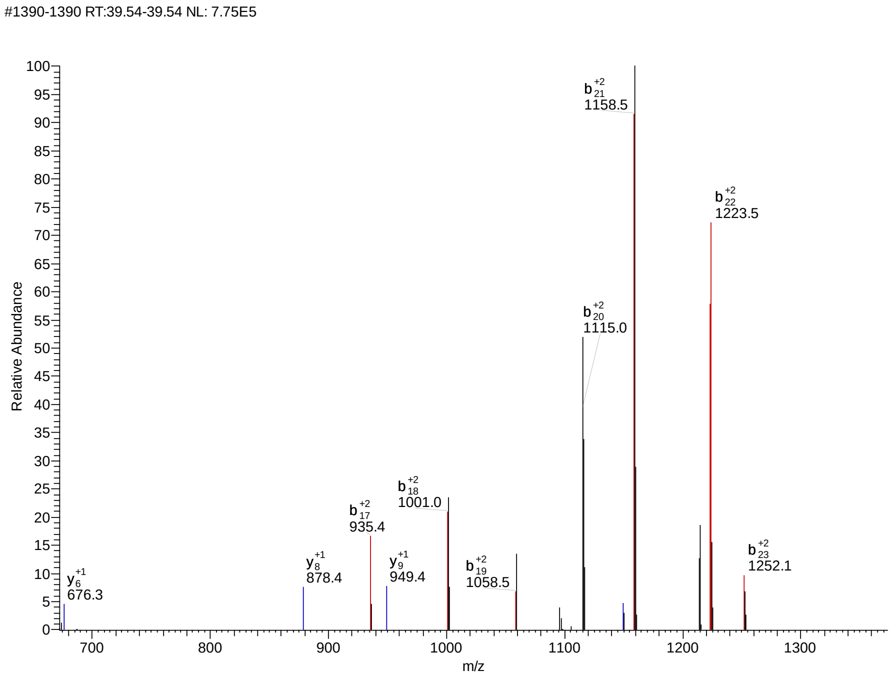

Supplementary Figure 52 – USP5 peptide A734-R757: CID MS/MS spectra of (unlabeled) USP5 tryptic peptides with assignments from BioWorks 3 (ThermoFisher) database searching used to confirm peptide identity.

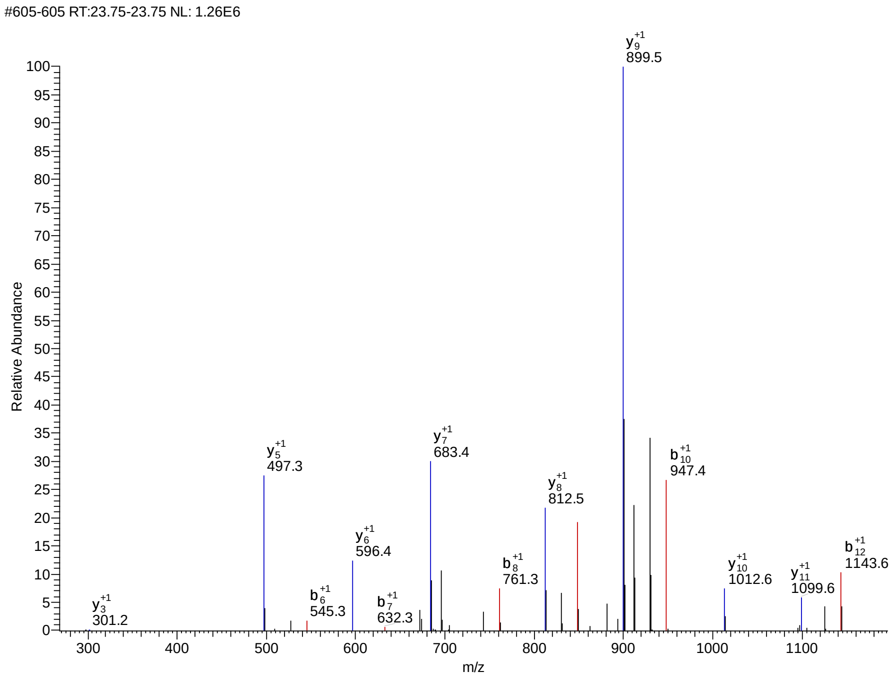

Supplementary Figure 53 – USP5 peptide S758-K772: CID MS/MS spectra of (unlabeled) USP5 tryptic peptides with assignments from BioWorks 3 (ThermoFisher) database searching used to confirm peptide identity.

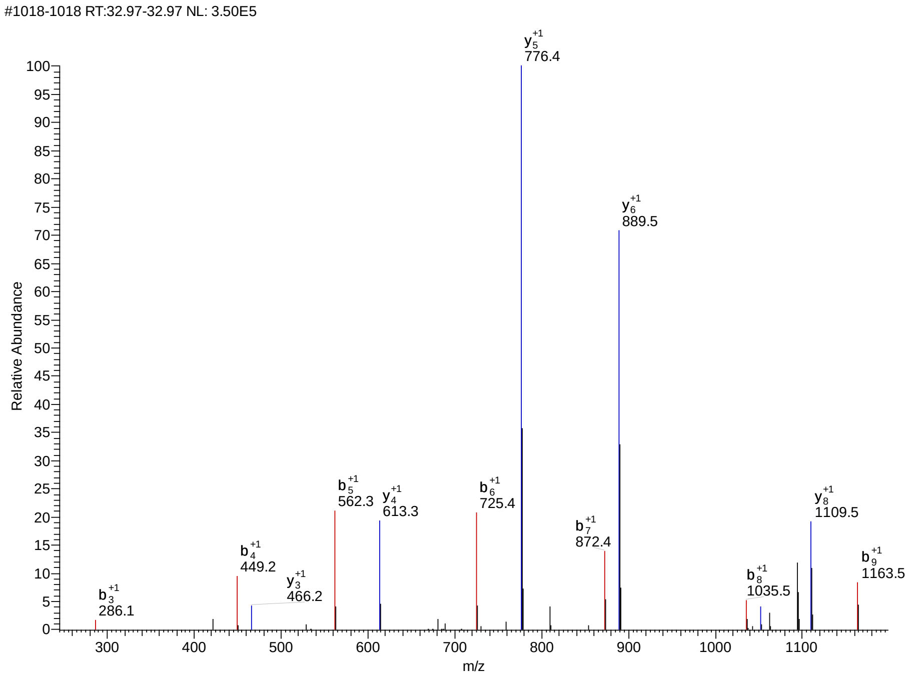

Supplementary Figure 54 – USP5 peptide S758-K772: CID MS/MS spectra of (unlabeled) USP5 tryptic peptides with assignments from BioWorks 3 (ThermoFisher) database searching used to confirm peptide identity.

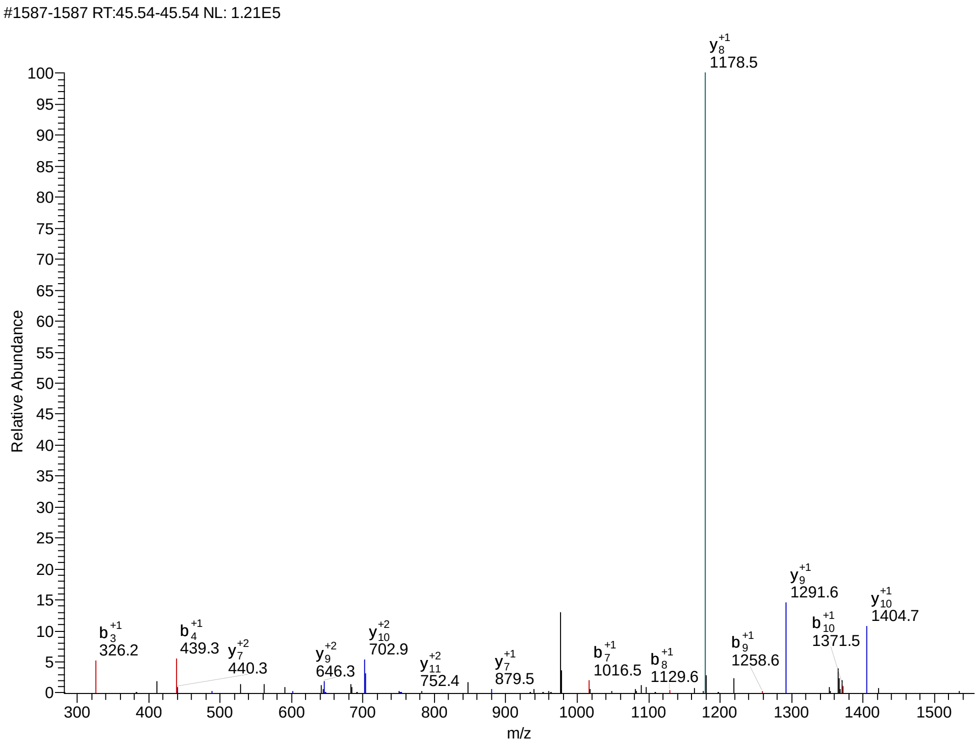

Supplementary Figure 55 – Labelled USP5 peptide I124-R135: CID MS/MS spectra of (labeled) USP5 tryptic peptides with assignments from BioWorks 3 (ThermoFisher) database searching used to confirm peptide identity.

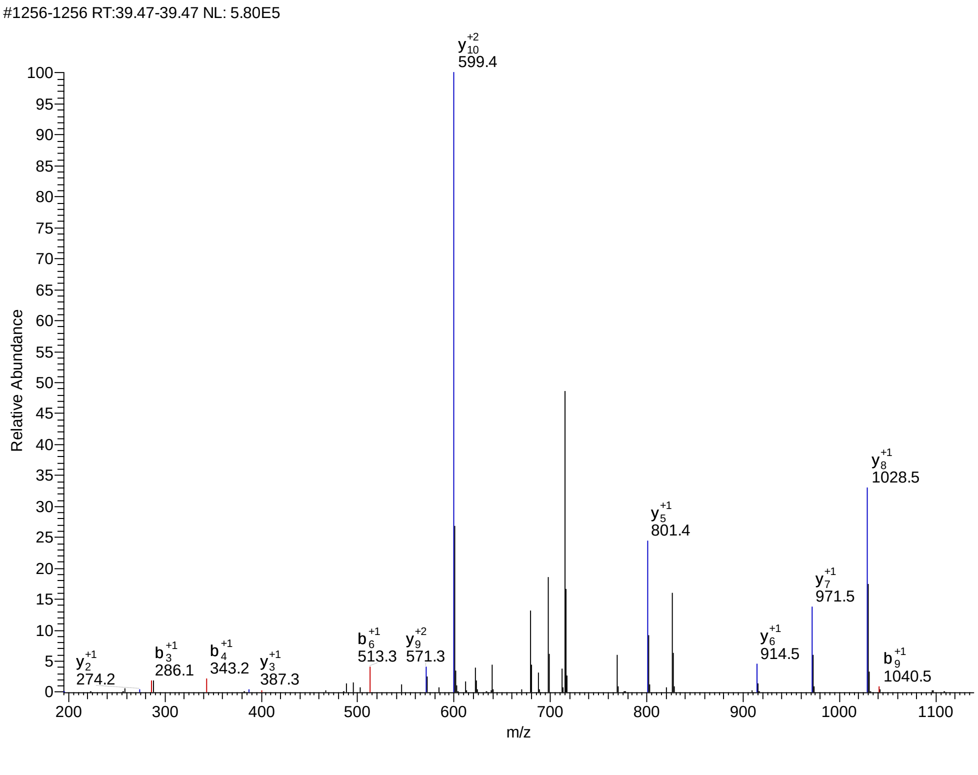

Supplementary Figure 56 – Labelled USP5 peptide D136-R146: CID MS/MS spectra of (labeled) USP5 tryptic peptides with assignments from BioWorks 3 (ThermoFisher) database searching used to confirm peptide identity.

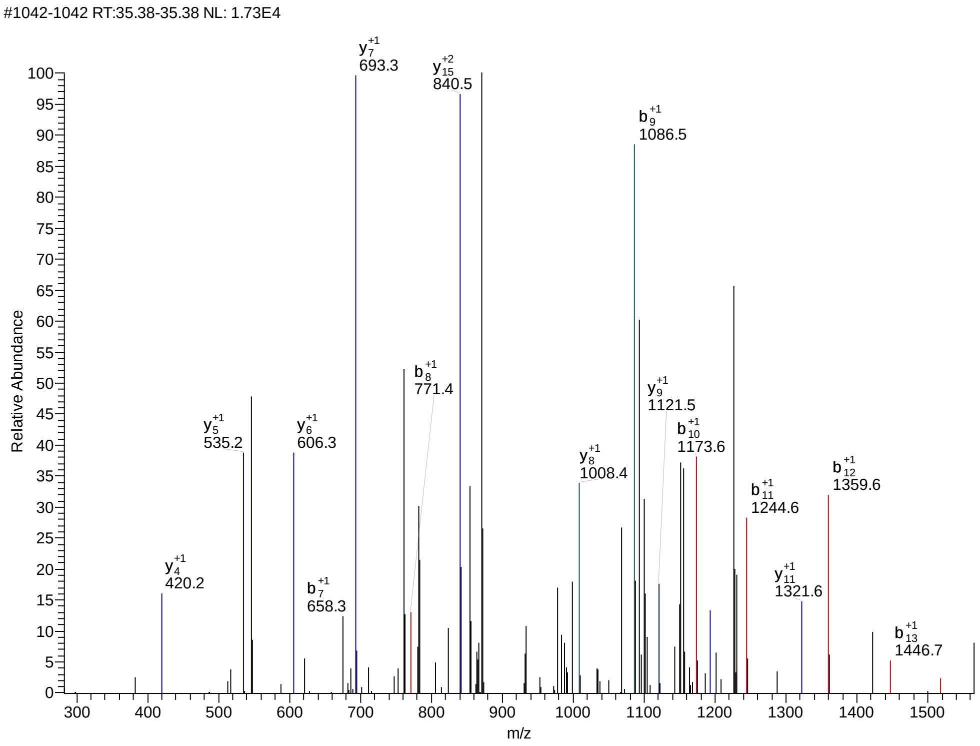

Supplementary Figure 57 – Labelled USP5 peptide V149-R164: CID MS/MS spectra of (labeled) USP5 tryptic peptides with assignments from BioWorks 3 (ThermoFisher) database searching used to confirm peptide identity.

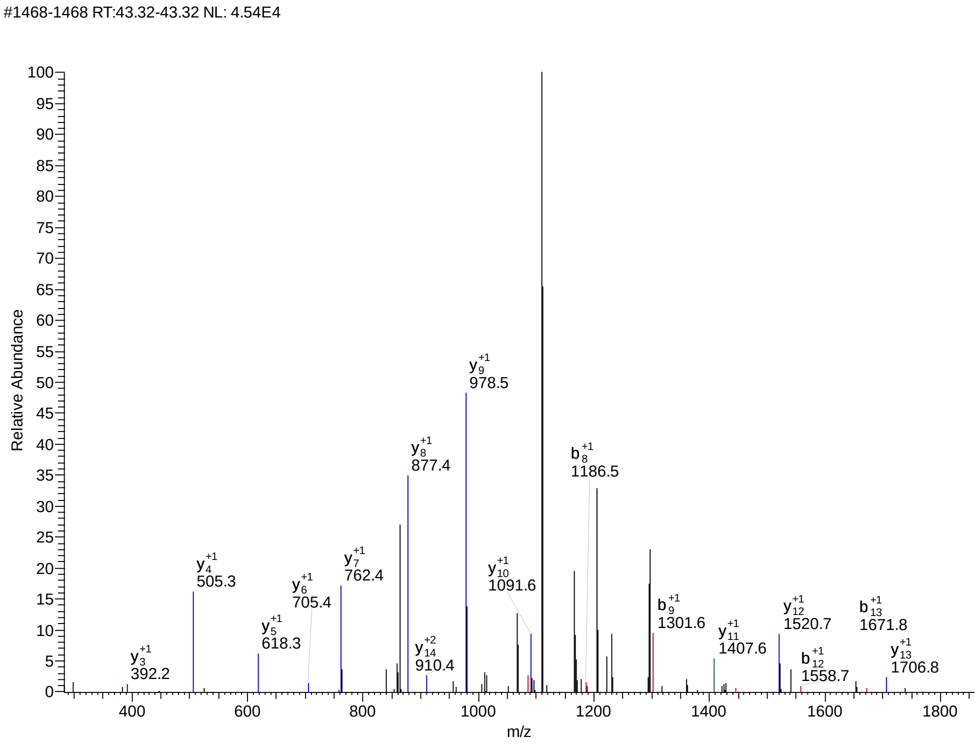

Supplementary Figure 58 – Labelled USP5 peptide E208-R223: CID MS/MS spectra of (labeled) USP5 tryptic peptides with assignments from BioWorks 3 (ThermoFisher) database searching used to confirm peptide identity.

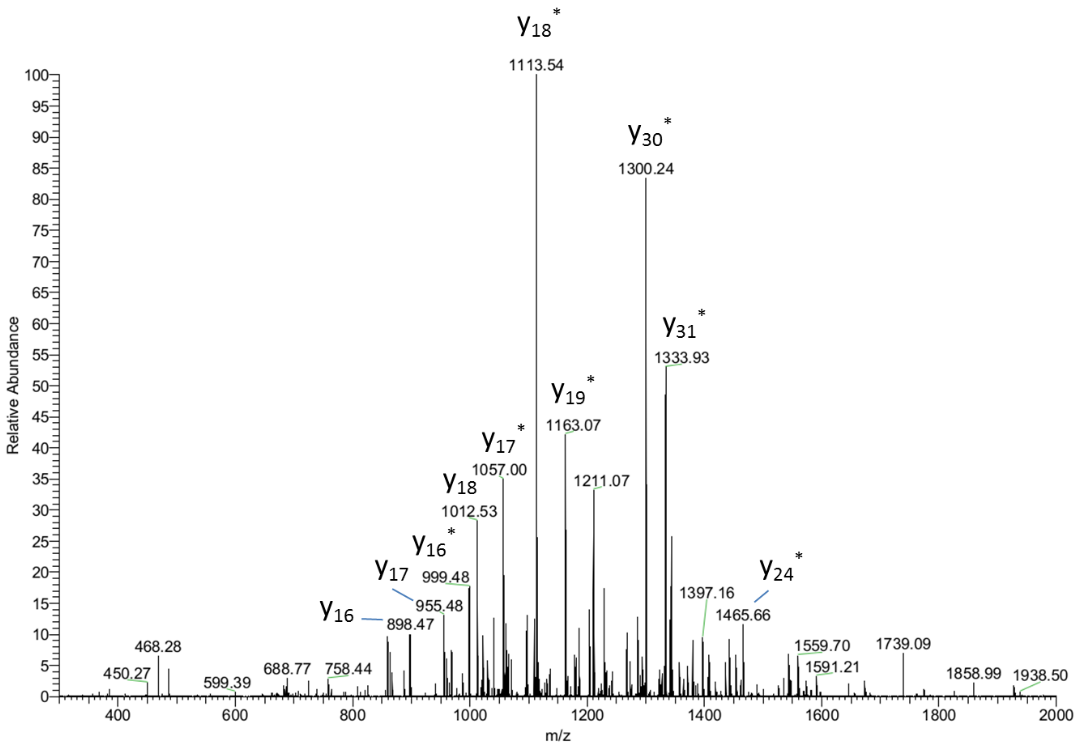

Supplementary Figure 59 – Labelled USP5 peptide L250-K287: CID MS/MS spectra of (labeled) USP5 tryptic peptides with assignments from BioWorks 3 (ThermoFisher) database searching used to confirm peptide identity.

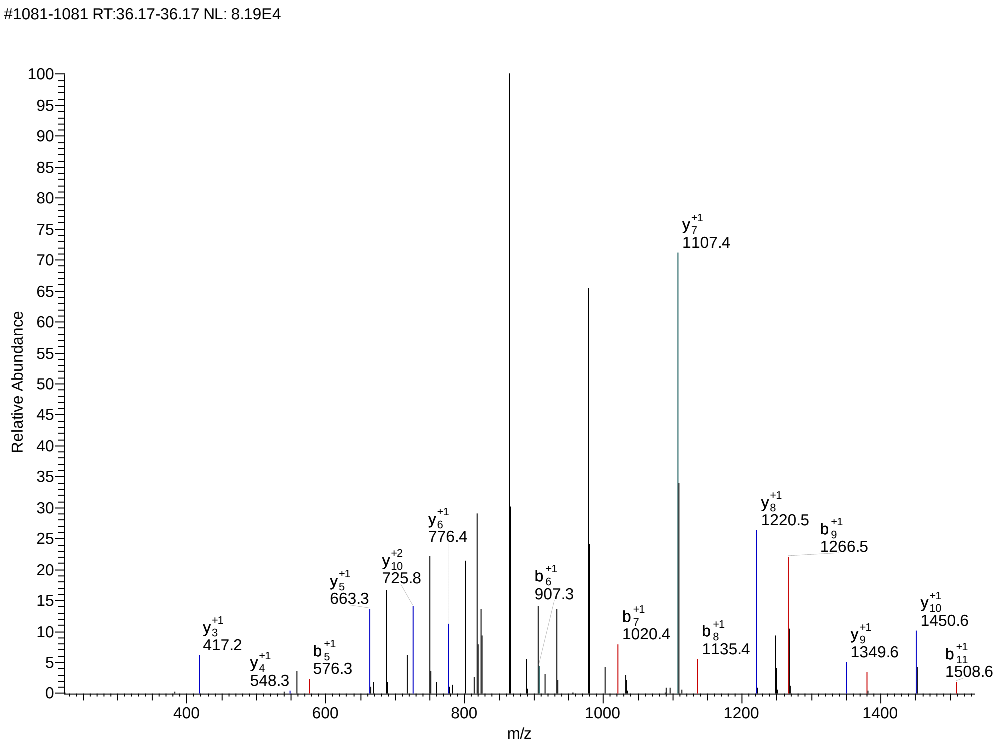

Supplementary Figure 60 – Labelled USP5 peptide T294-R305: CID MS/MS spectra of (labeled) USP5 tryptic peptides with assignments from BioWorks 3 (ThermoFisher) database searching used to confirm peptide identity.

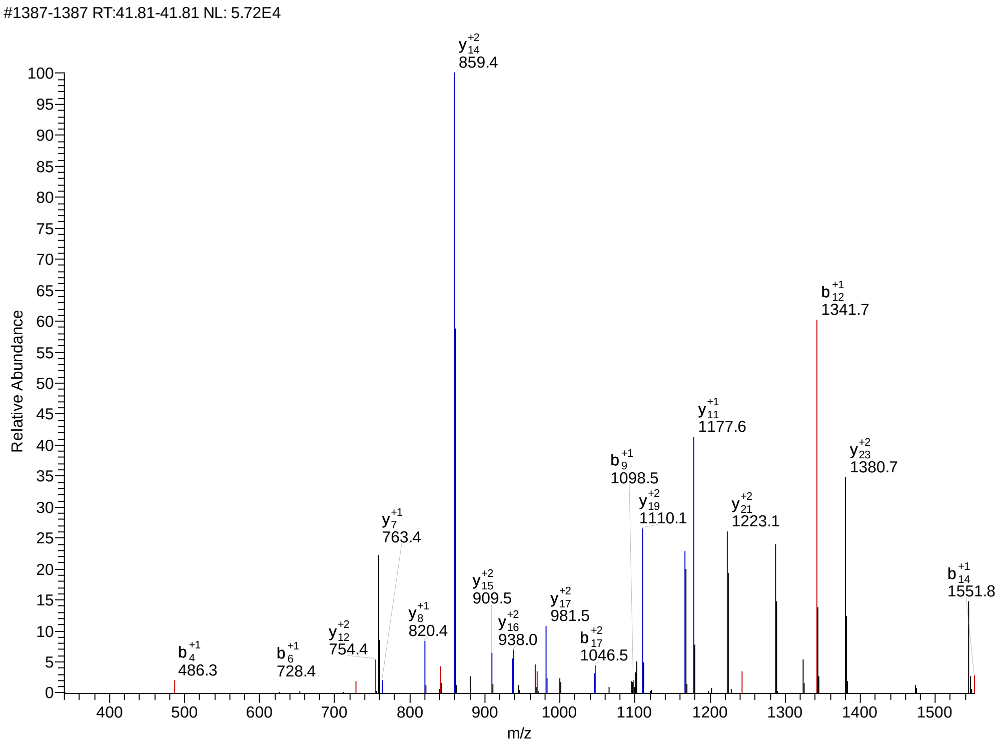

Supplementary Figure 61 – Labelled USP5 peptide I306-R331: CID MS/MS spectra of (labeled) USP5 tryptic peptides with assignments from BioWorks 3 (ThermoFisher) database searching used to confirm peptide identity.

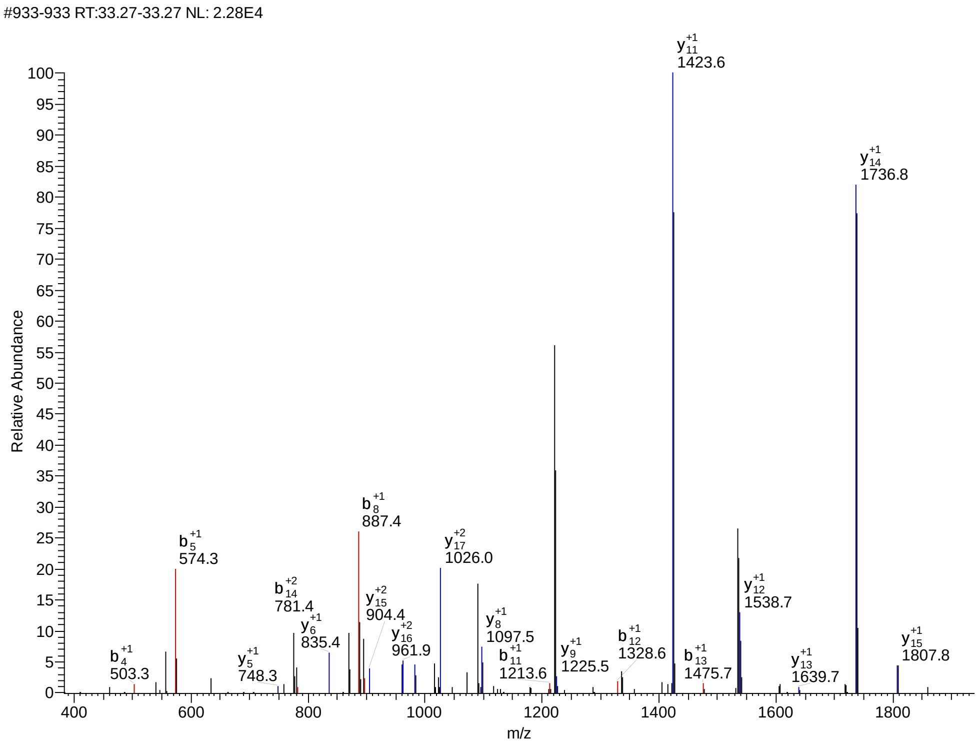

Supplementary Figure 62 – Labelled USP5 peptide I363-K381: CID MS/MS spectra of (labeled) USP5 tryptic peptides with assignments from BioWorks 3 (ThermoFisher) database searching used to confirm peptide identity.

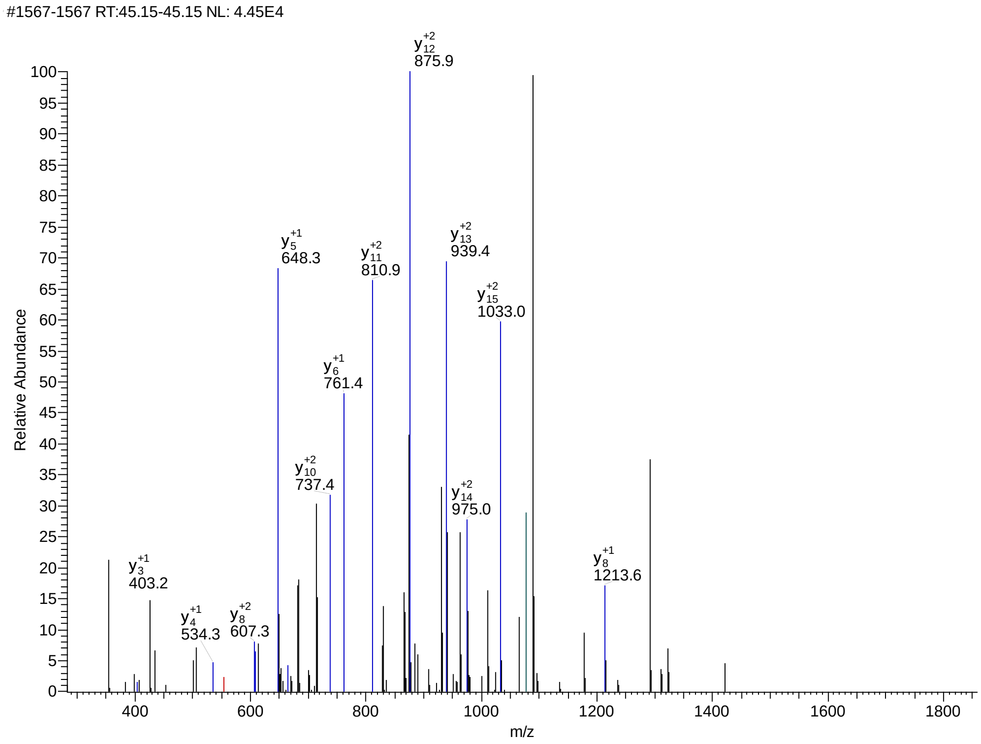

Supplementary Figure 63 – Labelled USP5 peptide Q435-R451: CID MS/MS spectra of (labeled) USP5 tryptic peptides with assignments from BioWorks 3 (ThermoFisher) database searching used to confirm peptide identity.

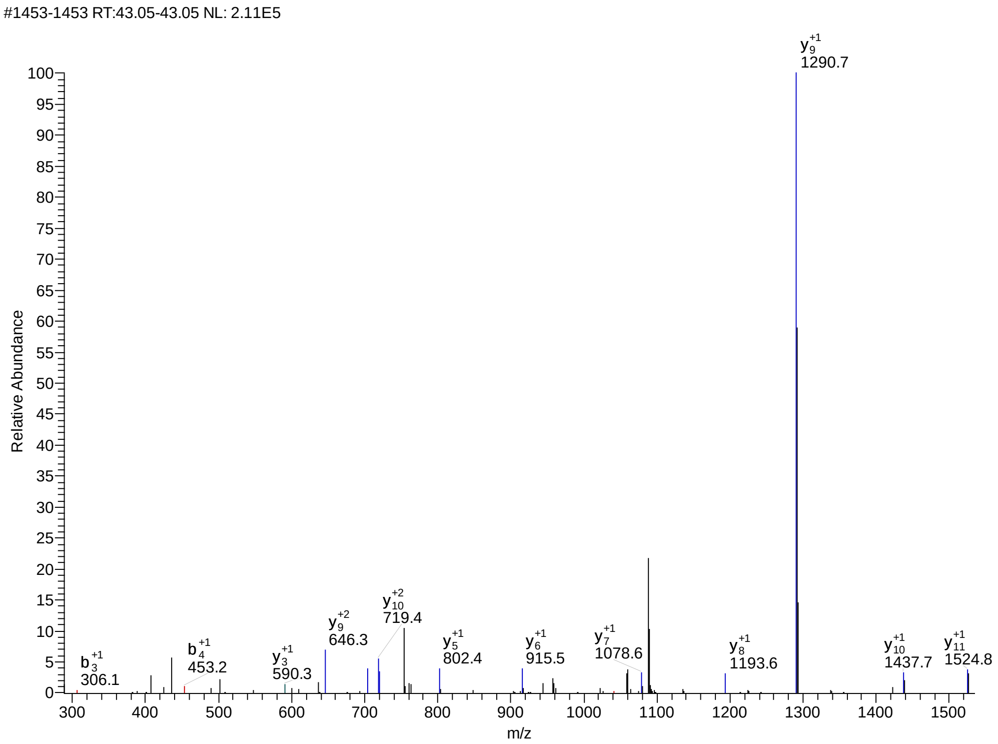

Supplementary Figure 64 – Labelled USP5 peptide F564-K576: CID MS/MS spectra of (labeled) USP5 tryptic peptides with assignments from BioWorks 3 (ThermoFisher) database searching used to confirm peptide identity.

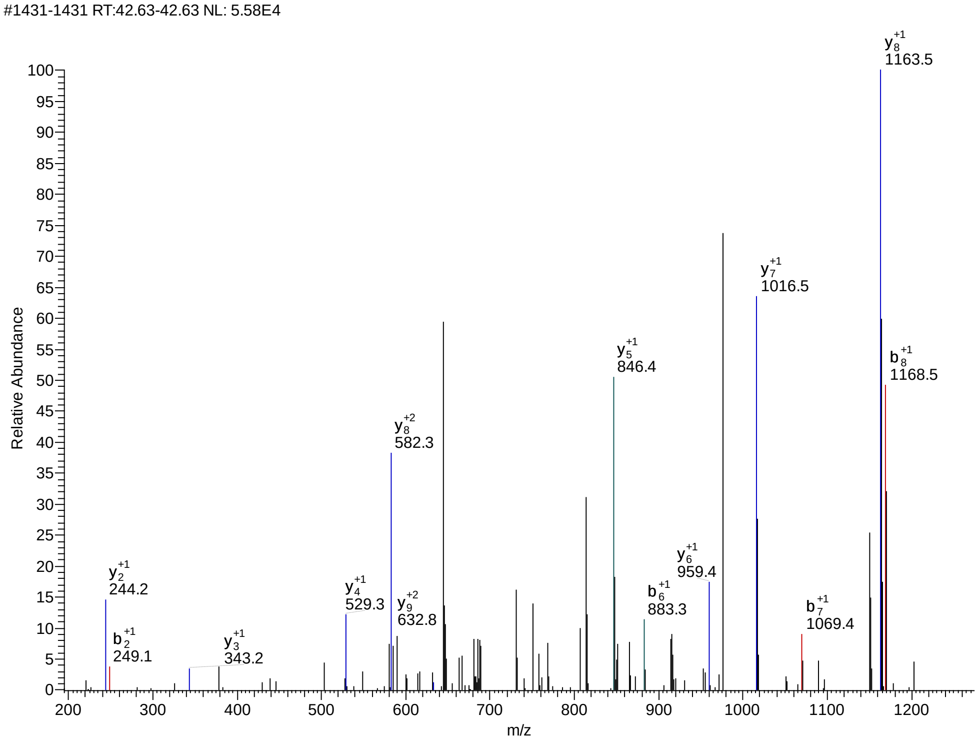

Supplementary Figure 65 – Labelled USP5 peptide F578-K587: CID MS/MS spectra of (labeled) USP5 tryptic peptides with assignments from BioWorks 3 (ThermoFisher) database searching used to confirm peptide identity.

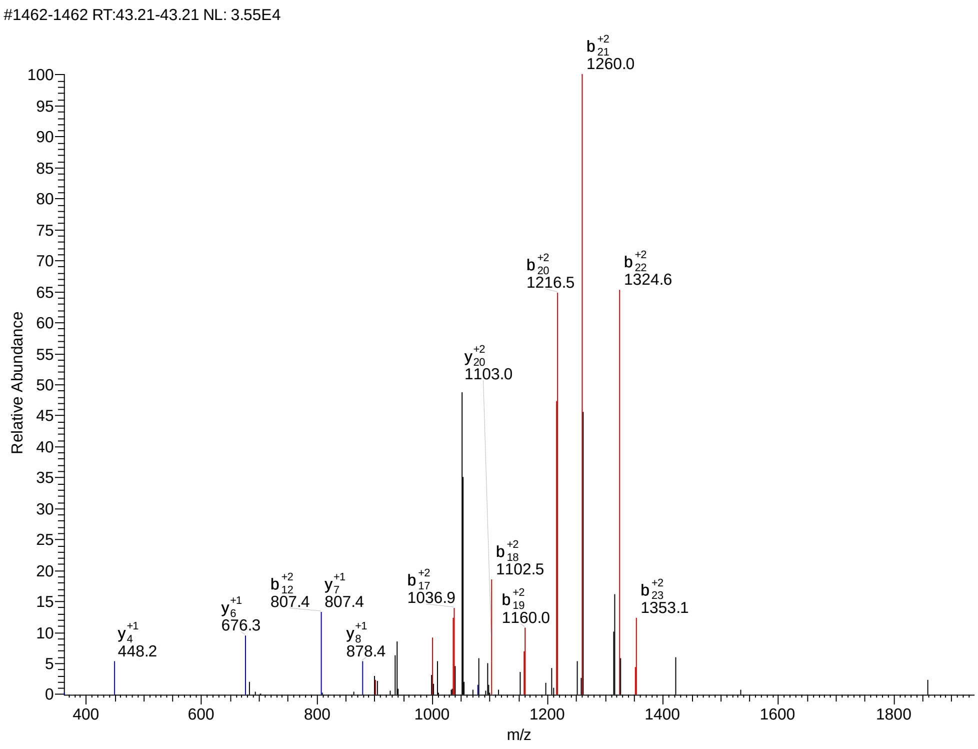

Supplementary Figure 66 – Labelled USP5 peptide A734-R757: CID MS/MS spectra of (labeled) USP5 tryptic peptides with assignments from BioWorks 3 (ThermoFisher) database searching used to confirm peptide identity.

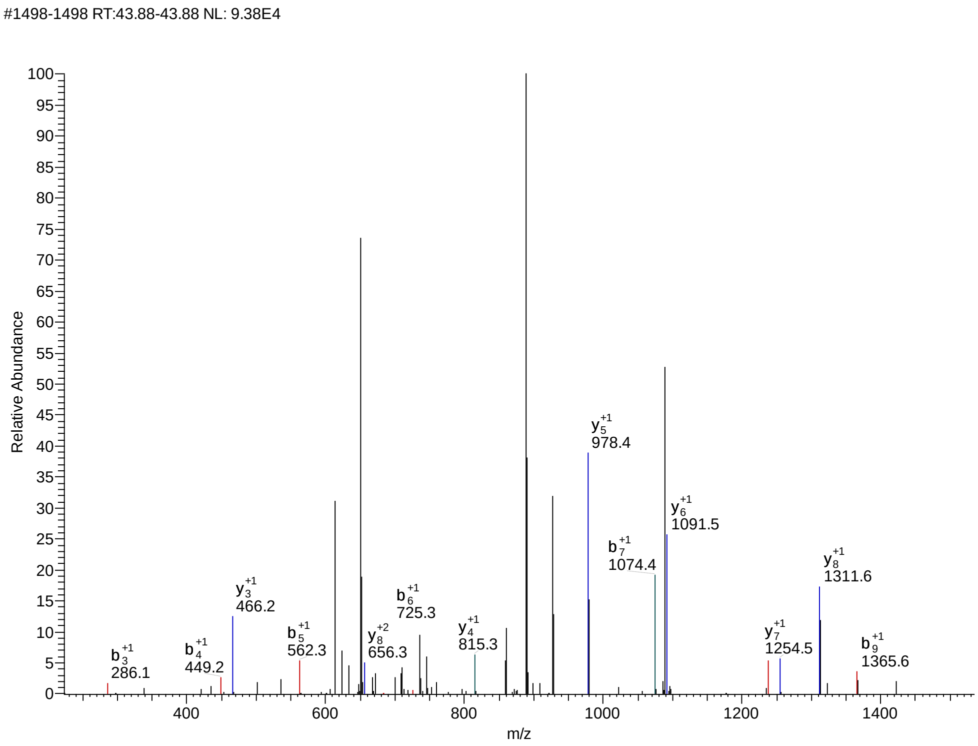

Supplementary Figure 67 – Labelled USP5 peptide D825-R834: CID MS/MS spectra of (labeled) USP5 tryptic peptides with assignments from BioWorks 3 (ThermoFisher) database searching used to confirm peptide identity.

A)

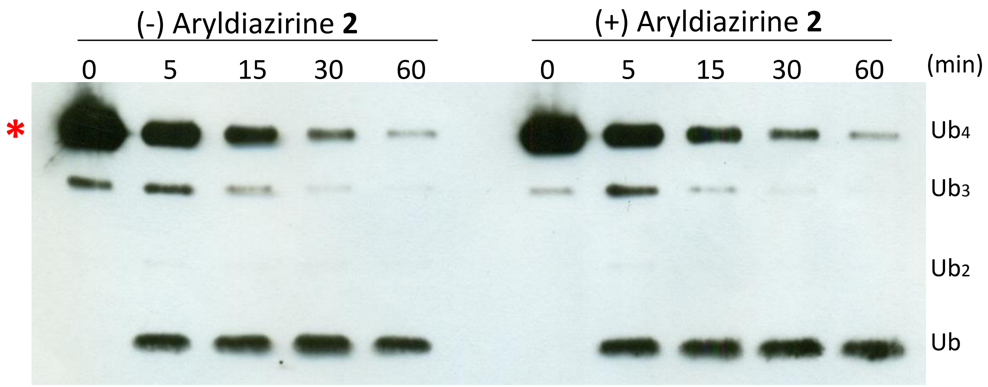

B)

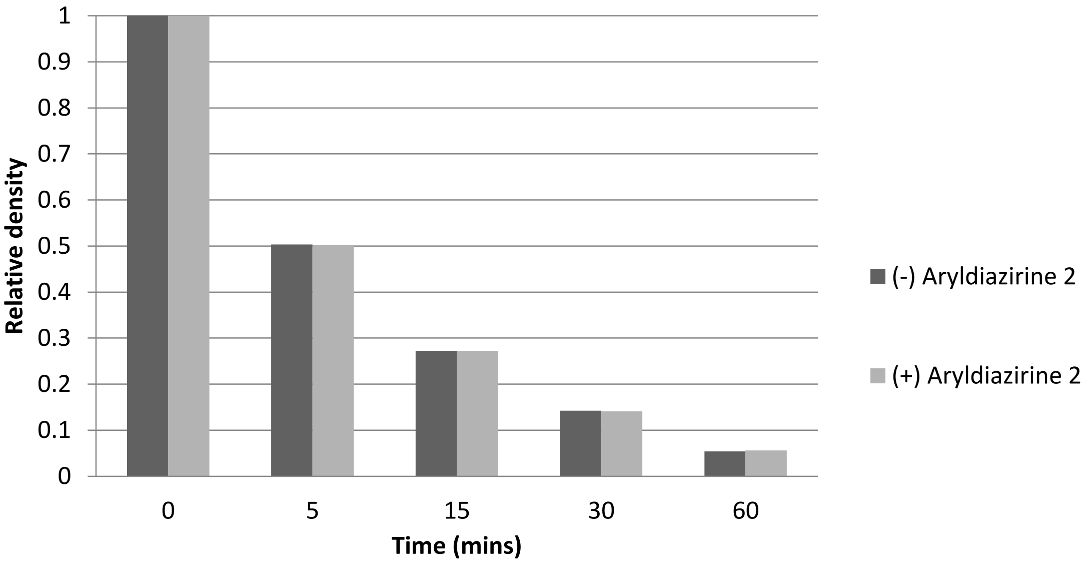

Supplementary Figure 68 - Investigating USP5 catalytic activity in the presence of aryldiazirine 2. A) Western blot of tetraubiquitin deubiquitination by WT USP5, in the presence and absence of aryldiazirine 2. B) Densitometric analysis of tetraubiquitin deubiquitination (band marked with * in A) over the time course.

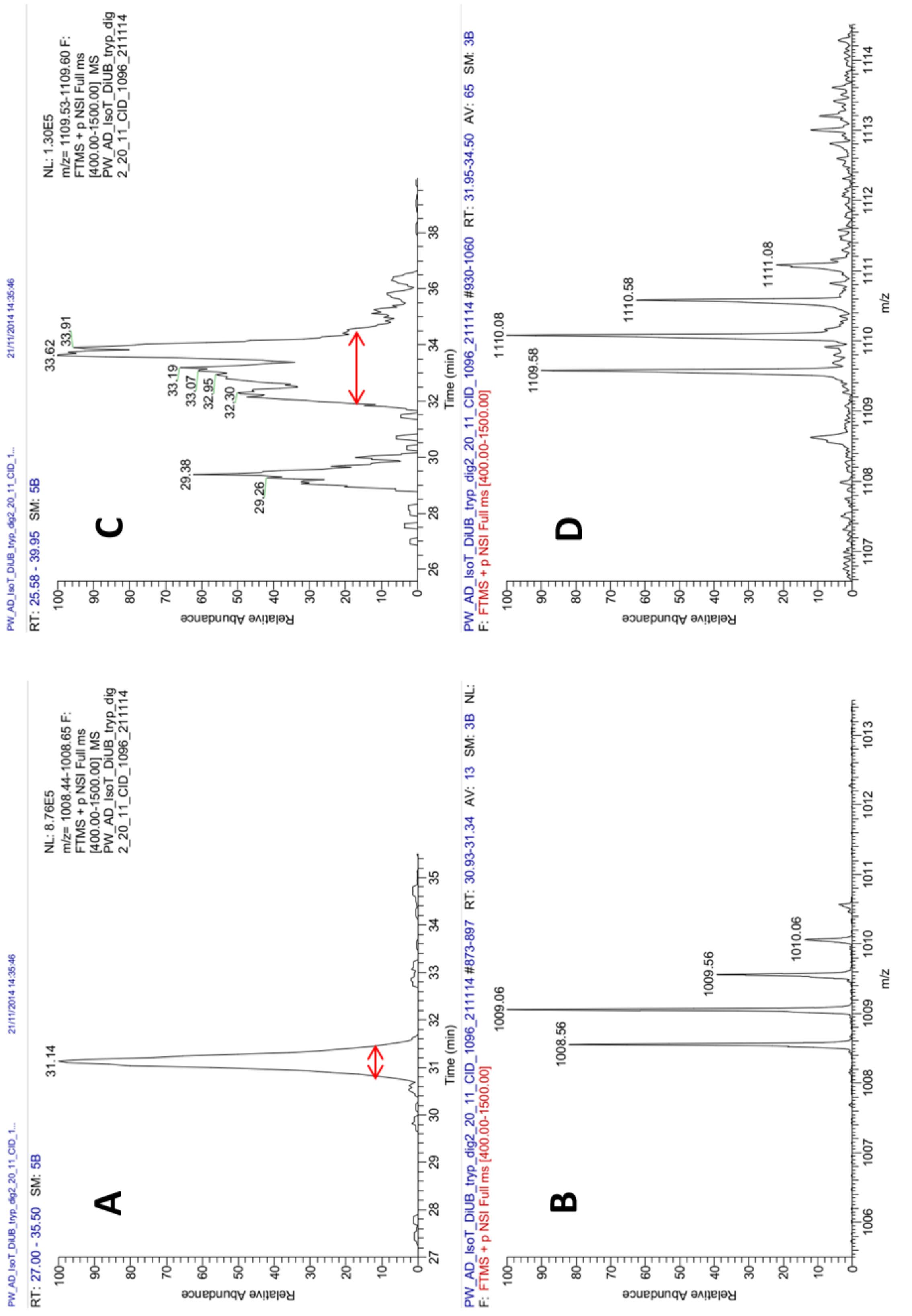

Supplementary Figure 69 – G1-R19: example of the extracted ion chromatogram (XIC) for the unlabeled and labeled precursors (A and C, respectively), and the spectra of the unlabeled and labeled precursor ions (B and D, respectively). The red arrow indicates approximately the area over which the data has been averaged.

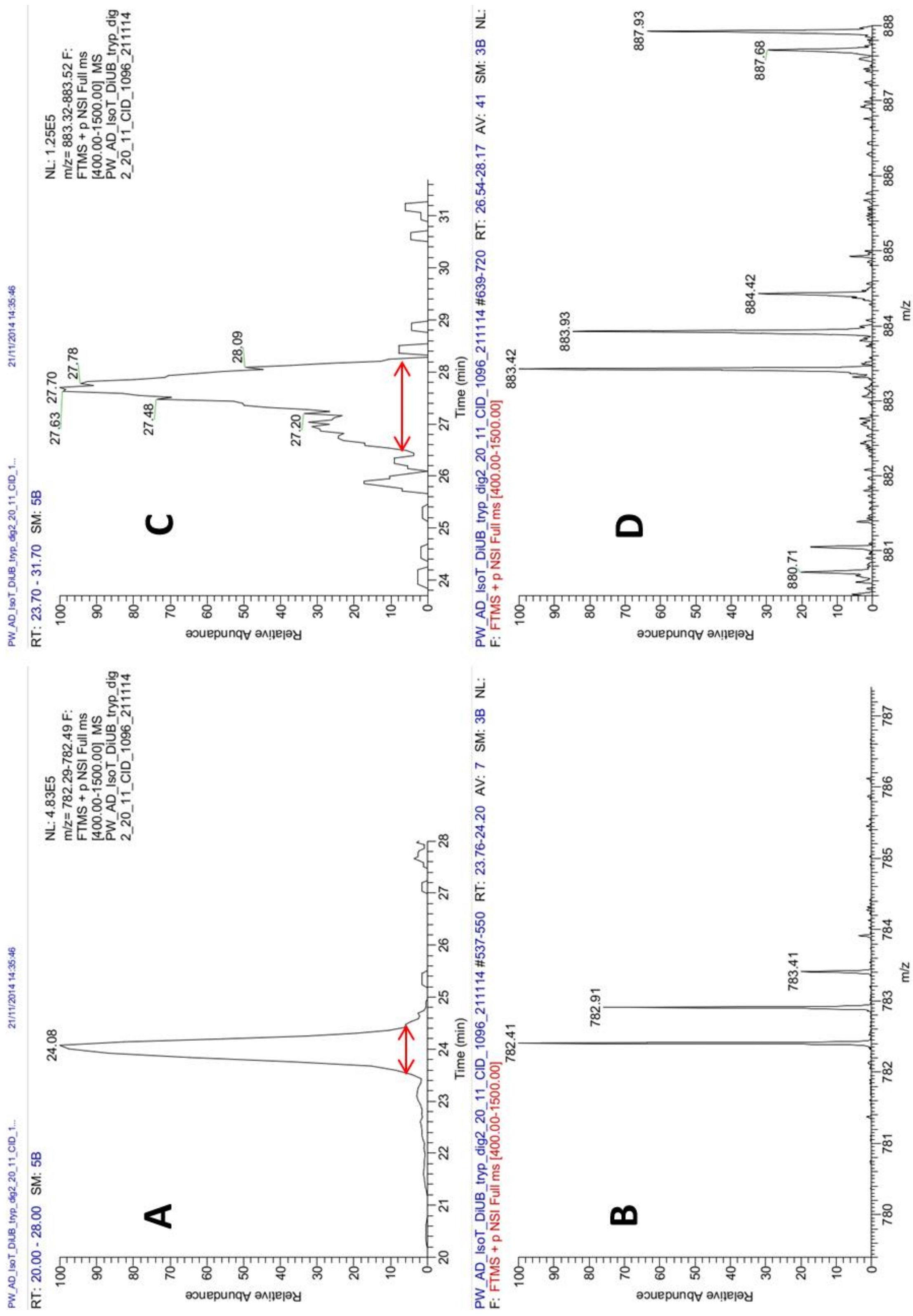

Supplementary Figure 70 – L101-K115: example of the extracted ion chromatogram (XIC) for the unlabeled and labeled precursors (A and C, respectively), and the spectra of the unlabeled and labeled precursor ions (B and D, respectively). The red arrow indicates approximately the area over which the data has been averaged.

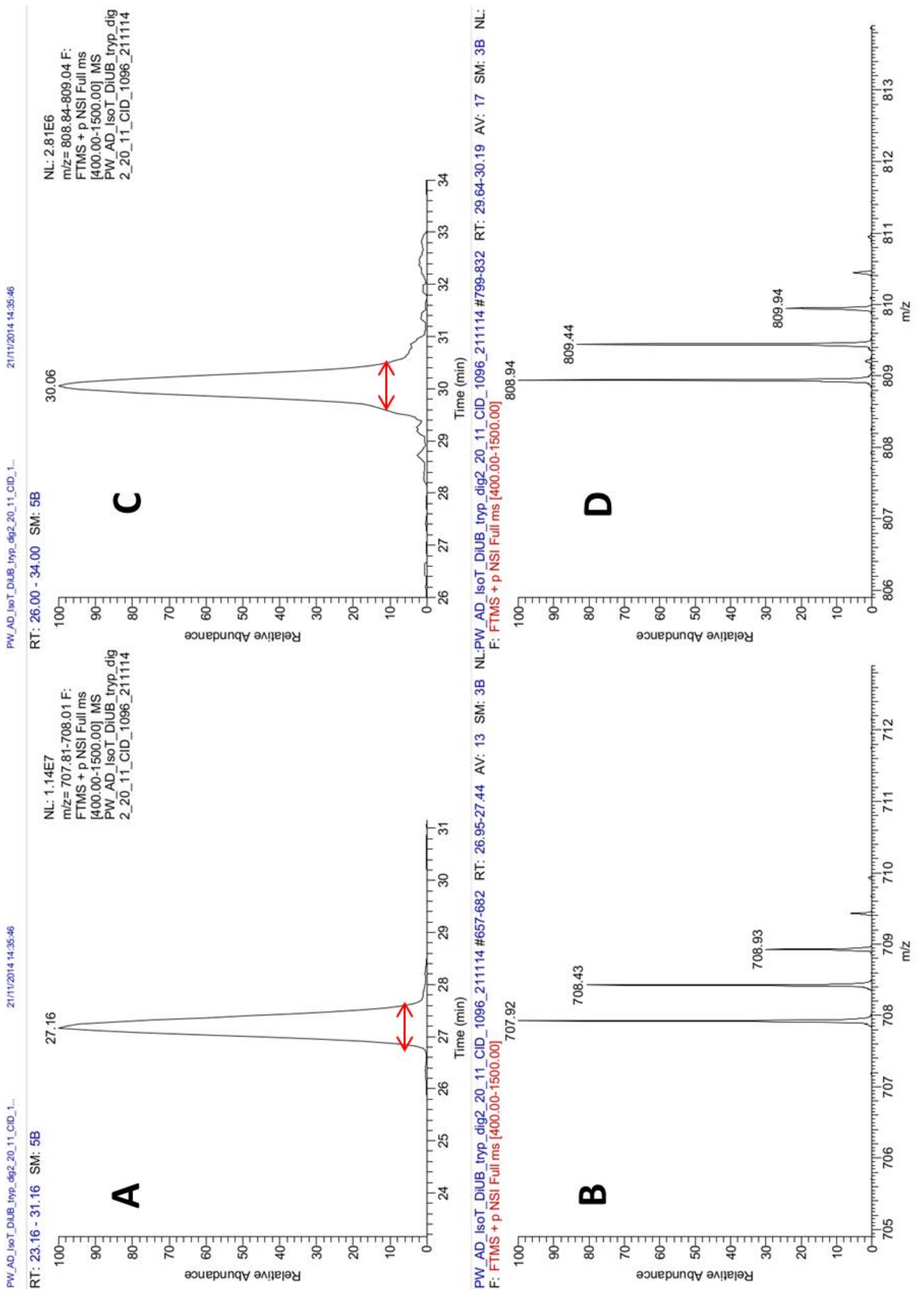

Supplementary Figure 71– I124-R135: example of the extracted ion chromatogram (XIC) for the unlabeled and labeled precursors (A and C, respectively), and the spectra of the unlabeled and labeled precursor ions (B and D, respectively). The red arrow indicates approximately the area over which the data has been averaged.

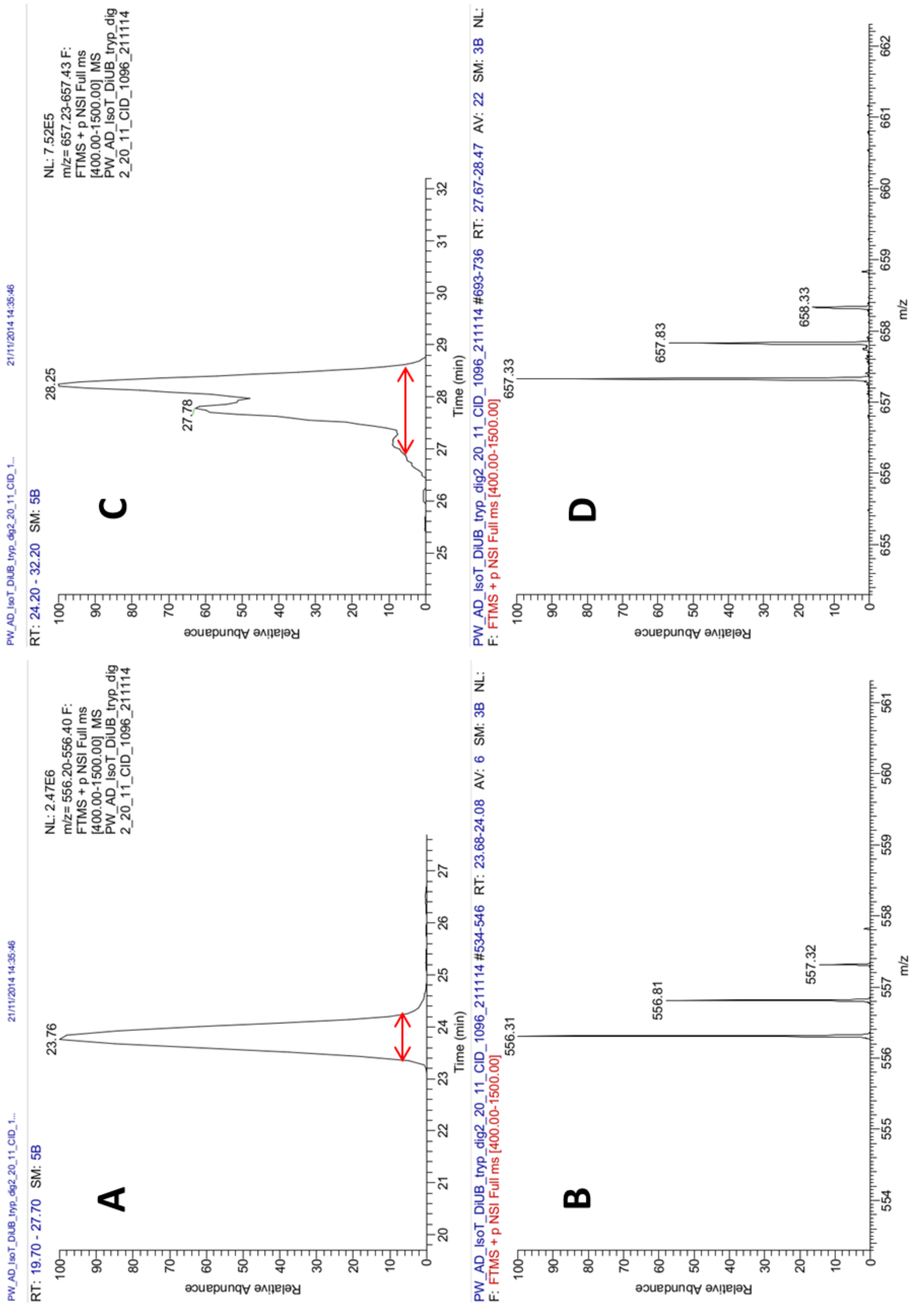

Supplementary Figure 72 – D136-R146: example of the extracted ion chromatogram (XIC) for the unlabeled and labeled precursors (A and C, respectively), and the spectra of the unlabeled and labeled precursor ions (B and D, respectively). The red arrow indicates approximately the area over which the data has been averaged.

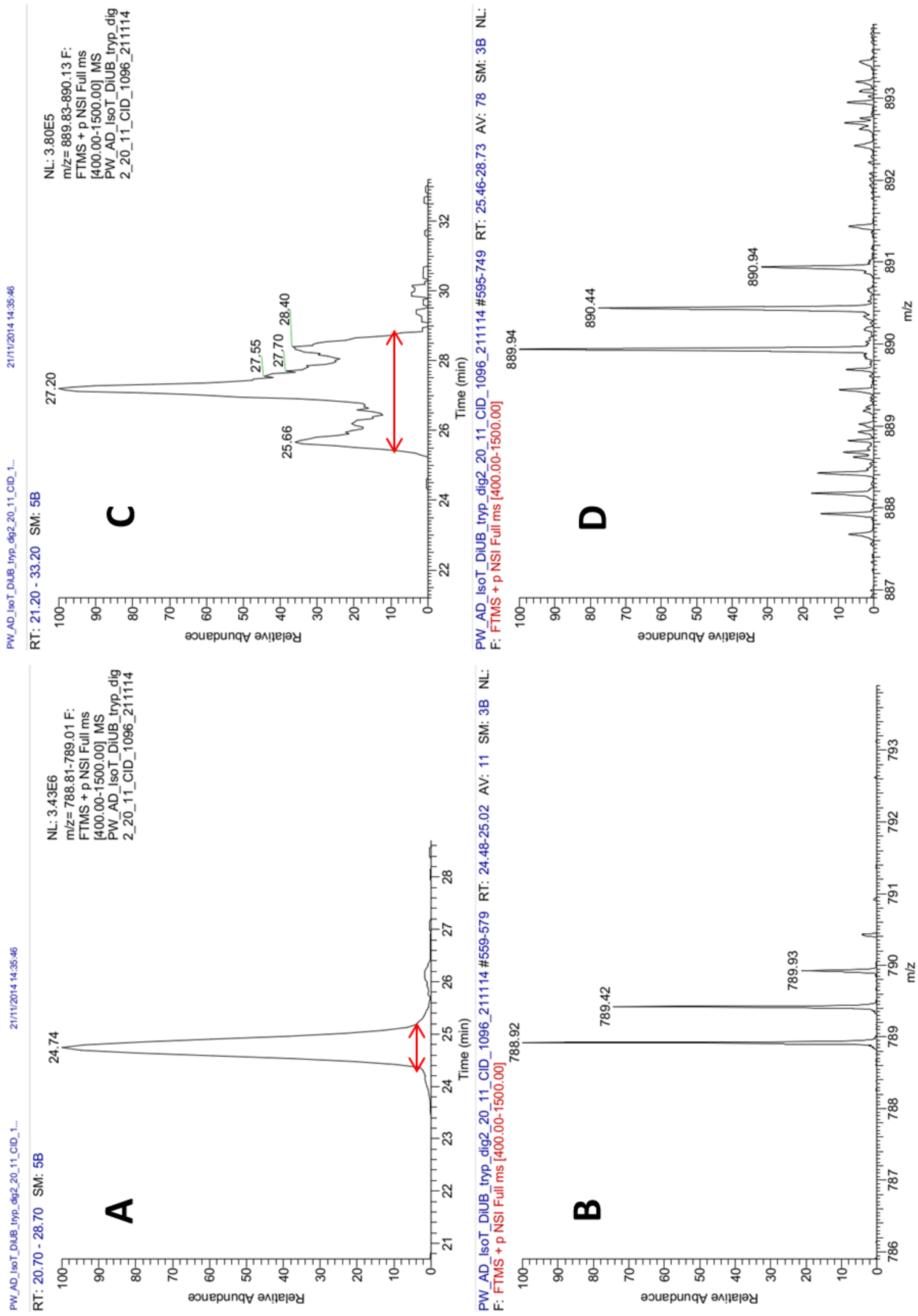

Supplementary Figure 73 – V149-R164: example of the extracted ion chromatogram (XIC) for the unlabeled and labeled precursors (A and C, respectively), and the spectra of the unlabeled and labeled precursor ions (B and D, respectively). The red arrow indicates approximately the area over which the data has been averaged.

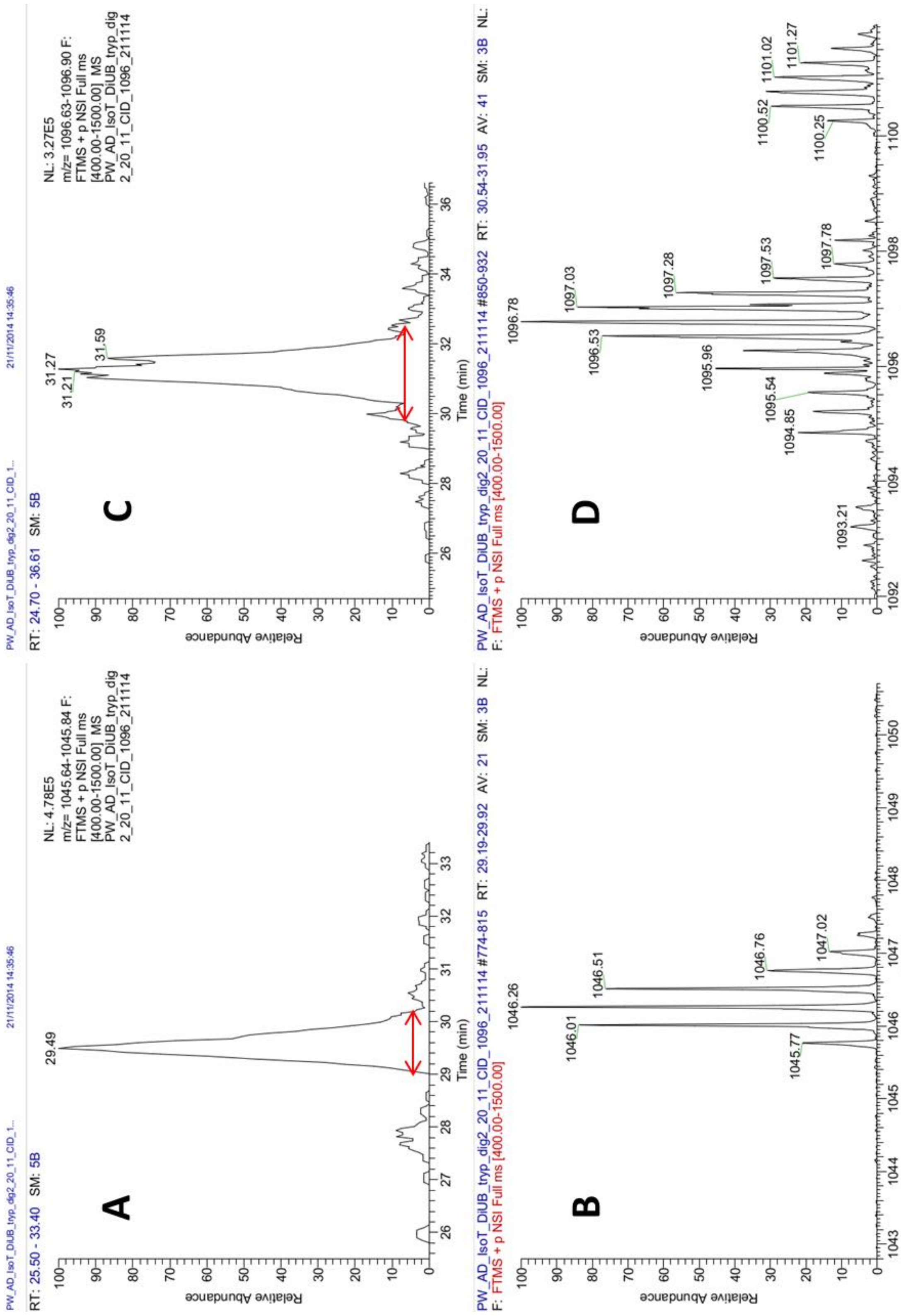

Supplementary Figure 74 – L250-K287: example of the extracted ion chromatogram (XIC) for the unlabeled and labeled precursors (A and C, respectively), and the spectra of the unlabeled and labeled precursor ions (B and D, respectively). The red arrow indicates approximately the area over which the data has been averaged.

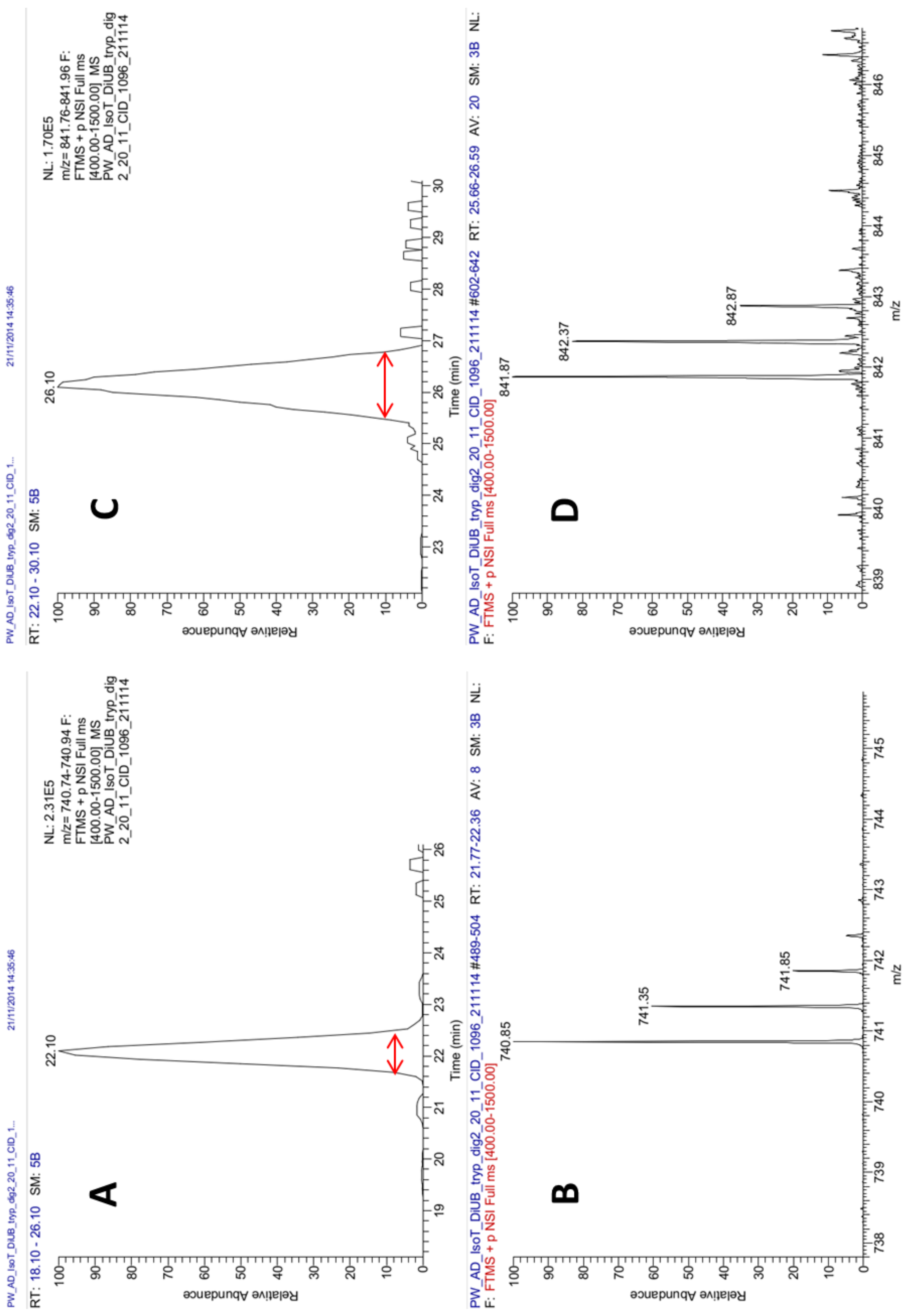

Supplementary Figure 75 – T294-R305: example of the extracted ion chromatogram (XIC) for the unlabeled and labeled precursors (A and C, respectively), and the spectra of the unlabeled and labeled precursor ions (B and D, respectively). The red arrow indicates approximately the area over which the data has been averaged.

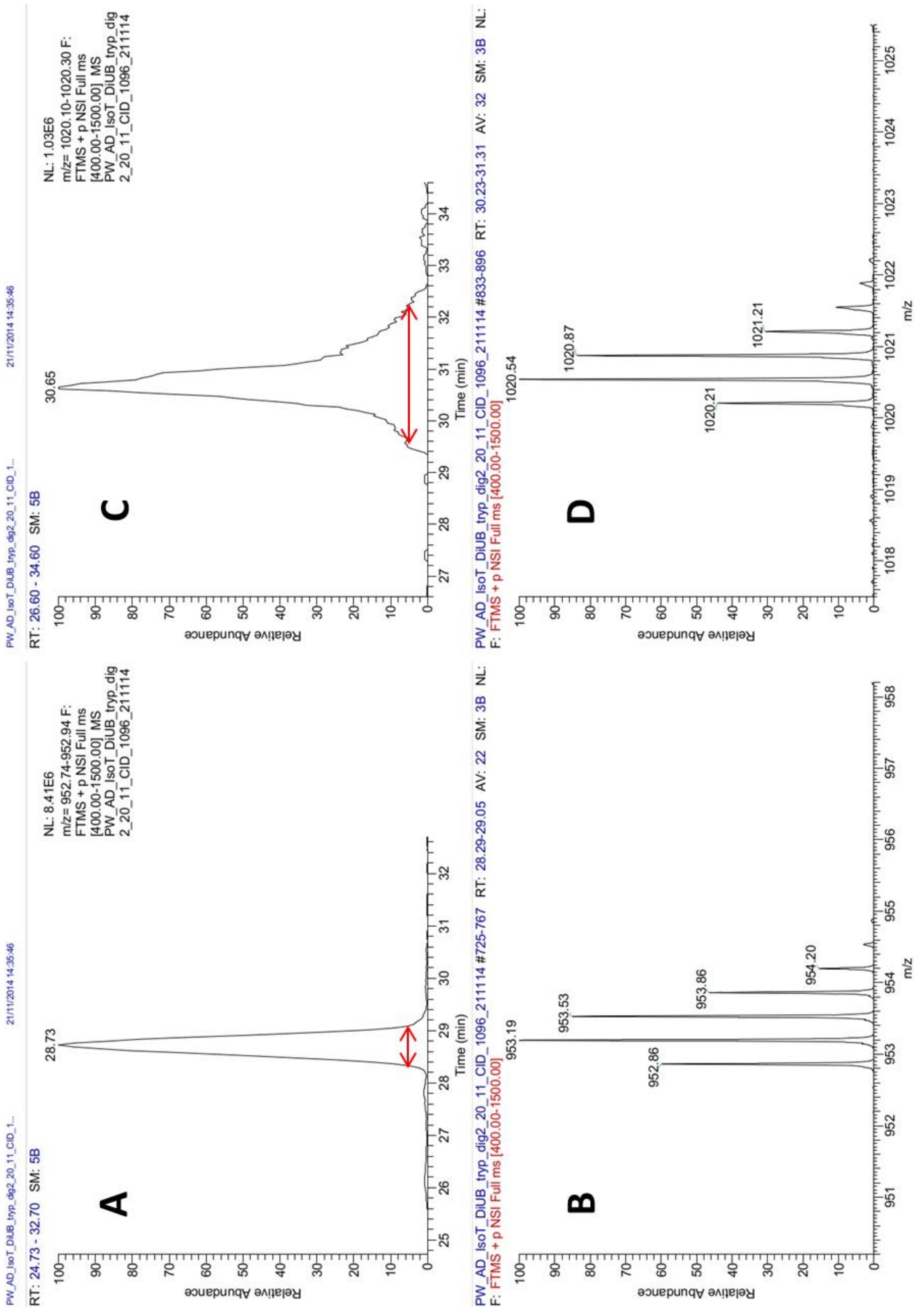

Supplementary Figure 76 – I306-R331: example of the extracted ion chromatogram (XIC) for the unlabeled and labeled precursors (A and C, respectively), and the spectra of the unlabeled and labeled precursor ions (B and D, respectively). The red arrow indicates approximately the area over which the data has been averaged.

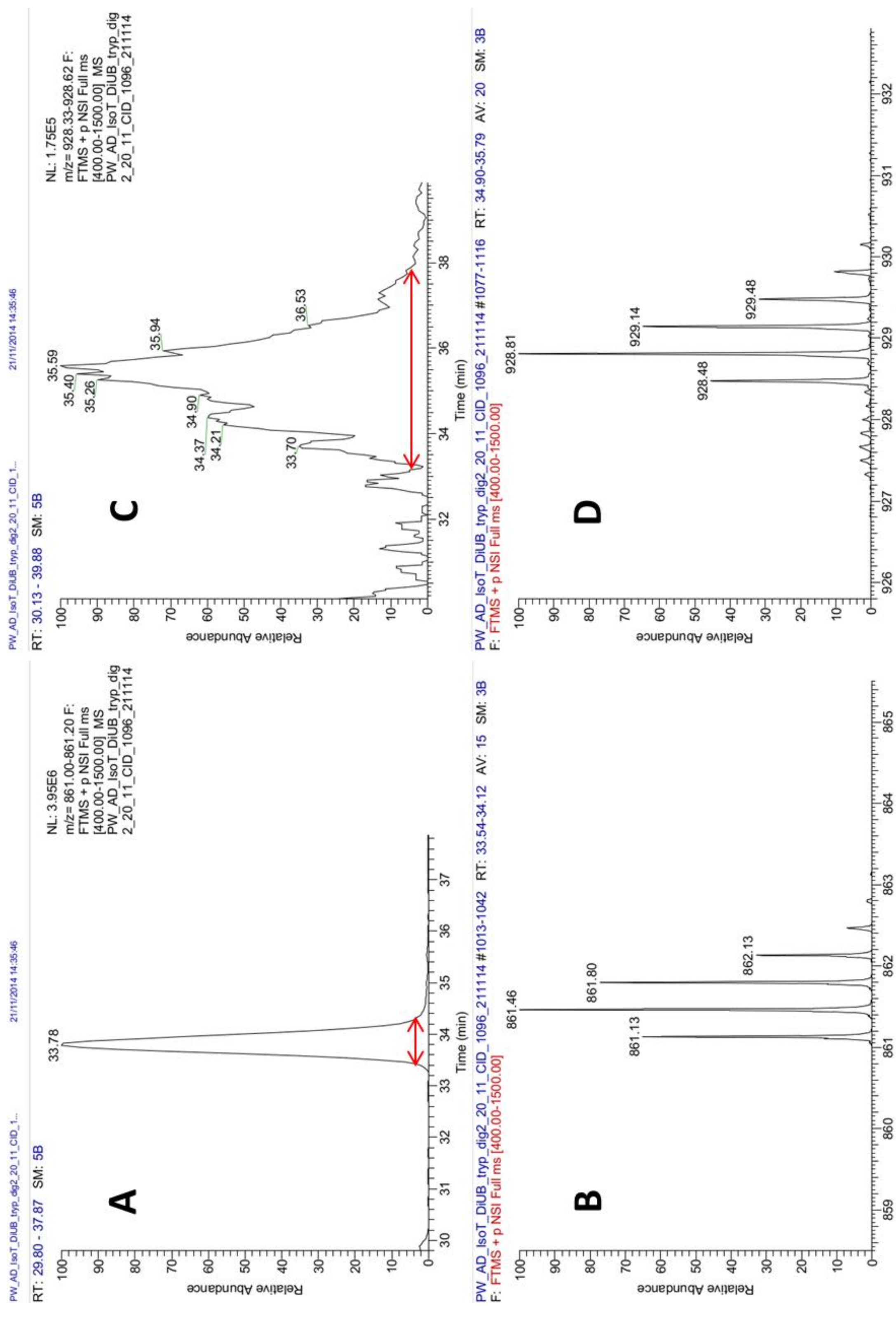

Supplementary Figure 77 – N332-R354: example of the extracted ion chromatogram (XIC) for the unlabeled and labeled precursors (A and C, respectively), and the spectra of the unlabeled and labeled precursor ions (B and D, respectively). The red arrow indicates approximately the area over which the data has been averaged.

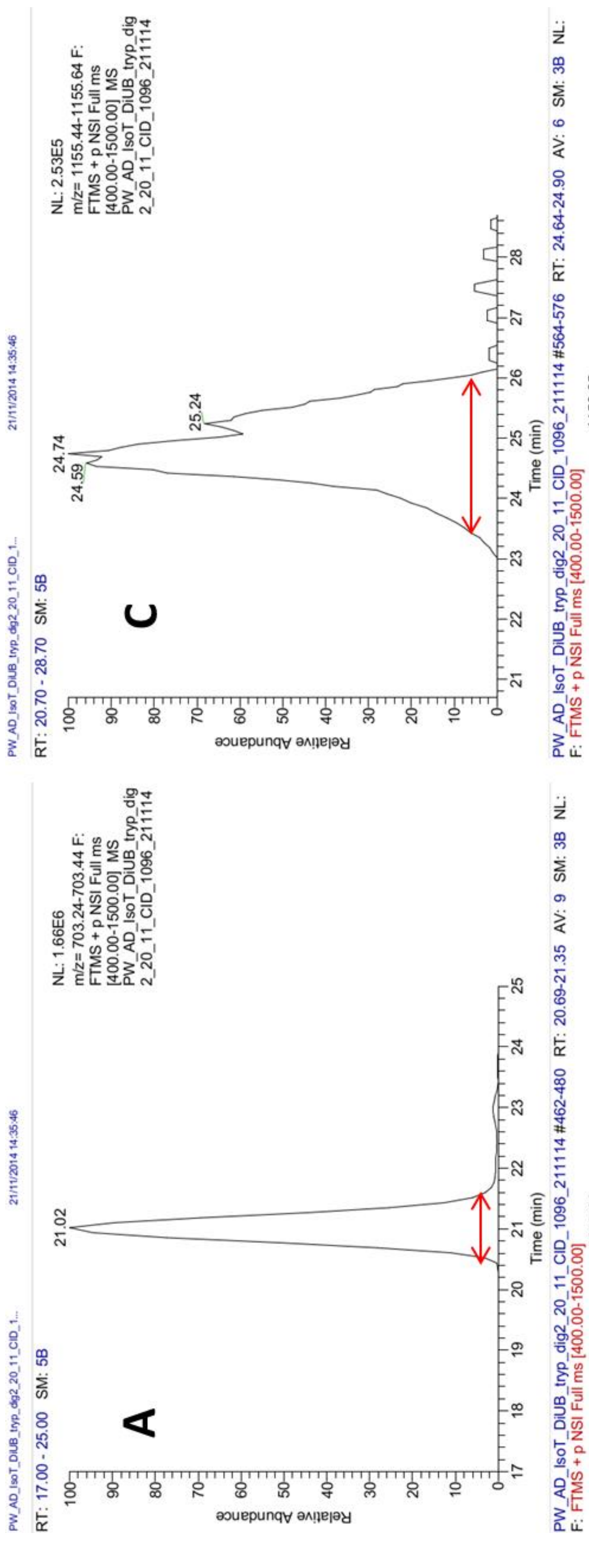

Supplementary Figure 78 – I363-K381: example of the extracted ion chromatogram (XIC) for the unlabeled and labeled precursors (A and C, respectively), and the spectra of the unlabeled and labeled precursor ions (B and D, respectively). The red arrow indicates approximately the area over which the data has been averaged.

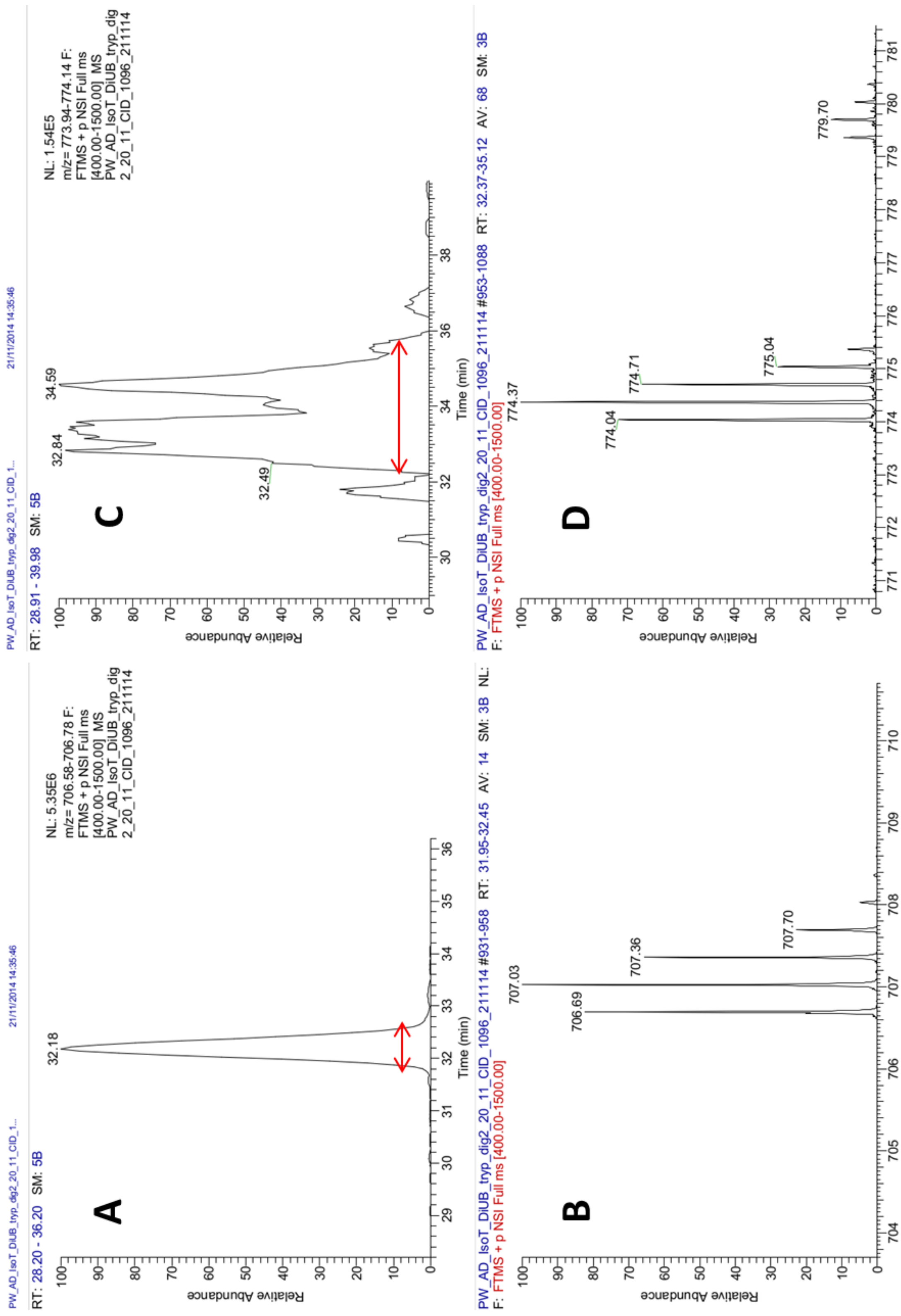

Supplementary Figure 79 – Q435-R451: example of the extracted ion chromatogram (XIC) for the unlabeled and labeled precursors (A and C, respectively), and the spectra of the unlabeled and labeled precursor ions (B and D, respectively). The red arrow indicates approximately the area over which the data has been averaged.

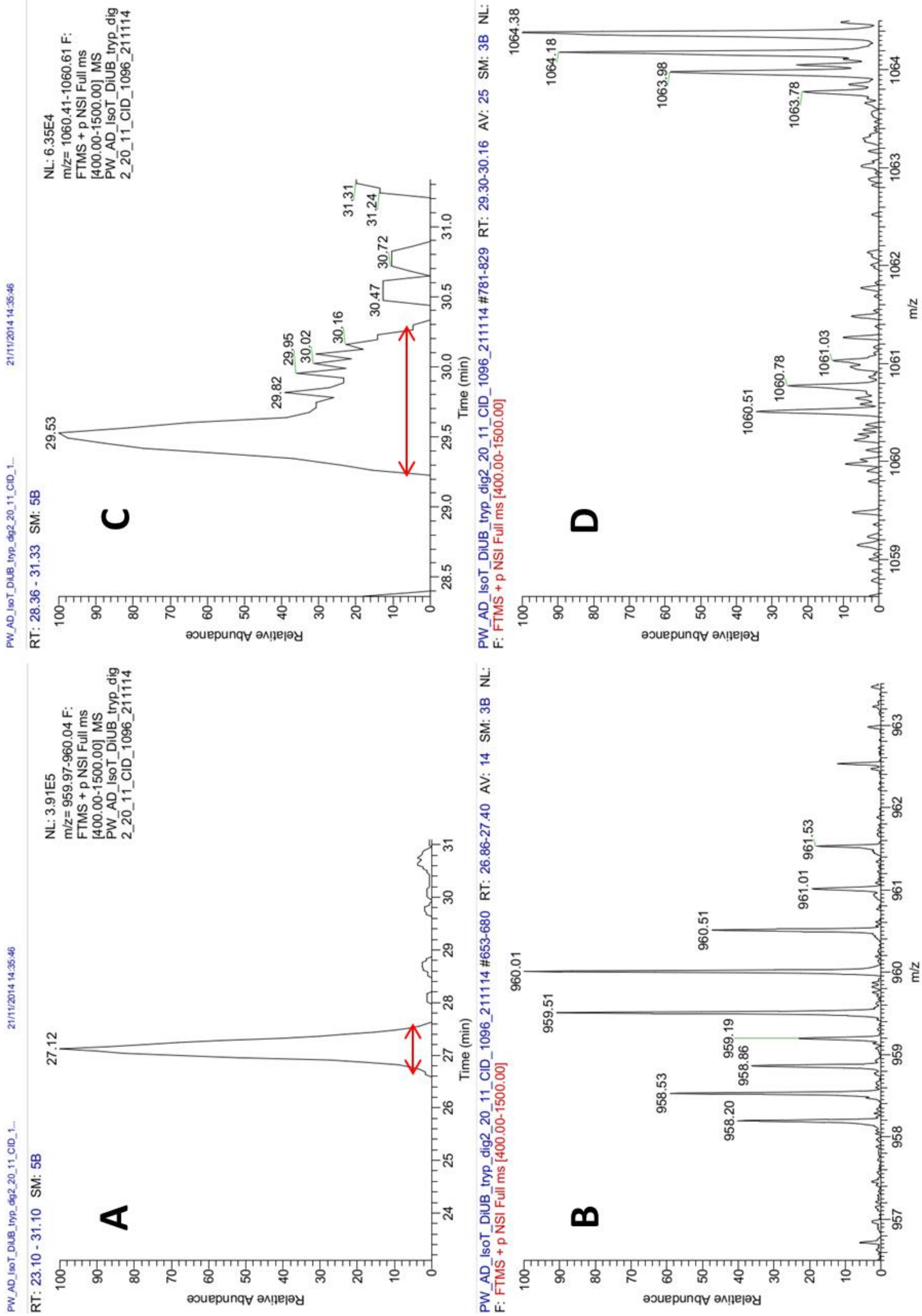

Supplementary Figure 80– V485-K501: example of the extracted ion chromatogram (XIC) for the unlabeled and labeled precursors (A and C, respectively), and the spectra of the unlabeled and labeled precursor ions (B and D, respectively). The red arrow indicates approximately the area over which the data has been averaged.

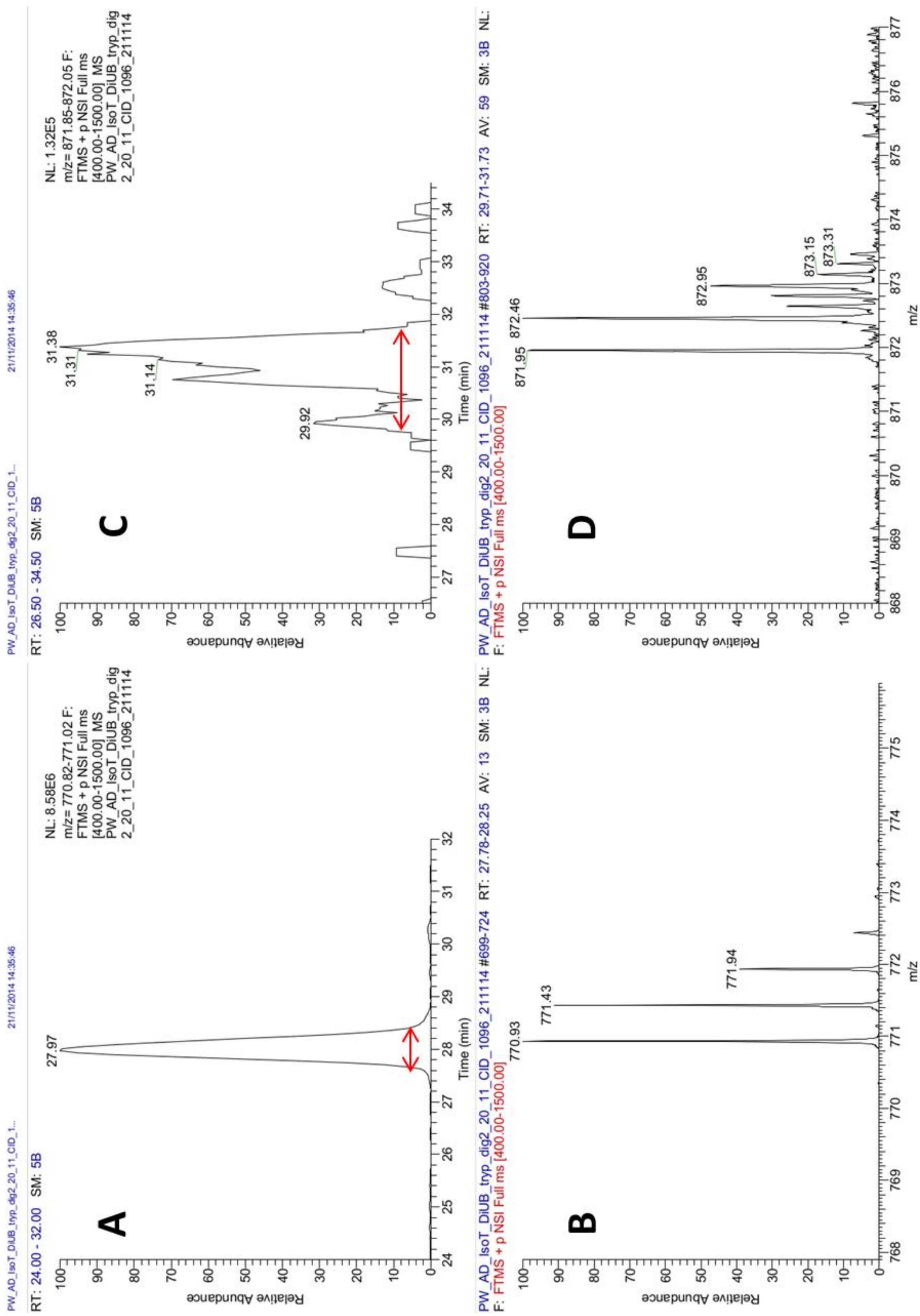

Supplementary Figure 81 – F564-K576: example of the extracted ion chromatogram (XIC) for the unlabeled and labeled precursors (A and C, respectively), and the spectra of the unlabeled and labeled precursor ions (B and D, respectively). The red arrow indicates approximately the area over which the data has been averaged.

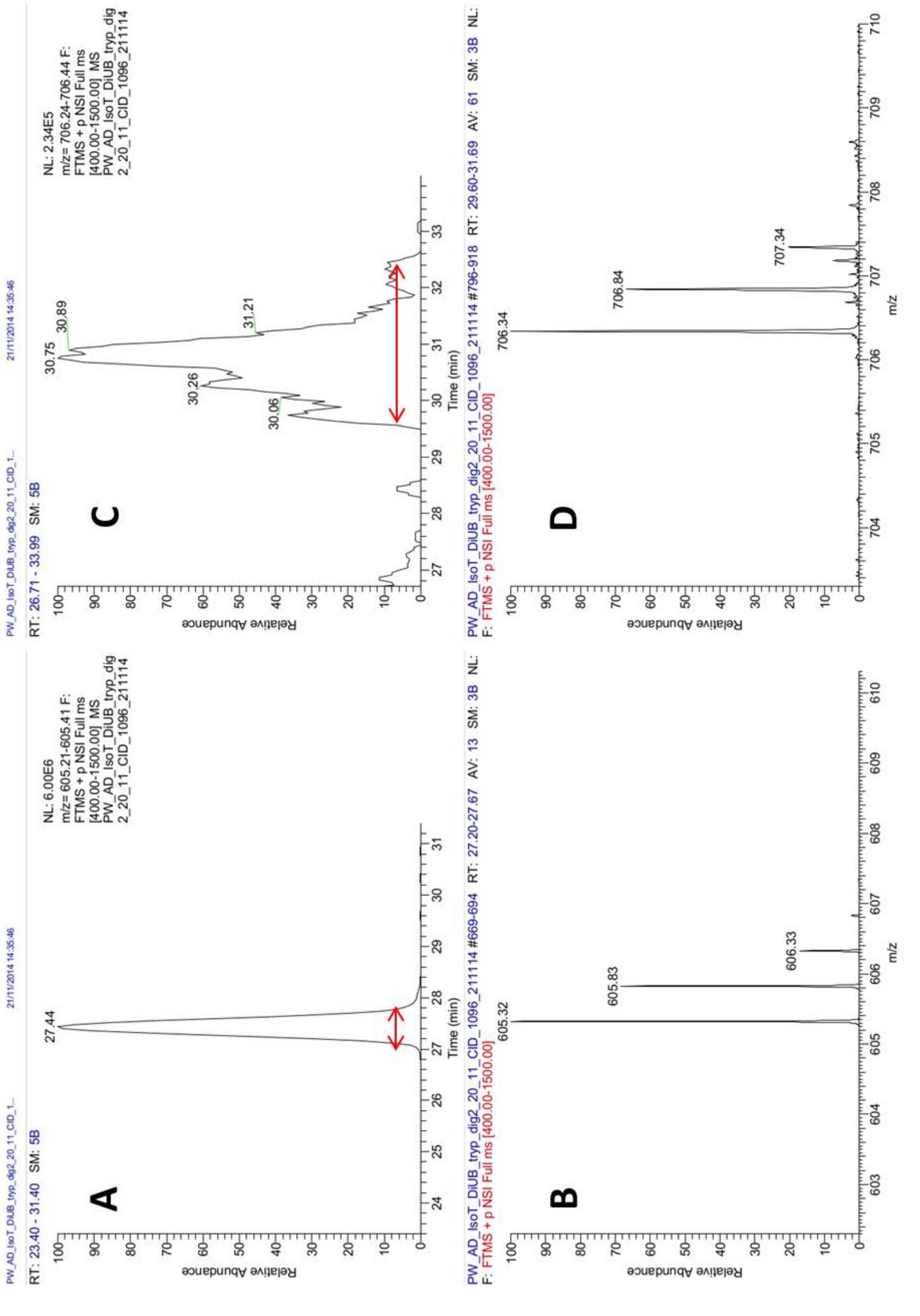

Supplementary Figure 82 – F578-K587: example of the extracted ion chromatogram (XIC) for the unlabeled and labeled precursors (A and C, respectively), and the spectra of the unlabeled and labeled precursor ions (B and D, respectively). The red arrow indicates approximately the area over which the data has been averaged.

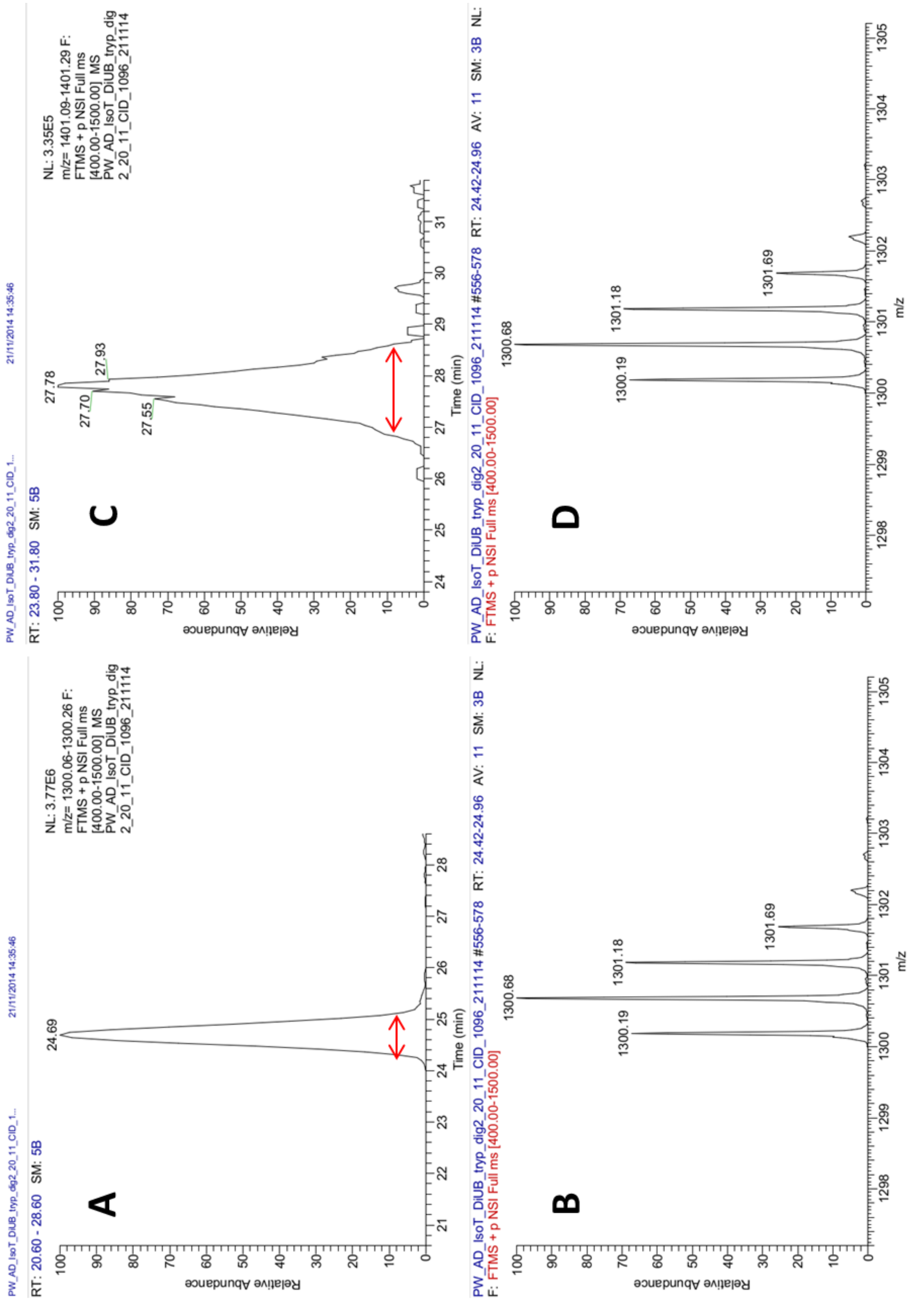

Supplementary Figure 83 – G606-K630: example of the extracted ion chromatogram (XIC) for the unlabeled and labeled precursors (A and C, respectively), and the spectra of the unlabeled and labeled precursor ions (B and D, respectively). The red arrow indicates approximately the area over which the data has been averaged.

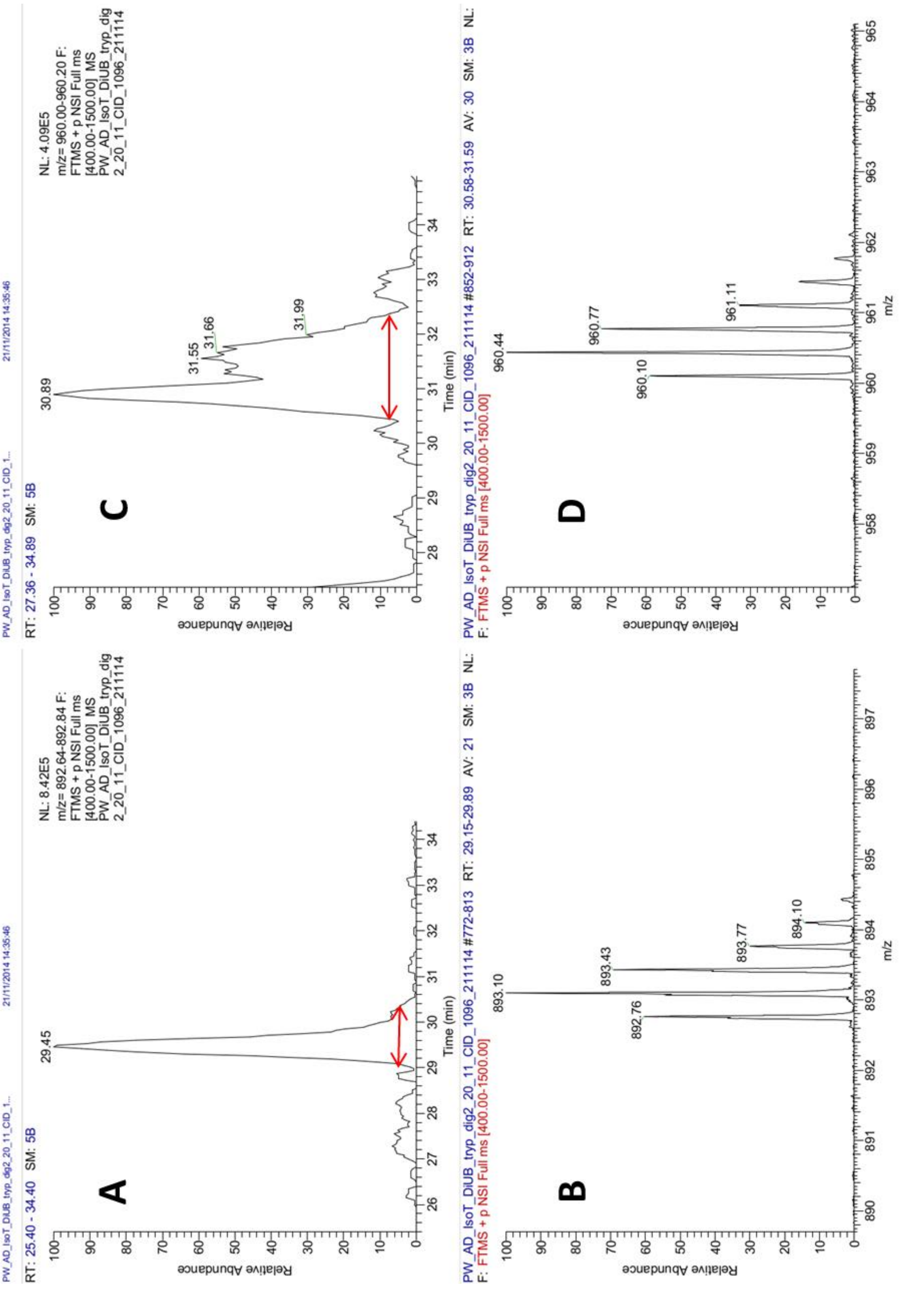

Supplementary Figure 84 – A734-R757: example of the extracted ion chromatogram (XIC) for the unlabeled and labeled precursors (A and C, respectively), and the spectra of the unlabeled and labeled precursor ions (B and D, respectively). The red arrow indicates approximately the area over which the data has been averaged.

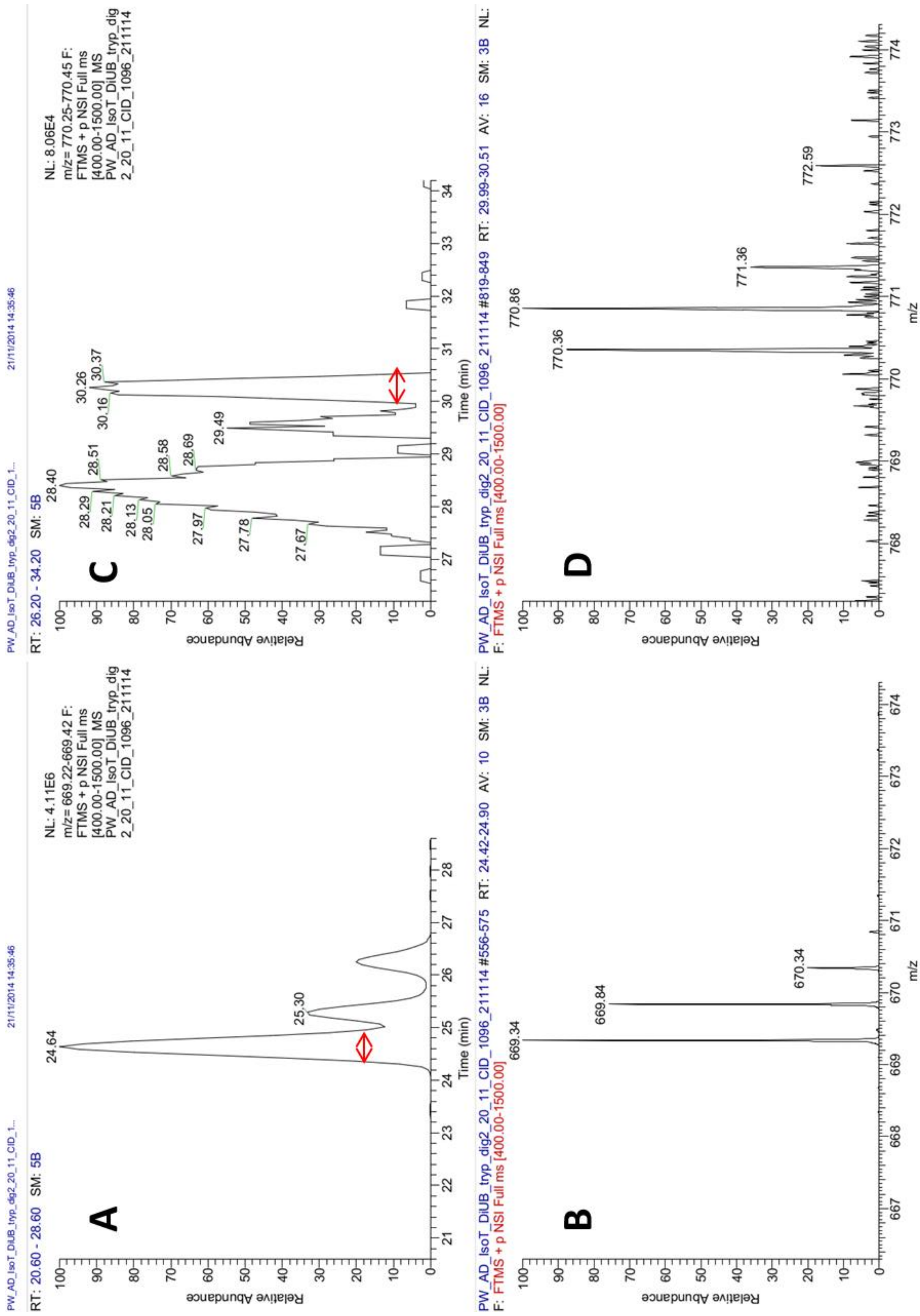

Supplementary Figure 85 – D825-R834: example of the extracted ion chromatogram (XIC) for the unlabeled and labeled precursors (A and C, respectively), and the spectra of the unlabeled and labeled precursor ions (B and D, respectively). The red arrow indicates approximately the area over which the data has been averaged.

Supplementary Tables

Sequence ID	Peptide MW	m/z	AD m/z	Unlabelled PA 1	AD PA 1	Unlabelled PA 2	AD PA 2	Unlabelled PA3	AD PA3	Fr.Mod.		
										**1**	**2**	**3**
**D18-T47**	3497.58	875.395	925.902	13005584	7278947	8285851	3106823	20150821	8764263	**0.358842**	**0.272704**	**0.303103**
**D52-N65**	1823.84	912.92	1013.935	117674747	25105261	58830625	16253246	158739562	35863598	**0.175832**	**0.216468**	**0.184291**
**D66-S86**	2274.08	759.026	826.37	152619146	23926815	30409379	9738153	196663203	43370354	**0.135527**	**0.242559**	**0.180685**
**D87-S100**	1504.69	753.345	854.36	61311122	8923503	16869812	1266599	222887726	9913157	**0.127053**	**0.069837**	**0.042582**
**I98-R112**	1674.79	838.395	939.41	163991015	29905616	242101046	41155521	273773529	37508091	**0.154235**	**0.145294**	**0.120496**
**D119-L129**	1372.7	687.35	788.365	171949322	23948399	30781927	10530437	181788743	27897945	**0.122250**	**0.254898**	**0.133046**

Supplementary Table 1 – Unbound HEWL peptide level fractional modification: tables listing all the intact peptide ions used for the data analysis with their peak areas (PA) for unlabelled and aryldiazirine (AD) modified peptides (3 repeats).

Sequence ID	Peptide MW	m/z	AD m/z	Unlabelled PA 1	AD PA 1	Unlabelled PA 2	AD PA 2	Unlabelled PA3	AD PA3	Fr.Mod.		
										**1**	**2**	**3**
**D18-T47**	3497.58	875.395	925.902	25348650	9204728	38837160	8692347	9013005	3416367	**0.2663916**	**0.182883**	**0.274862**
**D52-N65**	1823.84	912.92	1013.935	162706976	25037001	169482090	21667177	47678266	6282252	**0.1333571**	**0.113352**	**0.116423**
**D66-S86**	2274.08	759.026	826.37	214681054	28397969	206692084	30181841	100943513	12844107	**0.1168261**	**0.127417**	**0.112878**
**D87-S100**	1504.69	753.345	854.36	232281307	2242210	204350946	2490631	204532684	2478994	**0.0095607**	**0.012041**	**0.011975**
**I98-R112**	1674.79	838.395	939.41	112109304	7516966	205213413	8053905	365857357	28497766	**0.0628371**	**0.037764**	**0.072264**
**D119-L129**	1372.7	687.35	788.365	225917841	47665650	191754692	54535945	133512557	26365190	**0.1742271**	**0.221429**	**0.164908**

Supplementary Table 2 –HEWL-NAG5 peptide level fractional modification: tables listing all the intact peptide ions used for the data analysis with their peak areas (PA) for unlabelled and aryldiazirine (AD) modified peptides (3 repeats).

Sample					1				2				3			
Averaged scans					557-673				539-657				494-591			
RT					25.57-28.09				26.16-29.32				24.9-28.54			
Peptide Fr. Mod.					0.358842263				0.272703581				0.303103494			
Residue	Ion	Charge	m/z	Ad m/z	Intensity	AD Intensity	Fr.Mod	Per-residue mod.	Intensity	AD Intensity	Fr. Mod.	Per-residue mod.	Intensity	AD Intensity	Fr.Mod.	Per-residue mod.
DNYRGYSL	b8	1	969.4425	1171.47247	1410	0	0	**0**	644	0	0	**0**	597	0	0	**0**
G	b9	1	1026.464	1228.49394	459	0	0	**0**	239	0	0	**0**	300	0	0	**0**
N	b10	1	1140.507	1342.53686	1080	0	0	**0**	508	0	0	**0**	533	0	0	**0**
W	b11	2	663.7967	764.811726	844	0	0	**0**	390	0	0	**0**	508	0	0	**0**
V	b12	2	713.3309	814.345933	369	0	0	**0**	233	0	0	**0**	329	0	0	**0**
CAAKF	b17	2	1001.965	1102.98006	207	291	0.584337349	**0.209684937**	52	152	0.745098039	**0.203191**	92	168	0.646154	**0.195851**
ESN	b20	2	1167.024	1268.03883	805	1940	0.706739526	**0.043923074**	248	923	0.788215201	**0.011758**	360	936	0.722222	**0.023057**
F	b21	2	1240.558	1341.57304	449	840	0.65166796	**-0.019762006**	118	374	0.760162602	**-0.00765**	244	439	0.642753	**-0.02409**
NTQA	b25	3	965.4366	1032.77988	858	1420	0.623353819	**-0.01016031**	231	600	0.722021661	**-0.0104**	366	830	0.69398	**0.015527**
TNR	b28	3	1089.167	1156.51046	0	1320	1	**0.135156568**	0	559	1	**0.075806**	0	852	1	**0.092756**
NT							1	**0**			1	**0**			1	**0**

Supplementary Table 3 – HEWL D18-T47 unbound per-residue modification: table listing the raw data (ion intensity, and corresponding scan number and retention time (AD indicates aryldiazirine-modified peptide) and derived per residue modification

Sample					1				2				3			
Averaged scans					542-662				523-598				545-629			
RT					25.82-29.57				25.34-27.34				25.66-28.21			
Peptide Fr. Mod.					0.266391552				0.182883172				0.274862399			
Residue	Ion	Charge	m/z	Ad m/z	Intensity	AD Intensity	Fr.Mod	Per-residue mod.	Intensity	AD Intensity	Fr. Mod.	Per-residue mod.	Intensity	AD Intensity	Fr.Mod.	Per-residue mod.
DNYRGYSL	b8	1	969.4425	1171.47247	1120	0	0	0	1760	0	0	0	534	0	0	0
G	b9	1	1026.464	1228.49394	449	0	0	0	659	0	0	0	143	0	0	0
N	b10	1	1140.507	1342.53686	1020	0	0	0	1490	0	0	0	444	0	0	0
W	b11	2	663.7967	764.811726	864	55	0.059847661	0.015942911	1130	61	0.051217	0.009367	367	0	0	0
V	b12	2	713.3309	814.345933	559	21	0.036206897	-0.0062977	572	34	0.056106	0.000894	156	0	0	0
CAAKF	b17	2	1001.965	1102.98006	167	225	0.573979592	0.143258103	390	184	0.320557	0.048364	19	112	0.854962	0.234997
ESN	b20	2	1167.024	1268.03883	717	1230	0.63174114	0.015387189	1350	2320	0.632153	0.056985	346	528	0.604119	-0.06895
F	b21	2	1240.558	1341.57304	347	621	0.641528926	0.002607383	823	1200	0.593178	-0.00713	151	215	0.587432	-0.00459
NTQA	b25	3	965.4366	1032.77988	676	915	0.575109994	-0.017693442	1360	1850	0.576324	-0.00308	364	401	0.524183	-0.01738
TNR	b28	3	1089.167	1156.51046	0	1070	1	0.113187108	0	1240	1	0.077483	0	517	1	0.130784
NT							1	0			1	0			1	0

Supplementary Table 4 – HEWL D18-T47 bound per-residue modification: table listing the raw data (ion intensity, and corresponding scan number and retention time (AD indicates aryldiazirine-modified peptide) and derived per residue modification

Sequence ID	Unbound Per-Residue Mod.					Bound Per-Residue Mod.					
	1	2	3	Average	St.Dev.	1	2	3	Average	St.Dev.	P-Value
DNYRGYSL	0	0	0	**0**	**0**	0	0	0	**0**	**0**	
G	0	0	0	**0**	**0**	0	0	0	**0**	**0**	
N	0	0	0	**0**	**0**	0	0	0	**0**	**0**	
W	0	0	0	**0**	**0**	0.015943	0.009367	0	**0.008437**	**0.006542**	0.104871
V	0	0	0	**0**	**0**	-0.0063	0.000894	0	**-0.0018**	**0.0032**	0.254761
CAAKF	0.209685	0.203191	0.195851	**0.202909**	**0.005651**	0.143258	0.048364	0.234997	**0.142206**	**0.076196**	0.188971
ESN	0.043923	0.011758	0.023057	**0.026246**	**0.013324**	0.015387	0.056985	-0.06895	**0.001142**	**0.052389**	0.289401
F	-0.01976	-0.00765	-0.02409	**-0.01717**	**0.006957**	0.002607	-0.00713	-0.00459	**-0.00304**	**0.004123**	0.044986
NTQA	-0.01016	-0.0104	0.015527	**-0.00168**	**0.012166**	-0.01769	-0.00308	-0.01738	**-0.01272**	**0.006816**	0.17219
TNR	0.135157	0.075806	0.092756	**0.101239**	**0.024961**	0.113187	0.077483	0.130784	**0.107152**	**0.022175**	0.4073
NT	0	0	0	**0**	**0**	0	0	0	**0**	**0**	

Supplementary Table 5 – HEWL D18-T47 summary: per residue modification (taken from above: 3 repeats + average and standard deviation with p values).

Sample					1				2				3			
Peptide Fr. Mod.					0.358842263				0.272703581				0.303103494			
Residue	Ion	Charge	m/z	Ad m/z	Peak Area	AD Peak Area	Fr.Mod	Per-residue mod.	Peak Area	AD Peak Area	Fr. Mod.	Per-residue mod.	Peak Area	AD Peak Area	Fr.Mod.	Per-residue mod.
DNYRGYSL	b8	1	969.4425	1171.47247	268188	0	0	**0**	180051	0	0	**0**	162781	0	0	**0**
G	b9	1	1026.464	1228.49394	79892	0	0	**0**	94935	0	0	**0**	83571	0	0	**0**
N	b10	1	1140.507	1342.53686	201208	0	0	**0**	115370	0	0	**0**	139341	0	0	**0**
W	b11	2	663.7967	764.811726	148848	7718	0.049296	**0.017689**	174697	0	0	**0**	141285	0	0	**0**
V	b12	2	713.3309	814.345933	73260	4351	0.056062	**0.002428**	56464	0	0	**0**	85486	0	0	**0**
CAAKF	b17	2	1001.965	1102.98006	34251	55179	0.617008	**0.201291**	11496	35398	0.754851	**0.205851**	24536	44921	0.646745	**0.196031**
ESN	b20	2	1167.024	1268.03883	137425	324719	0.702636	**0.030727**	53216	191423	0.782471	**0.007532**	89557	236272	0.725141	**0.023762**
F	b21	2	1240.558	1341.57304	71105	146104	0.672642	**-0.01076**	24496	84312	0.774869	**-0.00207**	62701	110410	0.637799	**-0.02647**
NTQA	b25	3	965.4366	1032.77988	160017	256485	0.615807	**-0.02039**	49891	131554	0.725035	**-0.01359**	96231	211341	0.687127	**0.014952**
TNR	b28	3	1089.167	1156.51046	3601	236323	0.984991	**0.132479**	0	109849	1	**0.074984**	0	215338	1	**0.094833**
NT						0	1	**0.005386**			1	**0**			1	**0**

Supplementary Table 6 – HEWL D18-T47 unbound per-residue modification calculated using PRM: table listing the extracted ion chromatogram peak area of each fragment and derived per residue modification.

Sample					1				2				3			
Peptide Fr. Mod.					0.266391552				0.182883172				0.274862399			
Residue	Ion	Charge	m/z	Ad m/z	Peak Area	AD Peak Area	Fr.Mod	Per-residue mod.	Peak Area	AD Peak Area	Fr. Mod.	Per-residue mod.	Peak Area	AD Peak Area	Fr.Mod.	Per-residue mod.
DNYRGYSL	b8	1	969.4425	1171.47247	294251	0	0	**0**	368474	0	0	**0**	106837	0	0	**0**
G	b9	1	1026.464	1228.49394	115597	0	0	**0**	153024	0	0	**0**	28610	0	0	**0**
N	b10	1	1140.507	1342.53686	269897	0	0	**0**	322333	0	0	**0**	90781	0	0	**0**
W	b11	2	663.7967	764.811726	224451	11837	0.050096	**0.015943**	287001	13432	0.044709	**0.008176**	74564	0	0	**0**
V	b12	2	713.3309	814.345933	141416	3169	0.021918	**-0.0063**	169160	23917	0.123873	**0.014478**	31473	0	0	**0**
CAAKF	b17	2	1001.965	1102.98006	39917	56888	0.587656	**0.143258**	66453	67002	0.502057	**0.069163**	3518	23710	0.870795	**0.234997**
ESN	b20	2	1167.024	1268.03883	165687	299098	0.643519	**0.015387**	192003	376815	0.662453	**0.029334**	57312	97546	0.629906	**-0.06895**
F	b21	2	1240.558	1341.57304	81584	151029	0.649272	**0.002607**	116425	198516	0.630328	**-0.00588**	22455	42178	0.652577	**-0.00459**
NTQA	b25	3	965.4366	1032.77988	174880	235606	0.573968	**-0.01769**	211746	332701	0.611081	**-0.00352**	58233	73549	0.558111	**-0.01738**
TNR	b28	3	1089.167	1156.51046	1690	266328	0.993694	**0.113187**	0	346968	1	**0.071127**	0	91058	1	**0.130784**
NT							1	**0**			1	**0**			1	0

Supplementary Table 7 – HEWL D18-T47 bound per-residue modification calculated using PRM: table listing the extracted ion chromatogram peak area of each fragment and derived per residue modification.

Sequence ID	Unbound Per-Residue Mod.					Bound Per-Residue Mod.					
	1	2	3	Average	St.Dev.	1	2	3	Average	St.Dev.	P-Value
DNYRGYSL	0	0	0	**0**	**0**	0	0	0	**0**	**0**	
G	0	0	0	**0**	**0**	0	0	0	**0**	**0**	
N	0	0	0	**0**	**0**	0	0	0	**0**	**0**	
W	0.017689	0	0	**0.005896**	**0.008339**	0.015943	0.008176	0	**0.00804**	**0.006509**	0.394348
V	0.002428	0	0	**0.000809**	**0.001145**	-0.0063	0.014478	0	**0.002727**	**0.008698**	0.393242
CAAKF	0.201291	0.205851	0.196031	**0.201058**	**0.004012**	0.143258	0.069163	0.234997	**0.149139**	**0.067829**	0.196429
ESN	0.030727	0.007532	0.023762	**0.020674**	**0.009718**	0.015387	0.029334	-0.06895	**-0.00808**	**0.043418**	0.228639
F	-0.01076	-0.00207	-0.02647	**-0.0131**	**0.010098**	0.002607	-0.00588	-0.00459	**-0.00262**	**0.003732**	0.131089
NTQA	-0.02039	-0.01359	0.014952	**-0.00634**	**0.015313**	-0.01769	-0.00352	-0.01738	**-0.01287**	**0.00661**	0.309405
TNR	0.132479	0.074984	0.094833	**0.100765**	**0.023844**	0.113187	0.071127	0.130784	**0.105033**	**0.025028**	0.434942
NT	0.005386	0	0	**0.001795**	**0.002539**	0	0	0	**0**	**0**	0.211325

Supplementary Table 8 – HEWL D18-T47 per-residue modification summary: per residue modification calculated using PRM analysis (taken from above: 3 repeats + average and standard deviation with p values).

Sample					1				2				3			
Averaged scans					759-895				661-749				670-806			
RT					28.26-31.61				28.2-31.1				27.31-30.91			
Peptide Fr. Mod.					0.175831766				0.216467875				0.184290933			
Residue	Ion	Charge	m/z	Ad m/z	Intensity	AD Intensity	Fr.Mod	Per-residue mod.	Intensity	AD Intensity	Fr. Mod.	Per-residue mod.	Intensity	AD Intensity	Fr.Mod.	Per-residue mod.
DYG	b3	1	336.119	538.149	199	0	0	**0**	181	0	0	**0**	344	0	0	**0**
I	b4	1	449.2031	651.2331	448	11.5	0.025027	**0.004400577**	396	0	0	**0**	852	47	0.05228	**0.009634787**
L	b5	1	562.2871	764.3171	737	66.5	0.082763	**0.010151772**	620	13.2	0.020846	**0.004512596**	1220	164	0.118497	**0.012203156**
QIN	b8	1	917.4727	1119.503	433	150	0.25729	**0.030687385**	404	17	0.04038	**0.004228387**	748	263	0.260138	**0.02610322**
SR	b10	1	1160.606	1362.636	2680	4070	0.602963	**0.060780309**	2600	2110	0.447983	**0.088232948**	3900	5940	0.603659	**0.063307632**
W	b11	1	1346.685	1548.715	496	4600	0.902669	**0.0526978**	354	3050	0.896005	**0.096982302**	643	6820	0.913842	**0.05716393**
W	b12	1	1532.764	1734.794	227	6020	0.963663	**0.010724647**	139	4160	0.967667	**0.015512564**	218	8920	0.976144	**0.011481686**
C	b13	2	846.9012	947.9162	28	4580	0.993924	**0.005320854**	22	3800	0.994244	**0.005753056**	52	9110	0.994324	**0.003350558**
N							1	**0.001068422**			1	**0.001246021**			1	**0.001045965**

Supplementary Table 9 – HEWL D52-N65 unbound per-residue modification: table listing the raw data (ion intensity, and corresponding scan number and retention time (AD indicates aryldiazirine-modified peptide) and derived per residue modification

Sample					1				2				3			
Averaged scans					677-765				701-798				707-783			
RT					27.47-29.85				28-30.52				27.99-30.58			
Peptide Fr. Mod.					0.133357146				0.113352132				0.116423123			
Residue	Ion	Charge	m/z	Ad m/z	Intensity	AD Intensity	Fr.Mod	Per-residue mod.	Intensity	AD Intensity	Fr. Mod.	Per-residue mod.	Intensity	AD Intensity	Fr.Mod.	Per-residue mod.
DYG	b3	1	336.119	538.149	291	0	0	**0**	307	0	0	**0**	37.5	0	0	**0**
I	b4	1	449.2031	651.2331	623	0	0	**0**	744	0	0	**0**	105	0	0	**0**
L	b5	1	562.2871	764.3171	1020	0	0	**0**	1190	0	0	**0**	200	0	0	**0**
QIN	b8	1	917.4727	1119.503	711	0	0	**0**	781	0	0	**0**	126	0	0	**0**
SR	b10	1	1160.606	1362.636	830	6410	0.885359	**0.118068965**	677	7770	0.919853	**0.104267322**	136	1590	0.921205	**0.107249574**
W	b11	1	1346.685	1548.715	198	6260	0.96934	**0.011199498**	208	7210	0.97196	**0.005906428**	25	1450	0.983051	**0.007200275**
W	b12	1	1532.764	1734.794	131	7730	0.983335	**0.001866347**	161	8790	0.982013	**0.001139539**	21.6	1770	0.987944	**0.000569646**
C	b13	2	846.9012	947.9162	20	7430	0.997315	**0.001864331**	29.4	8620	0.996601	**0.001653551**	0	1270	1	**0.001403628**
N							1	**0.000358006**			1	**0.000385293**			1	**0**

Supplementary Table 10 – HEWL D52-N65 bound per-residue modification: table listing the raw data (ion intensity, and corresponding scan number and retention time (AD indicates aryldiazirine-modified peptide) and derived per residue modification

Sequence ID	Unbound Per-Residue Mod.					Bound Per-Residue Mod.					
	1	2	3	Average	St.Dev.	1	2	3	Average	St.Dev.	P-Value
DYG	0	0	0	**0**	**0**	0	0	0	**0**	**0**	
I	0.004401	0	0.009635	**0.004678**	**0.003938**	0	0	0	**0**	**0**	0.117486
**L**	0.010152	0.004513	0.012203	**0.008956**	**0.003252**	0	0	0	**0**	**0**	**0.030017**
**QIN**	0.030687	0.004228	0.026103	**0.02034**	**0.011545**	0	0	0	**0**	**0**	**0.032949**
**SR**	0.06078	0.088233	0.063308	**0.070774**	**0.012389**	0.118069	0.104267	0.10725	**0.109862**	**0.00593**	**0.006161**
**W**	0.052698	0.096982	0.057164	**0.068948**	**0.019907**	0.011199	0.005906	0.0072	**0.008102**	**0.002253**	**0.025081**
**W**	0.010725	0.015577	0.011482	**0.012594**	**0.002132**	0.001866	0.00114	0.00057	**0.001192**	**0.000531**	**0.009027**
C	0.005321	0.005689	0.003351	**0.004787**	**0.001027**	0.001864	0.001654	0.001404	**0.001641**	**0.000188**	0.025429
N	0.001068	0.001246	0.001046	**0.00112**	**8.95E-05**	0.000358	0.000385	0	**0.000248**	**0.000176**	0.004111

Supplementary Table 11 – HEWL D52-N65 summary: per residue modification (taken from above: 3 repeats + average and standard deviation with p values). Residues showing significant differences between bound and unbound are highlighted in red.

Sample					1				2				3			
Peptide Fr. Mod.					0.175831766				0.216467875				0.184290933			
Residue	Ion	Charge	m/z	Ad m/z	Peak Area	AD Peak Area	Fr.Mod	Per-residue mod.	Peak Area	AD Peak Area	Fr. Mod.	Per-residue mod.	Peak Area	AD Peak Area	Fr.Mod.	Per-residue mod.
DYG	b3	1	336.119	538.149	42608	3819	0.082258	**0.014464**	36090	2290	0.059666	**0.012916**	75235	5368	0.066598	**0.012273**
I	b4	1	449.2031	651.2331	95762	9115	0.086911	**0.000818**	73781	1603	0.021264	**-0.00831**	182482	14848	0.075245	**0.001593**
L	b5	1	562.2871	764.3171	167103	18039	0.097433	**0.00185**	119453	3891	0.031546	**0.002226**	264092	41697	0.136359	**0.011263**
QIN	b8	1	917.4727	1119.503	101138	45637	0.310932	**0.03754**	84008	11052	0.116263	**0.018339**	174739	71318	0.289843	**0.028286**
SR	b10	1	1160.606	1362.636	574706	899595	0.610184	**0.052618**	516068	457972	0.470178	**0.076611**	919224	1457486	0.613237	**0.059598**
W	b11	1	1346.685	1548.715	107529	994433	0.90242	**0.051384**	81939	647920	0.887733	**0.090387**	169918	1674001	0.90785	**0.054294**
W	b12	1	1532.764	1734.794	56706	1287266	0.957807	**0.009739**	35841	848065	0.959452	**0.015525**	65364	2157809	0.970599	**0.011564**
C	b13	2	846.9012	947.9162	6691	1069713	0.993784	**0.006326**	9631	847830	0.988768	**0.006346**	20475	2347133	0.991352	**0.003825**
N							1	**0.001093**			1	**0.002431**			1	**0.001594**

Supplementary Table 12 – HEWL D52-N65 unbound per-residue modification calculated using PRM: table listing the extracted ion chromatogram peak area of each fragment and derived per residue modification.

Sample					**1**				**2**				**3**			
Peptide Fr. Mod.					0.133357146				0.113352132				0.116423123			
Residue	Ion	Charge	m/z	Ad m/z	Peak Area	AD Peak Area	Fr.Mod	Per-residue mod.	Peak Area	AD Peak Area	Fr. Mod.	Per-residue mod.	Peak Area	AD Peak Area	Fr.Mod.	Per-residue mod.
DYG	b3	1	336.119	538.149	46258	4572	0.089947	**0.011995**	51388	4770	0.084939	**0.009628**	6446	0	0	**0**
I	b4	1	449.2031	651.2331	99886	5863	0.055443	**-0.0046**	121427	4917	0.038918	**-0.00522**	16275	0	0	**0**
L	b5	1	562.2871	764.3171	162149	7724	0.045469	**-0.00133**	197447	8436	0.040975	**0.000233**	33921	0	0	**0**
QIN	b8	1	917.4727	1119.503	110194	10967	0.090516	**0.006007**	143156	19929	0.1222	**0.009207**	21172	0	0	**0**
SR	b10	1	1160.606	1362.636	208437	1003098	0.827956	**0.098343**	217996	1328915	0.859077	**0.083527**	35117	249850	0.876768	**0.102076**
W	b11	1	1346.685	1548.715	60350	947115	0.940097	**0.014955**	80357	1280641	0.940957	**0.009281**	5526	231567	0.976693	**0.011634**
W	b12	1	1532.764	1734.794	32244	1171660	0.973217	**0.004417**	56815	1507734	0.963686	**0.002576**	5719	275565	0.979668	**0.000346**
C	b13	2	846.9012	947.9162	6853	1167938	0.994167	**0.002794**	14261	1542407	0.990839	**0.003078**	2453	219811	0.988964	**0.001082**
N							1	**0.000778**			1	**0.001038**			1	**0.001285**

Supplementary Table 13 – HEWL D52-N65 bound per-residue modification calculated using PRM: table listing the extracted ion chromatogram peak area of each fragment and derived per residue modification.

Sequence ID	Unbound Per-Residue Mod.					Bound Per-Residue Mod.					
	1	2	3	Average	St.Dev.	1	2	3	Average	St.Dev.	P-Value
DYG	0.014464	0.012916	0.012273	0.013218	0.000919	0.011995	0.009628	0	0.007208	0.005187	0.124007
I	0.000818	-0.00831	0.001593	-0.00197	0.004498	-0.0046	-0.00522	0	-0.00327	0.002328	0.369813
L	0.00185	0.002226	0.011263	0.005113	0.004351	-0.00133	0.000233	0	-0.00037	0.000689	0.110357
QIN	0.03754	0.018339	0.028286	0.028055	0.007841	0.006007	0.009207	0	0.005071	0.003817	0.016818
SR	0.052618	0.076611	0.059598	0.062943	0.010076	0.098343	0.083527	0.102076	0.094649	0.008011	0.012639
W	0.051384	0.090387	0.054294	0.065355	0.01774	0.014955	0.009281	0.011634	0.011957	0.002327	0.025905
W	0.009739	0.015525	0.011564	0.012276	0.002415	0.004417	0.002576	0.000346	0.002447	0.001664	0.004521
C	0.006326	0.006346	0.003825	0.005499	0.001184	0.002794	0.003078	0.001082	0.002318	0.000881	0.019055
N	0.001093	0.002431	0.001594	0.001706	0.000552	0.000778	0.001038	0.001285	0.001034	0.000207	0.102639

Supplementary Table 14 – HEWL D52-N65 per-residue modification summary: per residue modification calculated using PRM analysis (taken from above: 3 repeats + average and standard deviation with p values). Residues showing significant differences between bound and unbound are highlighted in red

Sample					1				2				3			
Averaged scans					505-685				477-589				468-628			
RT					23.58-27.38				24.48-28.58				23.03-27.24			
Peptide Fr. Mod.					0.1355274				0.242559194				0.186418633			
Residue	Ion	Charge	m/z	Ad m/z	Intensity	AD Intensity	Fr.Mod	Per-residue mod.	Intensity	AD Intensity	Fr. Mod.	Per-residue mod.	Intensity	AD Intensity	Fr.Mod.	Per-residue mod.
DGRTPGSRNL	b10	2	527.7731	628.7881	172	671	0.795967	**0.107875309**	18.5	124	0.870175	**0.211069**	130	523	0.800919	**0.149306194**
CN	b12	2	664.8098	765.8248	289	5530	0.950335	**0.020921138**	30.1	1130	0.974054	**0.025197**	199	3580	0.947341	**0.027295739**
IPC	b15	2	849.8936	950.9086	230	2090	0.900862	**-0.006704952**	14.4	432	0.967742	**-0.00153**	123	1410	0.919765	**-0.005140568**
SA	b17	2	928.9282	1029.943	792	15600	0.951684	**0.00688773**	96.6	3160	0.970337	**0.000629**	517	9830	0.950034	**0.005642642**
L	b18	2	985.4702	1086.485	1740	32700	0.949477	**-0.000299027**	289	6740	0.958885	**-0.00278**	936	21300	0.957906	**0.001467538**
L	b19	2	1042.012	1143.027	850	55800	0.984996	**0.004813694**	168	10500	0.984252	**0.006153**	391	38800	0.990023	**0.00598723**
SS							1	**0.002033509**			1	**0.00382**			1	**0.001859858**

Supplementary Table 15 – HEWL D66-N86 unbound per-residue modification: table listing the raw data (ion intensity, and corresponding scan number and retention time (AD indicates aryldiazirine-modified peptide) and derived per residue modification

Sample					1				2				3			
Averaged scans					459-593				443-648				456-593			
RT					22.68-26.19				21.97-27.04				22.98-26.92			
Peptide Fr. Mod.					0.116826078				0.127417321				0.112877895			
Residue	Ion	Charge	m/z	Ad m/z	Intensity	AD Intensity	Fr.Mod	Per-residue mod.	Intensity	AD Intensity	Fr. Mod.	Per-residue mod.	Intensity	AD Intensity	Fr.Mod.	Per-residue mod.
DGRTPGSRNL	b10	2	527.7731	628.7881	82.8	454	0.845753	0.098806	71.7	627	0.897381	**0.114341864**	43.1	230	0.842182	**0.095064**
CN	b12	2	664.8098	765.8248	116	2810	0.995889	0.013389	115	3760	0.970323	**0.00929404**	49.7	1830	0.97356	**0.01483**
IPC	b15	2	849.8936	950.9086	101	1090	0.915197	-0.00528	117	1560	0.930233	**-0.005108163**	77.4	629	0.89043	**-0.00938**
SA	b17	2	928.9282	1029.943	270	7500	0.965251	0.005848	205	9750	0.979407	**0.006265718**	132	4610	0.972164	**0.009226**
L	b18	2	985.4702	1086.485	703	16100	0.958162	-0.00083	552	20200	0.9734	**-0.000765419**	420	10700	0.96223	**-0.00112**
L	b19	2	1042.012	1143.027	206	28200	0.992748	0.004041	251	35400	0.99296	**0.002492203**	169	16100	0.989612	**0.003091**
SS							1	0.000847			1	**0.000897079**			1	**0.001173**

Supplementary Table 16 – HEWL D66-N86 bound per-residue modification: table listing the raw data (ion intensity, and corresponding scan number and retention time (AD indicates aryldiazirine-modified peptide) and derived per residue modification

Sequence ID	Unbound Per-Residue Mod.					Bound Per-Residue Mod.					
	1	2	3	Average	St.Dev.	1	2	3	Average	St.Dev.	P-Value
DGRTPGSRNL	0.107875	0.211069	0.149306	0.156084	0.0424	0.098806	0.114342	0.095064	0.102737	0.008347	0.111481
CN	0.020921	0.025197	0.027296	0.024471	0.002653	0.013389	0.009294	0.01483	0.012504	0.002345	0.004387
IPC	-0.0067	-0.00153	-0.00514	-0.00446	0.002167	-0.00528	-0.00511	-0.00938	-0.00659	0.001977	0.181207
SA	0.006888	0.000629	0.005643	0.004387	0.002705	0.005848	0.006266	0.009226	0.007113	0.001504	0.150618
L	-0.0003	-0.00278	0.001468	-0.00054	0.001741	-0.00083	-0.00077	-0.00112	-0.0009	0.000155	0.396879
L	0.004814	0.006153	0.005987	0.005651	0.000596	0.004041	0.002492	0.003091	0.003208	0.000637	0.008345
SS	0.002034	0.00382	0.00186	0.002571	0.000886	0.000847	0.000897	0.001173	0.000972	0.000143	0.063981

Supplementary Table 17 – HEWL D66-N86 summary: per residue modification (taken from above: 3 repeats + average and standard deviation with p values). Residues showing significant differences between bound and unbound are highlighted in red.

Sample					1				2				3			
Peptide Fr. Mod.					0.1355274				0.242559194				0.186418633			
Residue	Ion	Charge	m/z	Ad m/z	Peak Area	AD Peak Area	Fr.Mod	Per-residue mod.	Peak Area	AD Peak Area	Fr. Mod.	Per-residue mod.	Peak Area	AD Peak Area	Fr.Mod.	Per-residue mod.
DGRTPGSRNL	b10	2	527.7731	628.7881	46220	196161	0.809308	**0.109683**	4125	37342	0.900523	**0.21843**	35579	154121	0.812446	**0.151455**
CN	b12	2	664.8098	765.8248	73588	1807088	0.960872	**0.020541**	7781	374479	0.979645	**0.019192**	52186	1224557	0.959126	**0.027344**
IPC	b15	2	849.8936	950.9086	64508	628403	0.906903	**-0.00731**	7567	137861	0.947967	**-0.00768**	37783	442616	0.921351	**-0.00704**
SA	b17	2	928.9282	1029.943	222146	4782944	0.955616	**0.006602**	27534	1010137	0.973466	**0.006185**	151065	3171361	0.954532	**0.006186**
L	b18	2	985.4702	1086.485	481934	9043203	0.949404	**-0.00084**	95081	1960062	0.953735	**-0.00479**	338015	6248022	0.948677	**-0.00109**
L	b19	2	1042.012	1143.027	229892	15327958	0.985223	**0.004855**	49138	2975716	0.983755	**0.007282**	132163	11342314	0.988482	**0.00742**
SS							1	**0.002003**			1	**0.00394**			1	**0.002147**

Supplementary Table 18 – HEWL D66-N86 unbound per-residue modification calculated using PRM: table listing the extracted ion chromatogram peak area of each fragment and derived per residue modification.

Sample					1				2				3			
Peptide Fr. Mod.					0.116826078				0.127417321				0.112877895			
Residue	Ion	Charge	m/z	Ad m/z	Peak Area	AD Peak Area	Fr.Mod	Per-residue mod.	Peak Area	AD Peak Area	Fr. Mod.	Per-residue mod.	Peak Area	AD Peak Area	Fr.Mod.	Per-residue mod.
DGRTPGSRNL	b10	2	527.7731	628.7881	17073	124864	0.879714	**0.102774**	24210	209082	0.896224	**0.114195**	10508	80195	0.884149	**0.099801**
CN	b12	2	664.8098	765.8248	29536	835662	0.965862	**0.010064**	36612	1375129	0.974066	**0.009918**	15191	596002	0.975145	**0.010271**
IPC	b15	2	849.8936	950.9086	38515	303251	0.887306	**-0.00918**	50606	517126	0.910863	**-0.00805**	28055	205462	0.879859	**-0.01076**
SA	b17	2	928.9282	1029.943	70136	2166405	0.968641	**0.009502**	74487	3480669	0.979048	**0.008688**	42202	1449800	0.971715	**0.010368**
L	b18	2	985.4702	1086.485	241577	4206284	0.945687	**-0.00268**	303792	6640074	0.95625	**-0.0029**	136901	2990763	0.956229	**-0.00175**
L	b19	2	1042.012	1143.027	75870	7216431	0.989596	**0.00513**	90999	11191950	0.991935	**0.004547**	45813	4402007	0.9897	**0.003778**
SS							1	**0.001215**			1	**0.001028**			1	**0.001163**

Supplementary Table 19 – HEWL D66-N86 bound per-residue modification calculated using PRM: table listing the extracted ion chromatogram peak area of each fragment and derived per residue modification.

Sequence ID	Unbound Per-Residue Mod.					Bound Per-Residue Mod.					
	1	2	3	Average	St.Dev.	1	2	3	Average	St.Dev.	P-Value
DGRTPGSRNL	0.109683	0.21843	0.151455	0.159856	0.044791	0.102774	0.114195	0.099801	0.10559	0.006204	0.115878
CN	0.020541	0.019192	0.027344	0.022359	0.003568	0.010064	0.009918	0.010271	0.010085	0.000145	0.019903
IPC	-0.00731	-0.00768	-0.00704	-0.00735	0.000263	-0.00918	-0.00805	-0.01076	-0.00933	0.001108	0.0665
SA	0.006602	0.006185	0.006186	0.006324	0.000196	0.009502	0.008688	0.010368	0.00952	0.000686	0.012025
L	-0.00084	-0.00479	-0.00109	-0.00224	0.001803	-0.00268	-0.0029	-0.00175	-0.00244	0.000501	0.445532
L	0.004855	0.007282	0.00742	0.006519	0.001178	0.00513	0.004547	0.003778	0.004485	0.000554	0.057069
SS	0.002003	0.00394	0.002147	0.002697	0.000881	0.001215	0.001028	0.001163	0.001135	7.91E-05	0.064996

Supplementary Table 20 – HEWL D66-N86 per-residue modification summary: per residue modification calculated using PRM analysis (taken from above: 3 repeats + average and standard deviation with p values). Residues showing significant differences between bound and unbound are highlighted in red

Sample					1				2				3			
Averaged scans					428-609				427-522				416-517			
RT					21.95-26.71				22.1-25.53				21.47-25.62			
Peptide Fr. Mod.					0.127052761				0.069837356				0.042582128			
Residue	Ion	Charge	m/z	Ad m/z	Intensity	AD Intensity	Fr.Mod	Per-residue mod.	Intensity	AD Intensity	Fr. Mod.	Per-residue mod.	Intensity	AD Intensity	Fr.Mod.	Per-residue mod.
DITA	b4	1	401.2031	603.2331	14	0	0	**0**	11.4	0	0	**0**	0	0	0	**0**
S	b5	1	488.2351	690.2651	30.1	0	0	**0**	11	0	0	**0**	16.5	0	0	**0**
V	b6	1	587.3035	789.3335	117	0	0	**0**	84.7	0	0	**0**	71.7	0	0	**0**
NCAK	b10	1	1060.509	1262.539	553	308	0.357724	**0.045449768**	387	59.1	0.132482	**0.009252158**	340	130	0.276596	**0.011778035**
K	b11	1	1188.604	1390.634	421	793	0.653213	**0.037542686**	110	394	0.781746	**0.045342918**	187	424	0.693944	**0.017771592**
I	b12	2	651.3477	752.3627	113	2690	0.959686	**0.038938308**	20.8	1540	0.986674	**0.014311593**	52.2	1470	0.965708	**0.011572254**
V	b13	2	700.8819	801.8969	129	4450	0.971828	**0.001542657**	60.9	2560	0.976764	**-0.000692074**	59.8	2400	0.975689	**0.000425036**
S							1	**0.003579342**			1	**0.001622761**			1	**0.001035211**

Supplementary Table 21 – HEWL D87-S100 unbound per-residue modification: table listing the raw data (ion intensity, and corresponding scan number and retention time (AD indicates aryldiazirine-modified peptide) and derived per residue modification

Sample					1				2				3			
Averaged scans					433-546				410-515				434-518			
RT					22.18-25.85				21.36-25.12				22.42-26.31			
Peptide Fr. Mod.					0.009560704				0.012041249				0.011975141			
Residue	Ion	Charge	m/z	Ad m/z	Intensity	AD Intensity	Fr.Mod	Per-residue mod.	Intensity	AD Intensity	Fr. Mod.	Per-residue mod.	Intensity	AD Intensity	Fr.Mod.	Per-residue mod.
DITA	b4	1	401.2031	603.2331	0	0	0	**0**	0	0	0	**0**	0	0	0	**0**
S	b5	1	488.2351	690.2651	5	0	0	**0**	0	0	0	**0**	5	0	0	**0**
V	b6	1	587.3035	789.3335	30.4	0	0	**0**	25.7	0	0	**0**	17.7	0	0	**0**
NCAK	b10	1	1060.509	1262.539	148	10.8	0.06801	**0.000650224**	115	54	0.319527	**0.0038475**	91.8	48.2	0.344286	**0.00412287**
K	b11	1	1188.604	1390.634	43.2	200	0.822368	**0.007212197**	89.1	178	0.666417	**0.004176994**	36.4	157	0.811789	**0.005598418**
I	b12	2	651.3477	752.3627	14.5	636	0.977709	**0.00148517**	15.6	647	0.976456	**0.003733261**	13	428	0.970522	**0.001900844**
V	b13	2	700.8819	801.8969	28.7	1020	0.972633	**-4.85365E-05**	46.1	1030	0.95716	**-0.000232351**	25.5	748	0.967033	**-4.17762E-05**
S							1	**0.00026165**			1	**0.000515846**			1	**0.000394785**

Supplementary Table 22 – HEWL D87-S100 bound per-residue modification: table listing the raw data (ion intensity, and corresponding scan number and retention time (AD indicates aryldiazirine-modified peptide) and derived per residue modification

Sequence ID	Unbound Per-Residue Mod.					Bound Per-Residue Mod.					
	1	2	3	Average	St.Dev.	1	2	3	Average	St.Dev.	P-Value
DITA	0	0	0	0	0	0	0	0	0	0	
S	0	0	0	0	0	0	0	0	0	0	
V	0	0	0	0	0	0	0	0	0	0	
NCAK	0.04545	0.009252	0.011778	0.02216	0.016501	0.00065	0.003847	0.004123	0.002874	0.001576	0.120804
K	0.037543	0.045343	0.017772	0.033552	0.011604	0.007212	0.004177	0.005598	0.005663	0.00124	0.038753
I	0.038938	0.014312	0.011572	0.021607	0.012306	0.001485	0.003733	0.001901	0.002373	0.000977	0.079207
V	0.001543	-0.00069	0.000425	0.000425	0.000912	-4.9E-05	-0.00023	-4.2E-05	-0.00011	8.83E-05	0.248739
S	0.003579	0.001623	0.001035	0.002079	0.001088	0.000262	0.000516	0.000395	0.000391	0.000104	0.080226

Supplementary Table 23 – HEWL D87-S100 summary: per residue modification (taken from above: 3 repeats + average and standard deviation with p values). Residues showing significant differences between bound and unbound are highlighted in red.

Sample					1				2				3			
Peptide Fr. Mod.					0.127052761				0.069837356				0.042582128			
Residue	Ion	Charge	m/z	Ad m/z	Peak Area	AD Peak Area	Fr.Mod	Per-residue mod.	Peak Area	AD Peak Area	Fr. Mod.	Per-residue mod.	Peak Area	AD Peak Area	Fr.Mod.	Per-residue mod.
DITA	b4	1	401.2031	603.2331	5024	0	0	**0**	4114	0	0	**0**	1855	0	0	**0**
S	b5	1	488.2351	690.2651	15357	0	0	**0**	3678	0	0	**0**	6247	0	0	**0**
V	b6	1	587.3035	789.3335	55207	0	0	**0**	23094	0	0	**0**	27704	0	0	**0**
NCAK	b10	1	1060.509	1262.539	202943	102040	0.334576	**0.042508808**	102206	15364	0.13068	**0.009126317**	95499	41812	0.304506	**0.012966506**
K	b11	1	1188.604	1390.634	131200	281207	0.681868	**0.044124362**	24749	100424	0.802282	**0.046902912**	57099	135939	0.704208	**0.01702019**
I	b12	2	651.3477	752.3627	45984	986677	0.95547	**0.03476198**	6822	378923	0.982315	**0.012573036**	16438	451418	0.964865	**0.01109932**
V	b13	2	700.8819	801.8969	47125	1601020	0.971407	**0.002024823**	16671	631855	0.974294	**-0.000560146**	26649	707628	0.963707	**-4.93144E-05**
S							1	**0.003632788**			1	**0.001795238**			1	**0.001545426**

Supplementary Table 24 – HEWL D87-S100 unbound per-residue modification calculated using PRM: table listing the extracted ion chromatogram peak area of each fragment and derived per residue modification.

Sample					1				2				3			
Peptide Fr. Mod.					0.009560704				0.012041249				0.011975141			
Residue	Ion	Charge	m/z	Ad m/z	Peak Area	AD Peak Area	Fr.Mod	Per-residue mod.	Peak Area	AD Peak Area	Fr. Mod.	Per-residue mod.	Peak Area	AD Peak Area	Fr.Mod.	Per-residue mod.
DITA	b4	1	401.2031	603.2331	0	0	0	**0**	0	0	0	**0**	0	0	0	**0**
S	b5	1	488.2351	690.2651	1663	0	0	**0**	0	0	0	**0**	2467	0	0	**0**
V	b6	1	587.3035	789.3335	8886	0	0	**0**	14376	0	0	**0**	5548	0	0	**0**
NCAK	b10	1	1060.509	1262.539	37240	4727	0.112636	**0.001076881**	34898	13299	0.27593	**0.003322542**	28983	13766	0.322019	**0.003856226**
K	b11	1	1188.604	1390.634	10304	54495	0.840985	**0.00696353**	21326	52398	0.710732	**0.005235558**	9577	45799	0.827055	**0.006047875**
I	b12	2	651.3477	752.3627	5050	185324	0.973473	**0.001266679**	4460	187504	0.976766	**0.003203388**	4149	126569	0.96826	**0.001690948**
V	b13	2	700.8819	801.8969	16513	276039	0.943555	**-0.000286037**	12794	296665	0.958657	**-0.000218062**	7268	230161	0.969389	**1.35178E-05**
S							1	**0.000539651**			1	**0.000497823**			1	**0.000366574**

Supplementary Table 25 – HEWL D87-S100 bound per-residue modification calculated using PRM: table listing the extracted ion chromatogram peak area of each fragment and derived per residue modification.

Sequence ID	Unbound Per-Residue Mod.					Bound Per-Residue Mod.					
	1	2	3	Average	St.Dev.	1	2	3	Average	St.Dev.	P-Value
DITA	0	0	0	0	0	0	0	0	0	0	
S	0	0	0	0	0	0	0	0	0	0	
V	0	0	0	0	0	0	0	0	0	0	
NCAK	0.042509	0.009126	0.012967	0.021534	0.014914	0.001077	0.003323	0.003856	0.002752	0.001204	0.108927
**K**	0.044124	0.046903	0.01702	0.036016	0.01348	0.006964	0.005236	0.006048	0.006082	0.000706	0.0442
I	0.034762	0.012573	0.011099	0.019478	0.010824	0.001267	0.003203	0.001691	0.002054	0.000831	0.075625
V	0.002025	-0.00056	-4.9E-05	0.000472	0.001118	-0.00029	-0.00022	1.35E-05	-0.00016	0.000128	0.254154
S	0.003633	0.001795	0.001545	0.002324	0.000931	0.00054	0.000498	0.000367	0.000468	7.37E-05	0.053307

Supplementary Table 26 – HEWL D87-S100 per-residue modification summary: per residue modification calculated using PRM analysis (taken from above: 3 repeats + average and standard deviation with p values). Residues showing significant differences between bound and unbound are highlighted in red

Sample					1				2				3			
Averaged scans					788-920				768-899				762-898			
RT					30.14-32.9				28.29-30.82				28-30.63			
Peptide Fr. Mod.					0.154234841				0.145294146				0.120495682			
Residue	Ion	Charge	m/z	Ad m/z	Intensity	AD Intensity	Fr.Mod	Per-residue mod.	Intensity	AD Intensity	Fr. Mod.	Per-residue mod.	Intensity	AD Intensity	Fr.Mod.	Per-residue mod.
I							1	**0**			1	**0**			1	**0**
VS	y14	2	781.8621	882.8771	0	235	1	**0**	0	470	1	**0**	0	213	1	**0.003272986**
DGNG	y12	1	1376.616	1578.646	0	536	1	**0.037637**	0	933	1	**0.030785**	18.4	659	0.972837	**0.025447024**
M	y8	1	1033.504	1235.534	878	2720	0.755976	**0.050515**	1070	3980	0.788119	**0.048113**	895	2860	0.761651	**0.038111054**
N	y7	1	902.4631	1104.493	6990	5240	0.428455	**0.024238**	9340	7860	0.456977	**0.030026**	7360	5910	0.445365	**0.026567503**
AW	y6	1	788.4202	990.4502	4190	1560	0.271304	**0.035643**	5900	1970	0.250318	**0.030635**	4860	1410	0.22488	**0.024409109**
V	y4	1	531.3038	733.3338	12700	532	0.040206	**0.001201**	17400	715	0.03947	**0.001723**	14200	324	0.022308	**0.000414503**
A	y3	1	432.2354	634.2654	15700	526	0.032417	**0.005**	21200	602	0.027612	**0.004012**	16900	325	0.018868	**0.002273503**
WR	y2	1	361.1983	563.2283	1800	0	0	**0**	2550	0	0	**0**	1890	0	0	**0**

Supplementary Table 27 – HEWL I98-R112 unbound per-residue modification: table listing the raw data (ion intensity, and corresponding scan number and retention time (AD indicates aryldiazirine-modified peptide) and derived per residue modification

Sample					1				2				3			
Averaged scans					1037-1241				1036-1243				1091-1360			
RT					39.21-43.74				37.42-41.65				36.71-41.63			
Peptide Fr. Mod.					0.062837084				0.037764366				0.072264222			
Residue	Ion	Charge	m/z	Ad m/z	Intensity	AD Intensity	Fr.Mod	Per-residue mod.	Intensity	AD Intensity	Fr. Mod.	Per-residue mod.	Intensity	AD Intensity	Fr.Mod.	Per-residue mod.
I							1	**0**			1	**0**			1	**0**
VS	y14	2	781.8621	882.8771	0	0	1	**0**	0	0	1	**0**	0	98.7	1	**0**
DGNG	y12	1	1376.616	1578.646	0	0	1	**0.015246**	0	92.6	1	**0.005105**	0	320	1	**0.01791409**
M	y8	1	1033.504	1235.534	107	334	0.75737	**0.015534**	78	499	0.864818	**0.012972**	501	1520	0.752103	**0.018992281**
N	y7	1	902.4631	1104.493	699	728	0.510161	**0.013519**	1010	1100	0.521327	**0.006906**	2860	2740	0.489286	**0.018236938**
AW	y6	1	788.4202	990.4502	509	213	0.295014	**0.012968**	731	374	0.338462	**0.008502**	2290	711	0.236921	**0.011560437**
V	y4	1	531.3038	733.3338	1460	142	0.088639	**0.00412**	2230	285	0.11332	**0.001758**	6070	506	0.076946	**0.003982325**
A	y3	1	432.2354	634.2654	1880	44.4	0.023072	**0.00145**	2810	201	0.066755	**0.002521**	7480	167	0.021839	**0.001578152**
WR	y2	1	361.1983	563.2283	137	0	0	**0**	233	0	0	**0**	866	0	0	**0**

Supplementary Table 28 – HEWL I98-R112 bound per-residue modification: table listing the raw data (ion intensity, and corresponding scan number and retention time (AD indicates aryldiazirine-modified peptide) and derived per residue modification

Sequence ID	Unbound Per-Residue Mod.					Bound Per-Residue Mod.					
	1	2	3	Average	St.Dev.	1	2	3	Average	St.Dev.	P-Value
I	0	0	0	0	0	0	0	0	0	0	
VS	0	0	0.003272	0.001091	0.001543	0	0	0	0	0	0.211325
DGNG	0.037637	0.030785	0.025447	0.03129	0.004989	0.015246	0.005105	0.017914	0.012755	0.005518	0.012187
M	0.050515	0.048113	0.038111	0.04558	0.005371	0.015534	0.012972	0.018992	0.015833	0.002467	0.002854
N	0.024238	0.030026	0.026567	0.026944	0.002378	0.013519	0.006906	0.018236	0.012887	0.004647	0.015917
AW	0.035643	0.030635	0.024409	0.030229	0.004595	0.012968	0.008502	0.011560	0.01101	0.001864	0.005973
V	0.001201	0.001723	0.000414	0.001113	0.000538	0.00412	0.001758	0.003982	0.003287	0.001082	0.042186
A	0.005	0.004012	0.002273	0.003762	0.001127	0.00145	0.002521	0.001578	0.00185	0.000478	0.057091
WR	0	0	0	0	0	0	0	0	0	0	

Supplementary Table 29 – HEWL I98-R112 summary: per residue modification (taken from above: 3 repeats + average and standard deviation with p values). Residues showing significant differences between bound and unbound are highlighted in red.

Sample					1				2				3			
Peptide Fr. Mod.					0.154234841				0.145294146				0.120495682			
Residue	Ion	Charge	m/z	Ad m/z	Peak Area	AD Peak Area	Fr.Mod	Per-residue mod.	Peak Area	AD Peak Area	Fr. Mod.	Per-residue mod.	Peak Area	AD Peak Area	Fr.Mod.	Per-residue mod.
I							1	**0**			1	**0**			1	**0**
VS	y14	2	781.8621	882.8771	0	70478	1	**0**	0	135546	1	**0**	0	119258	1	**0**
DGNG	y12	1	1376.616	1578.646	0	104694	1	**0.041109**	0	172363	1	**0.035542**	0	107773	1	**0.032300425**
M	y8	1	1033.504	1235.534	181704	500017	0.733463	**0.047117**	220268	680166	0.755376	**0.044122**	185792	507299	0.731937	**0.03549281**
N	y7	1	902.4631	1104.493	1299915	972562	0.427974	**0.023172**	1679443	1383563	0.451701	**0.026782**	1316953	1023799	0.43738	**0.025868203**
AW	y6	1	788.4202	990.4502	796586	306312	0.277734	**0.035247**	1072616	391452	0.267373	**0.032475**	885992	253839	0.222699	**0.023392217**
V	y4	1	531.3038	733.3338	2298711	118962	0.049205	**0.002583**	3066929	140680	0.043858	**0.002543**	2474635	72768	0.028566	**0.001125611**
A	y3	1	432.2354	634.2654	2850247	95611	0.032456	**0.005006**	3828076	103621	0.026355	**0.003829**	2957880	57977	0.019224	**0.002316416**
WR	y2	1	361.1983	563.2283	348298	0	0	**0**	467416	0	0	**0**	338347	0	0	**0**

Supplementary Table 30 – HEWL I98-R112 unbound per-residue modification calculated using PRM: table listing the extracted ion chromatogram peak area of each fragment and derived per residue modification.

Sample					1				2				3			
Peptide Fr. Mod.					0.062837084				0.037764366				0.072264222			
Residue	Ion	Charge	m/z	Ad m/z	Peak Area	AD Peak Area	Fr.Mod	Per-residue mod.	Peak Area	AD Peak Area	Fr. Mod.	Per-residue mod.	Peak Area	AD Peak Area	Fr.Mod.	Per-residue mod.
I							1	**0**			1	**0**			1	**0**
VS	y14	2	781.8621	882.8771	0	0	1	**0**	0	0	1	**0**	0	419751	1	**0**
DGNG	y12	1	1376.616	1578.646	0	0	1	**0.017151**	0	46276	1	**0.005869**	0	107567	1	**0.01283407**
M	y8	1	1033.504	1235.534	68289	181904	0.727055	**0.012953**	25789	140157	0.844594	**0.012148**	161402	747397	0.822401	**0.024167943**
N	y7	1	902.4631	1104.493	259809	282489	0.520911	**0.014771**	303752	332932	0.522916	**0.007192**	995721	948903	0.487962	**0.017865658**
AW	y6	1	788.4202	990.4502	181895	72803	0.28584	**0.011216**	216000	107578	0.332464	**0.00771**	761749	241523	0.240735	**0.011148121**
V	y4	1	531.3038	733.3338	459385	55248	0.107354	**0.004913**	635537	93548	0.128309	**0.002086**	1940000	183622	0.086466	**0.004426286**
A	y3	1	432.2354	634.2654	581022	17458	0.029171	**0.001833**	789656	62246	0.073067	**0.002759**	2409534	62328	0.025215	**0.001822142**
WR	y2	1	361.1983	563.2283	44868	0	0	**0**	68364	0	0	**0**	284222	0	0	**0**

Supplementary Table 31 – HEWL I98-R112 bound per-residue modification calculated using PRM: table listing the extracted ion chromatogram peak area of each fragment and derived per residue modification.

Sequence ID	Unbound Per-Residue Mod.					Bound Per-Residue Mod.					
	1	2	3	Average	St.Dev.	1	2	3	Average	St.Dev.	P-Value
I	0	0	0	0	0	0	0	0	0	0	
VS	0	0	0	0	0	0	0	0	0	0	
**DGNG**	0.041109	0.035542	0.0323	0.036317	0.003638	0.017151	0.005869	0.012834	0.011951	0.004648	0.002145
**M**	0.047117	0.044122	0.035493	0.042244	0.004928	0.012953	0.012148	0.024168	0.016423	0.005486	0.003876
**N**	0.023172	0.026782	0.025868	0.025274	0.001532	0.014771	0.007192	0.017866	0.013276	0.004484	0.034954
**AW**	0.035247	0.032475	0.023392	0.030372	0.005063	0.011216	0.00771	0.011148	0.010024	0.001637	0.016269
V	0.002583	0.002543	0.001126	0.002084	0.000678	0.004913	0.002086	0.004426	0.003808	0.001234	0.090829
A	0.005006	0.003829	0.002316	0.003717	0.001101	0.001833	0.002759	0.001822	0.002138	0.000439	0.07803
WR	0	0	0	0	0	0	0	0	0	0	

Supplementary Table 32 – HEWL D87-S100 per-residue modification summary: per residue modification calculated using PRM analysis (taken from above: 3 repeats + average and standard deviation with p values). Residues showing significant differences between bound and unbound are highlighted in red

Sample					1				2				3			
Averaged scans					580-743				533-626				563-682			
RT					25.27-28.89				26.7-30.08				25.65-29.16			
Peptide Fr. Mod.					0.122249503				0.254897953				0.133045856			
Residue	Ion	Charge	m/z	Ad m/z	Intensity	AD Intensity	Fr.Mod	Per-residue mod.	Intensity	AD Intensity	Fr. Mod.	Per-residue mod.	Intensity	AD Intensity	Fr.Mod.	Per-residue mod.
DVQ	b3	1	343.1612	545.1912	1410	0	0	**0**	215	0	0	**0**	506	0	0	**0**
A	b4	1	414.1983	616.2283	2610	0	0	**0**	431	0	0	**0**	905	0	0	**0**
W	b5	1	600.2776	802.3076	1320	20.8	0.015513	**0.001896472**	194	0	0	**0**	427	0	0	**0**
I	b6	1	713.3617	915.3917	610	32.4	0.050436	**0.004269287**	101	0	0	**0**	203	0	0	**0**
R	b7	1	869.4628	1071.493	2380	3000	0.557621	**0.062003108**	445	437	0.495465	**0.126292977**	806	1130	0.583678	**0.077655898**
GCRL							1	**0.054080635**			1	**0.128604976**			1	**0.055389959**

Supplementary Table 33 – HEWL D119-L129 unbound per-residue modification: table listing the raw data (ion intensity, and corresponding scan number and retention time (AD indicates aryldiazirine-modified peptide) and derived per residue modification

Sample					1				2				3			
Averaged scans					534-653				577-686				532-632			
RT					24.83-28.28				25.3-28.41				25.29-28.64			
Peptide Fr. Mod.					0.17422707				0.221429226				0.164908441			
Residue	Ion	Charge	m/z	Ad m/z	Intensity	AD Intensity	Fr.Mod	Per-residue mod.	Intensity	AD Intensity	Fr. Mod.	Per-residue mod.	Intensity	AD Intensity	Fr.Mod.	Per-residue mod.
DVQ	b3	1	343.1612	545.1912	990	0	0	**0**	1100	0	0	**0**	670	0	0	**0**
A	b4	1	414.1983	616.2283	1780	0	0	**0**	2100	0	0	**0**	1170	0	0	**0**
W	b5	1	600.2776	802.3076	910	0	0	**0**	964	29.8	0.029986	**0.006639757**	548	0	0	**0**
I	b6	1	713.3617	915.3917	411	0	0	**0**	604	0	0	**-0.006639757**	327	0	0	**0**
R	b7	1	869.4628	1071.493	1880	1810	0.490515	**0.085460975**	2450	2000	0.449438	**0.099518753**	1450	1200	0.45283	**0.07467552**
GCRL							1	**0.088766095**			1	**0.121910472**			1	**0.090232921**

Supplementary Table 34 – HEWL D119-L129 bound per-residue modification: table listing the raw data (ion intensity, and corresponding scan number and retention time (AD indicates aryldiazirine-modified peptide) and derived per residue modification

Sequence ID	Unbound Per-Residue Mod.					Bound Per-Residue Mod.					
	1	2	3	Average	St.Dev.	1	2	3	Average	St.Dev.	P-Value
DVQ	0	0	0	0	0	0	0	0	0	0	
A	0	0	0	0	0	0	0	0	0	0	
W	0.001896	0	0	0.000632	0.000894	0	0.00664	0	0.002213	0.00313	0.281547
I	0.004269	0	0	0.001423	0.002013	0	-0.00664	0	-0.00221	0.00313	0.130454
R	0.062003	0.126293	0.077656	0.088651	0.027373	0.085461	0.099519	0.074676	0.086552	0.010171	0.462725
GCRL	0.054081	0.128605	0.05539	0.079359	0.034827	0.088766	0.12191	0.090233	0.100303	0.01529	0.246453

Supplementary Table 35 – HEWL D119-L129 summary: per residue modification (taken from above: 3 repeats + average and standard deviation with p values).

Sample					1				2				3			
Peptide Fr. Mod.					0.122249503				0.254897953				0.133045856			
Residue	Ion	Charge	m/z	Ad m/z	Peak Area	AD Peak Area	Fr.Mod	Per-residue mod.	Peak Area	AD Peak Area	Fr. Mod.	Per-residue mod.	Peak Area	AD Peak Area	Fr.Mod.	Per-residue mod.
DVQ	b3	1	343.1612	545.1912	362000	14005	0.037247	**0.004553408**	50520	0	0	**0**	115801	0	0	**0**
A	b4	1	414.1983	616.2283	658602	3418	0.005163	**-0.003922236**	104023	0	0	**0**	211840	0	0	**0**
W	b5	1	600.2776	802.3076	368787	6342	0.016906	**0.0014356**	49686	0	0	**0**	108795	0	0	**0**
I	b6	1	713.3617	915.3917	210021	17207	0.075726	**0.007190657**	31545	0	0	**0**	64374	0	0	**0**
R	b7	1	869.4628	1071.493	632657	777200	0.551262	**0.058134025**	118287	111813	0.485932	**0.123863124**	213803	273192	0.560975	**0.074635394**
GCRL							1	**0.054858049**			1	**0.131034829**			1	**0.058410463**

Supplementary Table 36 – HEWL D119-L129 unbound per-residue modification calculated using PRM: table listing the extracted ion chromatogram peak area of each fragment and derived per residue modification.

Sample					1				2				3			
Peptide Fr. Mod.					0.17422707				0.221429226				0.164908441			
Residue	Ion	Charge	m/z	Ad m/z	Peak Area	AD Peak Area	Fr.Mod	Per-residue mod.	Peak Area	AD Peak Area	Fr. Mod.	Per-residue mod.	Peak Area	AD Peak Area	Fr.Mod.	Per-residue mod.
DVQ	b3	1	343.1612	545.1912	235864	0	0	**0**	233165	0	0	**0**	163240	0	0	**0**
A	b4	1	414.1983	616.2283	420085	0	0	**0**	464133	0	0	**0**	288849	0	0	**0**
W	b5	1	600.2776	802.3076	224597	0	0	**0**	213757	6511	0.029559	**0.006545325**	143367	0	0	**0**
I	b6	1	713.3617	915.3917	175907	0	0	**0**	216184	0	0	**-0.006545325**	113081	0	0	**0**
R	b7	1	869.4628	1071.493	468646	401989	0.461719	**0.080444004**	540824	429255	0.442495	**0.097981301**	350422	297075	0.458805	**0.075660853**
GCRL							1	**0.093783066**			1	**0.123447925**			1	**0.089247588**

Supplementary Table 37 – HEWL D119-L129 bound per-residue modification calculated using PRM: table listing the extracted ion chromatogram peak area of each fragment and derived per residue modification.

Sequence ID	Unbound Per-Residue Mod.					Bound Per-Residue Mod.					
	1	2	3	Average	St.Dev.	1	2	3	Average	St.Dev.	P-Value
DVQ	0.004553	0	0	0.001518	0.002146	0	0	0	0	0	0.211325
A	-0.00392	0	0	-0.00131	0.001849	0	0	0	0	0	0.211325
W	0.001436	0	0	0.000479	0.000677	0	0.006545	0	0.002182	0.003085	0.262699
I	0.007191	0	0	0.002397	0.00339	0	-0.00655	0	-0.00218	0.003085	0.115312
R	0.058134	0.123863	0.074635	0.085544	0.02792	0.080444	0.097981	0.075661	0.084695	0.009595	0.485631
GCRL	0.054858	0.131035	0.05841	0.081434	0.035103	0.093783	0.123448	0.089248	0.10216	0.015167	0.24959

Supplementary Table 38 – HEWL D119-L129 per-residue modification summary: per residue modification calculated using PRM analysis (taken from above: 3 repeats + average and standard deviation with p values).

Sequence ID	Peptide MW	m/z	AD m/z	Unlabelled PA 1	AD PA 1	Unlabelled PA 2	AD PA 2	Unlabelled PA3	AD PA3	Fr.Mod.			Average	St.Dev.
										**1**	**2**	**3**		
**I363-K381**	2107.02	1054.53	1155.54	283921938	55636655	140555069	24972822	59883740	6517796	0.163850	0.150868	0.098157	**0.137625**	**0.028407**
**D136-R146**	1110.60	556.31	657.33	162679901	53416601	109051322	27148493	26635533	11998790	0.247189	0.199328	0.310573	**0.252363**	**0.045563**
**L101-K115**	1562.78	782.4	883.42	78107529	21377093	22791918	6318202	1829170	611984	0.214878	0.217045	0.250695	**0.227539**	**0.016397**
**D825-R834**	1336.65	669.33	770.35	149997089	3171938	102679910	2051690	36790306	971006	0.020709	0.019590	0.025714	**0.022004**	**0.002663**
**V149-R164**	1575.81	788.92	889.93	223977625	46784756	111509513	20524470	22581200	5271013	0.172789	0.155448	0.189249	**0.172496**	**0.013801**
**G606-K630**	2598.31	1300.17	1401.19	130519033	28043172	80421960	15148686	12787574	2660458	0.176859	0.158508	0.172220	**0.169196**	**0.007791**
**F578-K587**	1208.62	605.32	706.34	135814646	23105822	111772616	14250563	73969100	16712828	0.145392	0.113079	0.184302	**0.147591**	**0.029118**
**I124-R135**	1413.82	707.92	808.94	331119067	144351321	247521458	74752672	79501276	19373372	0.303597	0.231954	0.195939	**0.243830**	**0.044746**
**L589-R605**	1986.00	994.02	1095.04	46339381	0	12042358	0	3532795	0	0.000000	0.000000	0.000000	**0.000000**	**0**
**V485-K501**	1916.97	959.5	1060.51	31438891	1608651	4885223	0	2147731	0	0.048677	0.000000	0.000000	**0.016226**	**0.022946**
**F564-K576**	1539.83	770.93	871.95	372599550	29358807	144441183	8436403	90144710	2912126	0.073039	0.055184	0.031294	**0.053173**	**0.017102**
**E208-R223**	1859.92	930.98	1031.99	10768130	0	6170741	0	8429336	0	0.000000	0.000000	0.000000	**0.000000**	**0**
**I306-R331**	2855.52	952.86	1020.2	105796077	57194764	78895776	45547431	100818369	31891866	0.350908	0.366010	0.240312	**0.319077**	**0.056035**
**A734-R757**	2675.22	892.76	960.1	26969770	28048561	17496558	19779828	21095462	22554675	0.509804	0.530626	0.516715	**0.519048**	**0.008659**
**L250-K287**	4178.93	1045.76	1096.27	8335304	16926710	6822938	12242181	16864769	25713085	0.670046	0.642125	0.603907	**0.638693**	**0.02711**
**G1-R19**	2015.06	1008.55	1109.57	16814448	14232931	16056187	10551798	9583245	7293292	0.458426	0.396565	0.432156	**0.429049**	**0.02535**
**Q435-R451**	2117.04	706.69	774.04	74855173	39249282	47036584	24124191	55492462	12306916	0.343977	0.339010	0.181520	**0.288169**	**0.07544**
**N332-R354**	2580.33	861.13	928.48	48947831	22395951	38792894	16167203	71640261	14378679	0.313916	0.294163	0.167157	**0.258412**	**0.065029**
**T294-R305**	1479.66	740.84	841.86	6501101	20815171	4612906	6437692	1576470	7459689	0.762006	0.582565	0.825538	**0.723370**	**0.102887**

Supplementary Table 39 – Unbound USP5 peptide level modification: table listing all peak area values of each extracted ion chromatogram of each tryptic peptide obtained from digestion of the protein footprinted in the unbound state.

Sequence ID	Peptide MW	m/z	AD m/z	Unlabelled PA 1	AD PA 1	Unlabelled PA 2	AD PA 2	Unlabelled PA3	AD PA3	Fr.Mod.			Average	St.Dev.
										1	2	3		
**I363-K381**	2107.02	1054.53	1155.54	638415002	28498706	126200267	18793553	576547739	18678092	0.042732	0.129616	0.03138	**0.067909**	**0.043879**
**D136-R146**	1110.60	556.31	657.33	229727344	39206871	75495204	31459748	175656271	34721232	0.145786	0.29414	0.165043	**0.201656**	**0.065867**
**L101-K115**	1562.78	782.4	883.42	51298959	11682520	13176634	6296681	32312873	13991163	0.185491	0.323349	0.302159	**0.270333**	**0.060613**
**D825-R834**	1336.65	669.33	770.35	250716330	1951749	94252454	2217579	200664461	2713961	0.007725	0.022987	0.013344	**0.014685**	**0.006303**
**V149-R164**	1575.81	788.92	889.93	233429431	46535740	83434008	24771339	165388466	35884532	0.16622	0.228929	0.178288	**0.191146**	**0.027167**
**G606-K630**	2598.31	1300.17	1401.19	207039816	30522104	104011698	17398613	369102942	54878965	0.128481	0.143304	0.129437	**0.133741**	**0.006774**
**F578-K587**	1208.62	605.32	706.34	197779718	18573057	135606514	14992660	185211671	12293291	0.085846	0.099553	0.062243	**0.082548**	**0.015409**
**I124-R135**	1413.82	707.92	808.94	475019209	136651227	278443163	89764734	358714667	61120319	0.223407	0.243788	0.145582	**0.204259**	**0.042317**
**L589-R605**	1986.00	994.02	1095.04	44772905	0	20207461	0	93728519	0	0	0	0	**0**	**0**
**V485-K501**	1916.97	959.5	1060.51	29298399	0	8875853	589648	84049396	0	0	0.062294	0	**0.020765**	**0.029366**
**F564-K576**	1539.83	770.93	871.95	407030042	9249409	212560823	7174385	393279957	4804199	0.022219	0.03265	0.012068	**0.022313**	**0.008403**
**E208-R223**	1859.92	930.98	1031.99	10498861	0	4600261	0	46921683	0	0	0	0	**0**	**0**
**I306-R331**	2855.52	952.86	1020.2	331332031	62572967	204031607	50948445	366451556	43080915	0.158853	0.199813	0.105195	**0.154621**	**0.038743**
**A734-R757**	2675.22	892.76	960.1	30226612	34951577	23644710	24438729	29981414	34647070	0.536247	0.508257	0.536096	**0.526866**	**0.013159**
**L250-K287**	4178.93	1045.76	1096.27	100658537	19659305	43232275	17424984	355183296	57111066	0.163395	0.28727	0.13852	**0.196395**	**0.065056**
**G1-R19**	2015.06	1008.55	1109.57	33976455	23041599	17870832	14649604	41831181	30676289	0.404111	0.450474	0.423078	**0.425887**	**0.019032**
**Q435-R451**	2117.04	706.69	774.04	310978690	20875895	129304602	18852692	286837473	13413276	0.062907	0.127248	0.044674	**0.078276**	**0.035419**
**N332-R354**	2580.33	861.13	928.48	183686476	16306936	100277467	19396391	135393202	7974522	0.081537	0.162077	0.055623	**0.099746**	**0.045327**
**T294-R305**	1479.66	740.84	841.86	25380952	14792950	6556155	7933959	13800383	22406018	0.368223	0.547543	0.618841	**0.511536**	**0.105435**

Supplementary Table 40 – Diubiquitin bound USP5 peptide level modification: table listing all peak area values of each extracted ion chromatogram of each tryptic peptide obtained from digestion of the protein footprinted in the bound state.

Sequence ID	Unbound Fractional Mod.					Bound Fractional Mod.					P-Value
	1	2	3	Average	St.Dev.	1	2	3	Average	St.Dev.	
I363-K381	0.16385	0.150868	0.098157	0.137625	0.028407	0.042732	0.129616	0.03138	0.067909	0.043879	0.155742
D136-R146	0.247189	0.199328	0.310573	0.252363	0.045563	0.145786	0.29414	0.165043	0.201656	0.065867	0.421196
L101-K115	0.214878	0.217045	0.250695	0.227539	0.016397	0.185491	0.323349	0.302159	0.270333	0.060613	0.43683
D825-R834	0.020709	0.01959	0.025714	0.022004	0.002663	0.007725	0.022987	0.013344	0.014685	0.006303	0.22754
V149-R164	0.172789	0.155448	0.189249	0.172496	0.013801	0.16622	0.228929	0.178288	0.191146	0.027167	0.450406
G606-K630	0.176859	0.158508	0.17222	0.169196	0.007791	0.128481	0.143304	0.129437	0.133741	0.006774	0.008299
F578-K587	0.145392	0.113079	0.184302	0.147591	0.029118	0.085846	0.099553	0.062243	0.082548	0.015409	0.068296
I124-R135	0.303597	0.231954	0.195939	0.243830	0.044746	0.223407	0.243788	0.145582	0.204259	0.042317	0.414926
L589-R605	0	0	0	0.000000	0	0	0	0	0	0	
V485-K501	0.048677	0	0	0.016226	0.022946	0	0.062294	0	0.020765	0.029366	0.871605
F564-K576	0.073039	0.055184	0.031294	0.053173	0.017102	0.022219	0.03265	0.012068	0.022313	0.008403	0.105917
E208-R223	0	0	0	0.000000	0	0	0	0	0	0	
**I306-R331**	**0.350908**	**0.36601**	**0.240312**	**0.319077**	**0.056035**	**0.158853**	**0.199813**	**0.105195**	**0.154621**	**0.038743**	**0.026929**
A734-R757	0.509804	0.530626	0.516715	0.519048	0.008659	0.536247	0.508257	0.536096	0.526866	0.013159	0.533311
**L250-K287**	**0.670046**	**0.642125**	**0.603907**	**0.638693**	**0.02711**	**0.163395**	**0.28727**	**0.13852**	**0.196395**	**0.065056**	**0.003016**
G1-R19	0.458426	0.396565	0.432156	0.429049	0.02535	0.404111	0.450474	0.423078	0.425887	0.019032	0.894646
**Q435-R451**	**0.343977**	**0.33901**	**0.18152**	**0.288169**	**0.07544**	**0.062907**	**0.127248**	**0.044674**	**0.078276**	**0.035419**	**0.037775**
**N332-R354**	**0.313916**	**0.294163**	**0.167157**	**0.258412**	**0.065029**	**0.081537**	**0.162077**	**0.055623**	**0.099746**	**0.045327**	**0.047308**
T294-R305	0.762006	0.582565	0.825538	0.723370	0.102887	0.368223	0.547543	0.618841	0.511536	0.105435	0.111758

Supplementary Table 41 –USP5 peptide level modification summary: table listing per-peptide fractional modification values obtained from peak area values derived from unbound and bound USP5 (shown in Supplementary Tables 34 and 35). Entries in red include pepides showing significant differences.

Supplementary Methods

Determination of the Extent of Protein Labelling

The method proposed by Delfino et al.[1] was used to calculate the extent of labeling per protein (EL) obtained with the commercial photoleucine **1** and our aryldiazirine **2**. The spectra obtained for each labelled protein were deconvoluted using the Xtract algorithm (Thermo Xcalibur software) and the extent of labelling was calculated using the Supplementary Equation 1

$$EL = \sum_{i=0}^{i=n} iI_i \Big/ \sum_{i=}^{i=n} I_i \qquad (1)$$

where I_i is the sum of the intensities of all the peaks for each species *i* including oxidation products and *i* is the index indicating the number of labels inserted per protein.

Data Analysis for Peptide Labeling

***Peptide level analysis*.** The method proposed by Jumper *et al.* was used to evaluate the amount of labelling on each peptide and on single residues. The chromatograms for each singly-labelled and unlabelled peptide were extracted within a range of ±0.1 m/z and the spectrum for each peak was manually inspected to ensure the sampling of the correct ion only. In the case of lysozyme, these data were derived from the set of analysis files that contained one full scan and one MS/MS acquisition channel. In the few cases where there was an interfering signal in the same mass window as the selected peptide, a smaller mass tolerance or the second isotope of the peak envelope was selected for the extraction of the

ion chromatogram. The peptide fractional modification was obtained calculating the ratio between the peak area of the labelled form and the sum of the peak areas of labelled and unlabelled forms (Supplementary Equation 2).

$$P = \frac{A_{\text{labelled}}}{A_{labelled} + A_{\text{unlabelled}}} \quad (2)$$

The fractional modification values obtained for each peptide derived from bound and unbound proteins were respectively averaged and compared in order to determine the difference in the reaction extent. Differences in the extent of labelling between peptides were considered significant when the p-value obtained from a Student t-test was <0.05

***Residue level analysis*.** Modification sites on the peptides were investigated using tandem MS. On every mono-labelled peptide the label is distributed over the sequence and after undergoing fragmentation it will generate labelled and unlabeled fragments. The data has been averaged over all the chromatographic features of the precursor and the ratio between the intensity of the $n_{i\ \text{labelled}}$ fragment and the sum of the intensities of $n_{i\ \text{labelled}}$ and $n_{i\ \text{unlabelled}}$ allows the calculation of the fractional modification on the n_i residue (Supplementary Equation 3).

$$f.mod\ (n_{\text{i}}) = \frac{I(n_{\text{i labelled}})}{I(n_{\text{i labelled}}) + I(n_{\text{i unlabelled}})} \quad (3)$$

The difference in fractional modification between two consecutive fragments multiplied by the fractional amount of labelling on the peptide will give the absolute level of modification on the n_i residue (Supplementary Equation 4).

$$abs.mod. = P[f.mod.(n_i) - f.mod.(n_{i-1})] \quad (4)$$

In the case of the impossibility of detection of a n_i ion, its fractional modification was grouped together with its subsequent fragment.

The same mathematical approach was used to validate the results using parallel reaction monitoring (PRM). Extracted ion chromatograms (XIC) of each fragment were generated allowing a mass tolerance of ±0.1 Da and manually inspected to ensure the sampling of the correct ion. XICs were then integrated and the peak area values obtained for each fragment were used to calculate the fractional modification and the absolute modification using the methodology described above.

The few fractional modification values of residues that showed an anomalous increase or decrease in respect to the fractional modification curve trend were excluded from the dataset and were grouped together with the value corresponding to the adjacent residue resulting in only a small loss of resolution. The phenomenon appeared mostly on fragments corresponding to cleavage of the peptide bond of serine, threonine and cysteine residues for reasons yet to be investigated.

Per-residue modification values from each repeat were averaged and the averages compared between the two states of the protein. Differences in per-residue modification were considered significant when the p-value obtained from a Student t-test was <0.05 and the difference in label/residue was >0.01

USP5 AD peptides ID

Bioworks software was used to identify the peptides searching a custom FASTA database that included the sequence of the protein and allowed for the 202.02 ±0.02 amu modification on all residues. The spectra shown are extracted from the software except for the L250-K287 peptide, which was assigned manually. MS/MS spectra for the 4 remaining low abundance peptides were not recorded, but their identities were deduced from the predicted mass shift and relative chromatographic retention.

Supplementary References

1. Gomez, G.E., Mundo, M.R., Craig, P. O. & Delfino, J.M. Probing Protein Surface with a Solvent Mimetic Carbene Coupled to Detection by Mass Spectrometry. *J. Am. Soc. Mass Spectrom.* **23**, 30-42 (2012).
